# Climate change may threaten habitat suitability of threatened plant species within Chinese nature reserves

**DOI:** 10.7717/peerj.2091

**Published:** 2016-06-14

**Authors:** Chunjing Wang, Chengzhu Liu, Jizhong Wan, Zhixiang Zhang

**Affiliations:** School of Nature Conservation, Beijing Forestry University, Beijing, China

**Keywords:** Climate change, Threatened plant species, Conservation areas, Suitable habitat, China, Schoener's *D*, Maxent modelling

## Abstract

Climate change has the potential to alter the distributions of threatened plant species, and may therefore diminish the capacity of nature reserves to protect threatened plant species. Chinese nature reserves contain a rich diversity of plant species that are at risk of becoming more threatened by climate change. Hence, it is urgent to identify the extent to which future climate change may compromise the suitability of threatened plant species habitats within Chinese nature reserves. Here, we modelled the climate suitability of 82 threatened plant species within 168 nature reserves across climate change scenarios. We used Maxent modelling based on species occurrence localities and evaluated climate change impacts using the magnitude of change in climate suitability and the degree of overlap between current and future climatically suitable habitats. There was a significant relationship between overlap with current and future climate suitability of all threatened plant species habitats and the magnitude of changes in climate suitability. Our projections estimate that the climate suitability of more than 60 threatened plant species will decrease and that climate change threatens the habitat suitability of plant species in more than 130 nature reserves under the low, medium, and high greenhouse gas concentration scenarios by both 2050s and 2080s. Furthermore, future climate change may substantially threaten tree plant species through changes in annual mean temperature. These results indicate that climate change may threaten plant species that occur within Chinese nature reserves. Therefore, we suggest that climate change projections should be integrated into the conservation and management of threatened plant species within nature reserves.

## Introduction

Climate change is predicted to become a major threat to biodiversity in the 21st century, forcing plant species distributions to shift or decrease dramatically (Thuiller et al., 2005; Bellard et al., 2012; Corlett & Westcott, 2013). When the suitable habitats of plant species shift outside of the range to which plant species are adapted, these plant species face an increased risk of extinction (Thuiller et al., 2005; Summers et al., 2012). Extinction risk evaluations have been completed for woody plant species, projecting declines of many species ranges under climate change (Zhang et al., 2014). Nature reserves play an important role in the conservation of threatened plant species worldwide (Hansen et al., 1991; Xu & Melick, 2007). The establishment of nature reserves is one of the most effective methods available for conserving plant habitats and slowing plant species population declines (Saetersdal, Line & Birks, 1993; Araújo et al., 2011; Ma et al., 2013). However, climate change may affect the ability of nature reserves to protect threatened plant species and even cause extinctions of threatened plant species protected within nature reserves (Araújo et al., 2004; Araújo et al., 2011). Climate change has already been shown to endanger plant diversity in European conservation areas (Thuiller et al., 2005; Araújo et al., 2011). The ability of nature reserves to protect threatened tree plants in northeastern China under climate change was recently assessed using projected changes in species distributions (Yu et al., 2014). As plant species are already vulnerable to extinction within nature reserves, assessing the effects of continued climate change on plant distributions is essential. Specifically, climate change assessments must be integrated into the conservation management plans for threatened plant species in nature reserves based on the effects of climate change on the distributions of plant species and habitat suitability (Groves et al., 2012; Lawson et al., 2012; Fordham et al., 2013).

Recent research has evaluated the effect of climate change on threatened plant species in nature reserves using ecological niche models (ENMs; Yu et al., 2014; Wan et al., 2014; Wang et al., 2015). ENMs are a popular tool used to model climate suitability or potential distributions of plant species based on species occurrence data and environmental variables across current species ranges (Elith et al., 2011; Merow, Smith & Silander, 2013). The changes in species distributions that can be inferred with ENMs, such as future projections based on climate change, are an important tool for extinction assessment of threatened plant species (Araújo et al., 2011; Fordham et al., 2012). However, there are many challenges in applying ENMs to the conservation of plant species. Plants have limited seed dispersal and migration distances, hindering large-scale movement that might be necessary for species to survive climate change (McConkey et al., 2012, Corlett & Westcott, 2013; Iverson & McKenzie, 2013). Hence, ENMs can underestimate or overestimate future plant species distributions based on future climatic suitability as estimated by ENMs (Iverson & McKenzie, 2013; Zhang et al., 2014). Thus, we may not be able to determine effective conservation plans for threatened plant species in nature reserves under climate change in this way, which undermines the apparent usefulness of ENM assessments for threatened plant species (Hijmans & Graham, 2006; Aranda & Lobo, 2011; Pineda & Lobo, 2012). To improve the usefulness of ENMs in conservation management, we evaluated changes in habitat suitability for threatened plant species based on the current occurrences of plant populations rather than potential suitable habitats estimated from ENMs (Pineda & Lobo, 2012).

China contains rich plant diversity, including more than 10% of the world's vascular plant species owing to its large area (9.6 million km^2^) and high environmental heterogeneity, which encompasses boreal, temperate, subtropical, and tropical biomes (Liu & Diamond, 2005; Ren et al., 2007; Yang, Ma & Kreft, 2014). Furthermore, China harbors more threatened plant species than many other regions worldwide (Liu & Diamond, 2005; Wu et al., 2011). However, Chinese nature reserves only cover 27.5% of threatened plant species distributions (Zhang et al., 2015). Moreover, climate change poses a considerable threat to plant species in China (Wang et al., 2015).

Here, we examined the effects of climate change on threatened plant species within nature reserves by assessing changes in climate suitability based on occurrence localities of species compiled from previous field work. In this study, we used Maxent modelling to project the distributions of 82 threatened Chinese plant species from four plant types and distributed among 168 nature reserves. To accomplish this, we fulfilled two goals: (1) the assessment of changes in climate suitability ranges for threatened plants in the future and (2) the evaluation of the overlap between current and future climate suitability ranges. Finally, we suggest several effective approaches for the conservation of threatened plants in the context of climate change.

## Methods

### Species data and occurrence locality data

We selected threatened plant species from the List of National Key Protected Wild Plants approved by the State Council of China (http://www.gov.cn/gongbao/content/2000/content_60072.htm). We obtained the geographical coordinates of occurrence localities within national nature reserves from 168 scientific research reports finished after 1990, drawing our nature reserve samples from all provinces of China except Hong Kong, Macao, Shanghai, Tianjing, and Taiwan. The list of on the threatened plant species within these national nature reserves was drawn in Table S1. We obtained 4,982 records of 82 threatened plant species from within the 168 nature reserves, with each species having at least 10 recorded occurrences to ensure satisfactory performance of ENMs (Table S1; Pearson et al., 2007; Wang et al., 2015). We grouped 82 threatened plant species based on plant type such as tree, shrub, herb, and fern species using the reference *Rare and Endangered Plants in China* (China's State Forestry Administration and the Institute of Botany, Chinese Academy of Sciences, 2013; Table S1).

### Environmental variables

We obtained spatial data on 32 environmental variables at a 10-arc-min resolution including nine soil (http://soilgrids.org/), three topography (http://www.worldclim.org/), one wilderness (http://due.esrin.esa.int/page_globcover.php), and nineteen climate variables (http://www.worldclim.org/; Table S2). We tested for multi-collinearity amongst variables using Pearson correlation coefficients from a principal component analysis. Using the scores from the first two principal components (cumulative percentage, 58.614%), we excluded variables with a cross-correlation coefficient absolute value exceeding 0.75 (Tables S2 and S3; Farashi & Najafabadi, 2015). This reduced our predictor variable set to 17 environmental variables that may influence the distribution and physiological performance of threatened plant species and can therefore be used in ENMs to infer the current climate suitability of threatened plant species (Tables S2 and S3; Wang et al., 2015).

We obtained the same bioclimatic variables as Table S2 for our future projections. To model the future climate suitability for threatened plant species in roughly the 2050s (i.e., 2040--2069) and 2080s (i.e., 2070--2099), we used the average projection maps generated under four global climate models (i.e., bcc_csm1_1, csiro_mk3_6_0, gfdl_cm3, and mohc_hadgem2_es) and three greenhouse gas concentration scenarios as representative concentration pathways (RCPs) of 2.6 (mean, 270 ppm; range, 140--410 by 2100), 4.5 (mean, 780 ppm; range, 595--1,005 by 2100), and 8.5 (mean, 1,685 ppm; range, 1,415--1,910 by 2100), representing the low, medium, and high gas concentration scenarios, respectively (http://www.ipcc.ch/; http://www.ccafs-climate.org/). We used these three RCPs to represent the low, medium and high emission climate scenarios in order to estimate the future climate suitability for threatened plant species (http://www.ipcc.ch/). Our projections keep the non-climatic variables constant into the future, with only the climate variables changing.

### Modelling habitat suitability of species

We used Maxent modelling to predict the climatically suitable habitats for the 82 threatened plant species using occurrence localities and bioclimatic variables. Maxent is currently one of the most frequently applied ENMs (Merow, Smith & Silander, 2013). We optimized the analysis settings based on previous work by Merow, Smith & Silander (2013) and set the regularization multiplier (i.e., *beta*) to 1.5 to produce smooth and general response curves that represent a biologically realistic model (Tingley et al., 2014). The maximum number of background points was set to 10,000. A 5-fold cross-validation approach for testing was employed to remove bias with respect to recorded occurrence points (Wang et al., 2015). All other settings were as described by Merow, Smith & Silander (2013). We evaluated the predictive precision of Maxent using the area under the curve (AUC) of the receiver operation characteristic (ROC). AUC values range from 0.5 (i.e., lowest predictive ability or occurrences exhibiting no difference from randomly selected background points) to 1 (i.e., highest predictive ability). Models of each species with cross-validation testing AUC values above 0.7 were considered useful in our study (Elith et al., 2011; Merow, Smith & Silander, 2013). The logistic output format provided by Maxent assigns each map grid cell a value of 0--1, with 0 representing the lowest environmental suitability for a species and 1 the highest (Merow, Smith & Silander, 2013).

We tested the effects of environmental variables on the habitat suitability for threatened plant species using permutation importance (PI) and percentage contribution (PC) based on the jackknife method. PI evaluates the change in model AUC scores when each predictor was randomly permuted. A variable is considered important when AUC scores decrease substantially. PCs represent the influence of a particular environmental variable on the final model; the sum of all the variables is 100%. The threshold PC of habitat suitability for each species was 15% (Oke & Thompson, 2015). First, we computed the average PI values based on the different groups of plant types (Oke & Thompson, 2015). Second, we analyzed the effect of environmental variables on habitat suitability based on the proportion of total plant species affected according to the PC results (at a 15% threshold) and for different groups of plant types (Oke & Thompson, 2015). Finally, we used a linear regression to determine the relationship between the average PI values and the proportion of the total plant species affected using the PC results broken down into the categories of trees, shrubs, herbs, and ferns.

### Climatic habitat suitability analysis

To ensure proper model performance in our study, we evaluated the climate suitability for threatened plant species with occurrence localities based on previous field work (Pineda & Lobo, 2012; Van Andel et al., 2015; Walsh & Haseeb, 2015). We used ArcGIS 10.2 (Esri; Redlands, CA, USA) to extract the current and future climate suitability for threatened plant species based on occurrence localities from the maps of climate suitability generated by our Maxent models. Occurrence localities were derived from field data coded as presence and absence within nature reserves. We then used two indices: (1) changes in climate suitability in order to identify climate suitability of threatened plant species and (2) the overlap between current and future climatically suitable habitats under the low, medium and high concentration scenarios. The species with substantially decreasing climate suitability and large overlaps between current and future climatically suitable habitats indicate highly negative effects of climate change on habitat suitability (Thuiller et al., 2005; Keith et al., 2008). The projected changes in climate suitability may indicate variability in the potential locations of suitable climate conditions for threatened plant species in China, and the overlap between current and future climatically suitable habitats may indicate the potential movement of suitable climate conditions for threatened plant species (Warren, Glor & Turelli, 2008; Groom, 2013; Guisan et al., 2014).

We used ArcGIS 10.2 (Esri, Redlands, CA, USA) to calculate the change in climate suitability (*C*) between current conditions and those projected for the 2050s and 2080s (under the low, medium, and high concentration scenarios, respectively; Yu et al., 2014). We used the following equation to estimate *C*:

\begin{eqnarray*}C= \frac{A-B}{B} \end{eqnarray*}

where *C* is the change in the climate suitability for threatened plant species based on either the occurrence localities of each threatened plant species across all the nature reserves or of all the plants belonging to each nature reserve independently, and *A* and *B* are the future and current average climate suitability of individual grid cells based on the occurrence localities of each threatened plant species across all the nature reserves or of all the plants belonging to each nature reserve independently.

We used Schoener's *D* to compute the overlap between current and future climate suitability of threatened plant species based on the occurrence localities of each plant across all nature reserves as well as all the plant species belonging to each nature reserve (Warren, Glor & Turelli, 2008; Rödder & Engler, 2011). *D* is an ideal method for computing niche overlap from climate-based ENMs (Rödder & Engler, 2011). Here, we computed *D* in ENMtools 1.4.4 with values ranging from 0 (species that have completely discordant climate niches) to 1 (species that have identical climate niches; Warren, Glor & Turelli, 2008; Warren, Glor & Turelli, 2010). Detailed information on the *D* statistic is provided by Warren, Glor & Turelli (2008) and Warren, Glor & Turelli (2010).

First, we used a linear regression to explore the relationships between *C* and *D* based on occurrence localities of each threatened plant species in all the nature reserves and of all the plants belonging to each nature reserve under the low, medium, and high greenhouse gas concentration scenarios (in both the 2050s and 2080s). We projected a substantial change in habitat suitability between current and future concentration scenarios producing a large gap between current and future climatically suitable habitats of threatened plant species. Hence, we first focused on the change in climate suitability (*C*) between current conditions and those of the 2050s and 2080s based on occurrence localities of each species across all nature reserves and of all the threatened plants belonging to each nature reserve individually. Second, we computed the average values of *C* for trees, shrubs, herbs, and ferns as groups to determine the change range of *C* for different types of plants. Finally, we used a non-parametric test to explore differences in *C* among all plants belonging to each nature reserve and for different plant type groups across all the nature reserves between the low, medium, and high greenhouse gas concentration scenarios.

## Results

For all 82 threatened plant species across 168 nature reserves, model performance assessed using AUC scores was high (all models had AUC values over 0.7; Table S1). There were significant relationships between PI values and PC estimates from Maxent modelling (Fig. S1; *P* < 0.001) indicating that the variables selected by a jackknife test typically have consistent and high PC and PI values for tree, shrub, herb, and fern species. The largest effect on habitat suitability for trees (PI, 24.27; PC, 41%), herbs (PI, 22.52; PC, 25%), and ferns (PI, 19.32; PC, 38%) was produced by annual mean temperature changes, and precipitation seasonality most strongly impacted the habitat suitability of shrubs (PI, 17.21; PC, 33%; Table 1). For non-climatic variables, we found that soil pH was the important variable influencing habitat suitability for shrubs (PI, 16.65; PC, 50%) and ferns (PI, 16.82; PC, 25%). Specifically, the most important variables determined in this study were annual mean temperature for *Malania oleifera* (a tree; PI, 95.615), precipitation seasonality for *Platycrater arguta* (a shrub; PI, 88.711), and soil pH for *Alsophila gigantea* (a fern; PI, 90.218; Table S4). In addition, we found that temperature seasonality strongly affects habitat suitability for *Magnolia wilsonii* (a shrub; PI, 92.327) and that mean diurnal range has an important impact on habitat suitability for *Fokienia hodginsii* (a tree; PI, 61.271; Table S4).

**Table 1:** Summary of the permutation importance (PI) and percentage contribution (PC; %) for each plant type. The values (plus or minus standard errors) represent average PI, and the values inside the parentheses represent the percentage of the total plant species impacted based on the PC results. The codes of variables were the same as Table S2.

Variables	Tree	Shrub	Herb	Fern
BLD	0.72 ± 0.13(0)	0.20 ± 0.18(0)	0.76 ± 0.19(0)	0.95 ± 0.74(0)
CEC	0.60 ± 0.19(0)	0.30 ± 0.27(0)	0.50 ± 0.23(0)	0.51 ± 0.30(0)
CLYPPT	1.35 ± 0.37(0)	3.50 ± 2.13(0)	2.24 ± 1.07(0)	1.62 ± 0.87(0)
CRFVOL	0.69 ± 0.16(0)	0.49 ± 0.28(0)	0.97 ± 0.33(0)	1.33 ± 0.73(0)
OCSTHA	1.07 ± 0.31(2)	0.81 ± 0.55(0)	0.72 ± 0.25(0)	1.41 ± 0.45(0)
PHIHOX	4.72 ± 1.18(9)	16.65 ± 6.68(50)	2.37 ± 1.37(0)	16.82 ± 10.10(25)
SLTPPT	0.36 ± 0.09(0)	0.23 ± 0.17(0)	2.93 ± 1.68(0)	4.36 ± 3.77(0)
SNDPPT	0.56 ± 0.13(0)	0.25 ± 0.17(0)	0.98 ± 0.37(0)	0.25 ± 0.24(0)
Aspect	0.58 ± 0.10(0)	0.41 ± 0.19(0)	0.82 ± 0.31(0)	1.44 ± 0.69(0)
Slope	2.50 ± 0.70(14)	1.36 ± 0.66(0)	2.56 ± 1.07(16)	3.95 ± 0.95(0)
Globcover	1.14 ± 0.21(0)	2.28 ± 2.00(0)	0.72 ± 0.28(0)	0.88 ± 0.45(0)
Bio1	24.27 ± 3.13(41)	5.15 ± 2.42(33)	22.52 ± 5.74(25)	19.32 ± 8.42(38)
Bio2	9.77 ± 1.99(39)	8.88 ± 7.76(33)	2.63 ± 1.42(25)	18.60 ± 7.86(38)
Bio3	7.75 ± 1.39(5)	2.72 ± 1.61(0)	12.50 ± 3.31(33)	0.71 ± 0.43(0)
Bio4	18.42 ± 3.02(16)	23.57 ± 13.69(33)	20.03 ± 4.25(42)	8.21 ± 3.98(0)
Bio12	11.60 ± 2.04(71)	15.99 ± 13.29(33)	11.60 ± 4.01(42)	17.37 ± 6.76(63)
Bio15	13.90 ± 2.86(18)	17.21 ± 9.38(33)	15.15 ± 4.13(8)	2.26 ± 0.45(0)

**Figure 1:**
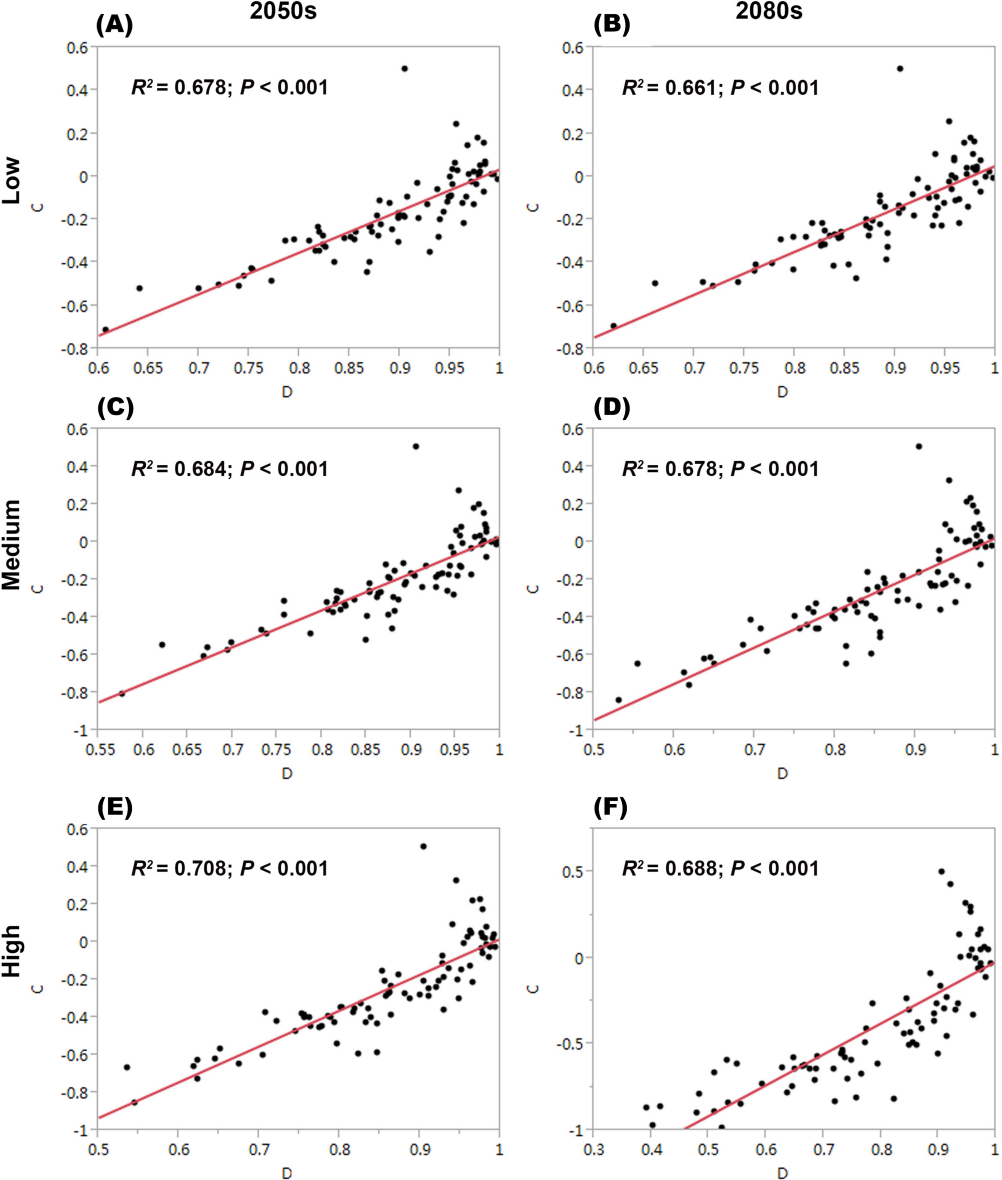
Relationships of the congruence between current and future climate suitability of threatened plant species with changes in climate suitability in all nature reserves under the low, medium, and high greenhouse gas concentration scenarios by both (A, C, E, respectively) the 2050s and (B, D, and F, respectively) the 2080s. *C* represents the changes in the climatic habitat suitability for threatened plant species. *D* represents the overlap between current and future climatic habitat suitability of threatened plant species in nature reserves.

**Figure 2:**
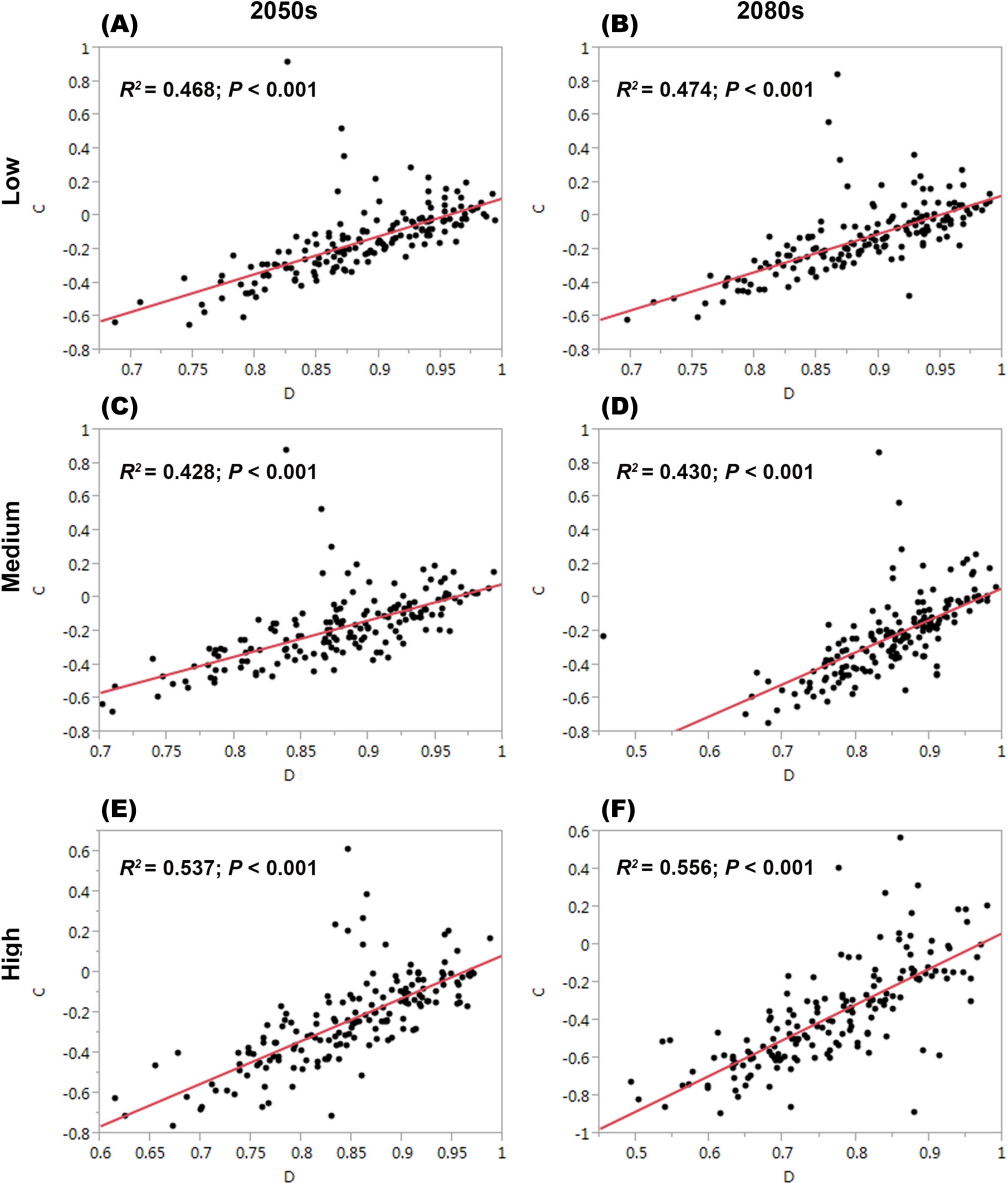
Relationships of the congruence between current and future climate suitability of threatened plant species with changes in climate suitability for all threatened plant species belonging to each nature reserve under the low, medium, and high greenhouse gas concentration scenarios for both (A, C, and E, respectively) the 2050s and (B, D, and F, respectively) the 2080s. *C* represents the changes in the climatic habitat suitability for threatened plant species. *D* represents the overlap between current and future climatic habitat suitability of threatened plant species in nature reserves.

**Figure 3:**
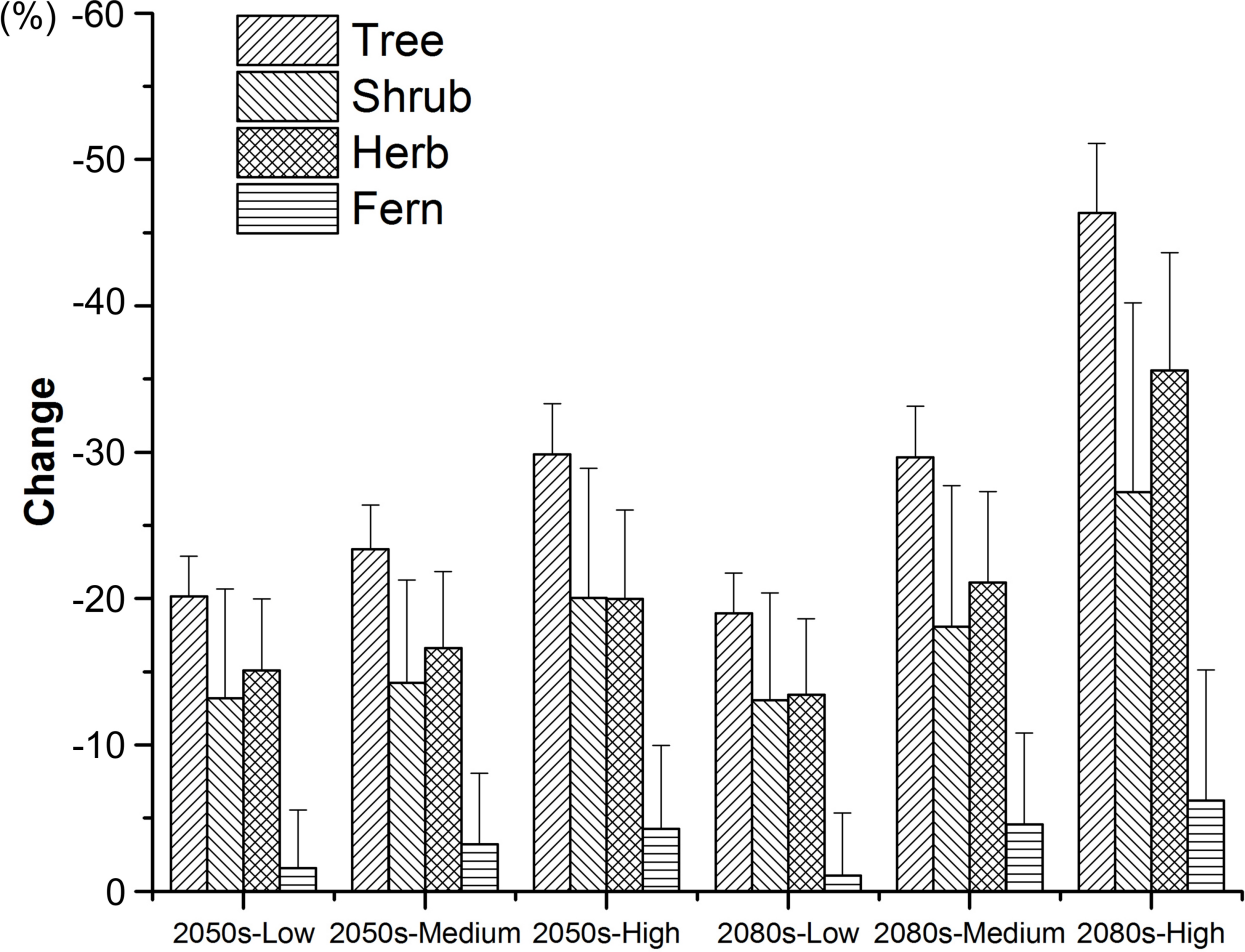
Changes in suitable climate for each threatened plant species in all the nature reserves according to plant type groups under the low, medium, and high greenhouse gas concentration scenarios for both the 2050s and 2080s. Standard errors are represented by error bars.

**Figure 4:**
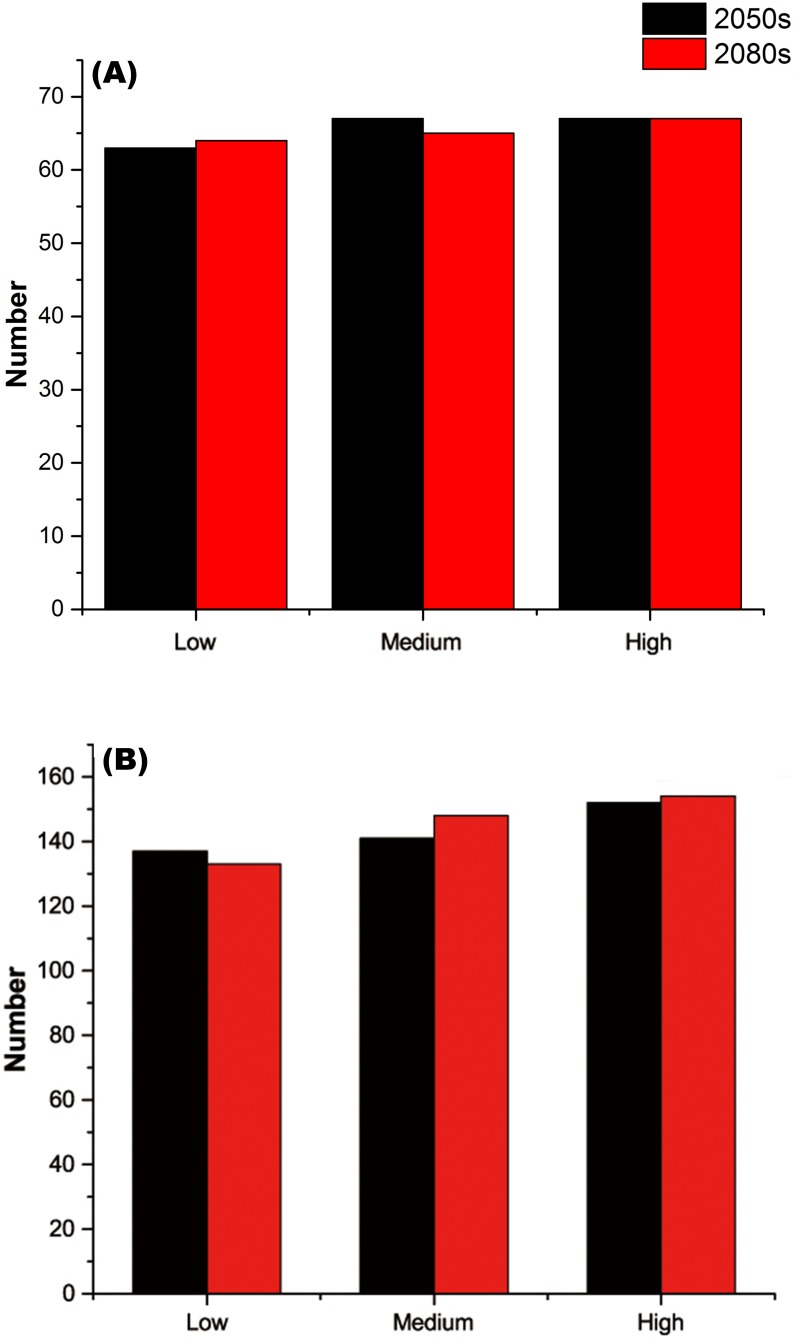
(A) Numbers of threatened plant species within all the nature reserves and (B) numbers of nature reserves with decreasing habitat suitability under the low, medium, and high greenhouse gas concentration scenarios for both the 2050s and 2080s.

For each threatened plant species across all nature reserves, there were significantly positive relationships between *C* (the change in climate suitability between current and future conditions) and *D* (the overlap between current and future climate suitability) under the low, medium, and high greenhouse gas concentration scenarios (*P* < 0.001; Fig. 1). For all threatened plant species belonging to each nature reserve with a decreasing *C* value, *D* values also decreased significantly (*P* < 0.001; Fig. 2). Thus, we focused on *C* because of these significantly positive relationships between *C* and *D* under the low and high greenhouse gas concentration scenarios (Figs. 1 and 2). Climate suitability is projected to decrease significantly from low to high concentration scenarios across the different plant type groups across all the nature reserves (*P* < 0.001; Fig. 3) and across all threatened plant species occurring within each nature reserve independently (*P* < 0.001). Furthermore, *C* values were projected to be larger in the 2080s than the 2050s in the medium and high concentration scenarios based plant type groups (*P* < 0.001; Fig. 3). Moreover, *C* increases significantly from low to high concentration scenarios (*P* < 0.001; Fig. 3). Habitat suitability for tree species would decrease most severely, and climate change may have the smallest impact on fern species across all the concentration scenarios (Fig. 3). The climate suitability of 63, 65, and 65 threatened plant species are projected to decrease in the low, medium, and high concentration scenarios, respectively, by both the 2050s and 2080s (Fig. 4A; Table S5). *Thuja koraiensis* is projected to have the largest decrease in climatically suitable habitat under the high concentration scenario by the 2080s (Table S5).

**Figure 5:**
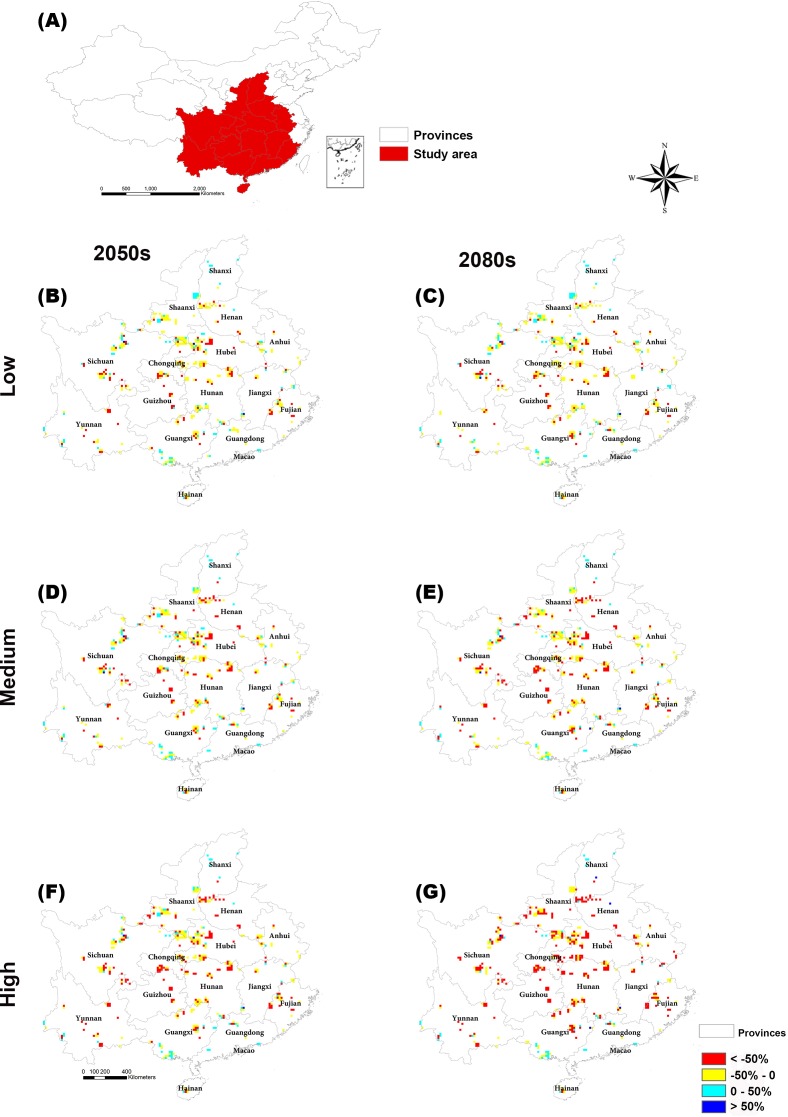
Distributions of suitable climate change for threatened plant species in nature reserves of central and southern China in the (B and C) low, (D and E) medium, and (F and G) high greenhouse gas concentration scenarios for both the 2050s and 2080s.

The regions with large changes in climate suitability during the current time period are distributed across central and southern China (Fig. S2). With increasing greenhouse gas concentrations, the habitat suitability for threatened plant species in nature reserves will decrease gradually (Figs. 4B and 5). The climate suitability of 132, 140, and 151 nature reserves are projected to decrease under the low, medium and high concentration scenarios, respectively, by both the 2050s and 2080s (Fig. 4B; Table S6). Furthermore, the number of nature reserves exhibiting decreased habitat suitability for threatened plant species was larger under the medium and high concentration scenarios for the 2050s relative to the 2080s (Figs. 4B and 5). We focused on the habitat suitability of threatened plant species in nature reserves under the high concentration scenario. The nature reserves with decreasing habitat suitability of threatened plant species were distributed across Henan, Shaanxi, Sichuan, Chongqing, Guizhou, Yunnan, Guangxi, Fujian, Jiangxi, and Anhui provinces (Fig. 5). Wudaoxia nature reserve (Hubei province) exhibited the largest decrease in climate suitability under the low concentration scenario (in the 2050s), the medium concentration scenario (in the 2080s), and the high concentration scenario (in the 2080s; Table S6).

## Discussion

We evaluated the climate suitability of threatened plant species in Chinese nature reserves under future climate change scenarios using occurrence locality data. We project that the habitat suitability of more than 60 threatened plants within more than 130 nature reserves would decrease under these projected climate change scenarios. Overall, this indicates that climate change may threaten habitat suitability of threatened plant species within Chinese nature reserves.

Annual mean temperature is projected to affect the habitat suitability of threatened tree, herb, and fern species most, while precipitation seasonality is the driving factor in changing habitat suitability for threatened shrub species. This indicates the importance of monitoring threatened plant species according to factors such as plant type. This is consistent with previous studies that found that annual mean temperature was the most important bioclimatic variable for the distribution and growth of trees, herbs, and ferns (Zhang et al., 2014; Yu et al., 2014; Wang et al., 2015). The annual mean temperature is projected to increase dramatically in the 2080s. Hence, annual mean temperature may dramatically alter the distribution of plant species. Dilts et al. (2015) showed that the water balance influenced by precipitation seasonality is related to the geographic distribution of most shrub species. By the 2080s, precipitation seasonality may also change substantially with increasing greenhouse gas concentrations. Hence, we also focused on the role of precipitation seasonality on habitat suitability or threatened plant species. Moreover, the impact of soil pH on habitat suitability for tree, shrub, and fern species was substantial (Ervin & Holly, 2011; Marschner, Crowley & Yang, 2004). Soil pH affects nutrient availability, which dramatically impacts habitat suitability (Ervin & Holly, 2011; Marschner, Crowley & Yang, 2004). To address the practical conservation issues, we must consider the impact of future climate change coupled with factors such as soil pH on habitat suitability for threatened plant species, particularly, tree, shrub, and fern species.

Based on the Global Strategy for Plant Conservation (http://www.cbd.int/gspc/), at least 75% of known threatened plant species are protected. Projected climate changes caused by high greenhouse gas emissions are projected to damage suitable habitats for plant species within Chinese nature reserves. The large shift in potential habitat distributions and decreases in habitats with suitable climates could leave potentially viable populations of threatened plant species vulnerable to extinction (Fordham et al., 2013; Costion et al., 2015; Van Andel et al., 2015). Hence, we compiled a list of important plants for conservation within China including more than 60 threatened plant species (over 73.2% of all 82 species), for example, *T. koraiensis*, which is particularly endangered by trends of climate suitability under the high concentration scenario. In particular, extreme climate events and rapid changes in climate can cause physiological stress and damage to plants (Bastos et al., 2014; Zinta et al., 2014). Threatened plant species are already in danger and thus are vulnerable to extreme climate events like the 2003 summer heatwave, showing that inappropriate land management can threaten the existence of plant species (Bastos et al., 2014; Zinta et al., 2014; Wujeska-Klause, Bossinger & Tausz, 2015). Furthermore, we found that the threatened tree species within nature reserves would be strongly affected by climate change, particularly under the high concentration scenario by the 2080s. The distributions of suitable habitats for tree species may shift as a consequence of climate change. Alberto et al. (2013) has shown that evolutionary responses are required for tree populations to track climate change. Hence, we must assess the impact of climate change on habitat suitability for tree species when managing the conservation of threatened plant species. Although fern species may be affected less by climate change, we still must pay attention to the response of fern species like *Alsophila denticulate*, *Cibotium barometz*, and *Alsophila metteniana* because their suitable habitats decrease substantially under the high concentration scenario. Hence, we must monitor the changing dynamics of potential distributions of threatened plants under climate change and prevent habitat degeneration in order to stabilize plant populations (Thuiller et al., 2005; Keith et al., 2008; Araújo et al., 2011).

Furthermore, many threatened species are valued for their economic potential and medicinal properties (Wang et al., 2015). For example, the important anticancer drug camptothecin is extracted from *Camptotheca acuminata* (Kusari, Zühlke & Spiteller, 2009). However, the habitat of viable populations of *C. acuminata* has decreased as a result of environmental pollution, deforestation, and erosion (Yu et al., 2014; Wang et al., 2015). Moreover, climate change may aggravate the already stressed remnant populations of *C. acuminata* (Table S1). The value of wild plant resources may be diminished by climate change. Previous studies have also shown that plant species may need to escape to higher latitudes and altitudes to evade rising temperatures (Thuiller et al., 2011). Furthermore, threatened plant species with narrow climate niches would be threatened severely by climate change (Ma et al., 2013). Our results, in combination with those of previous studies, highlight the need for monitoring and managing threatened species under projected decreasing climate suitability as well as the value of determining congruence between current and future climatically suitable habitats (Thuiller et al., 2011; Fiedler, 2012; Costion et al., 2015).

Climate change threatens habitat suitability for threatened plant species in more than 130 nature reserves (77.4% of all the nature reserves in the analysis) under the low greenhouse gas concentration scenario, 140 reserves under the medium concentration scenario (83.3%), and 165 reserves (98.2%) under the high concentration scenario by both the 2050s and 2080s. This indicates that climate change will likely decrease the capacity of these nature reserves to protect threatened plants. These nature reserves play an important role in ecosystem services (Xu & Melick, 2007; Araújo et al., 2011; Yu et al., 2014). For example, Ailaoshan nature reserve exhibits rich plant diversity and stores a large quantity of carbon (Qiao et al., 2014). However, climate change will alter the habitat suitability for many threatened plant species in this nature reserve, possibly disrupting ecosystem services such as carbon storage (Heller & Zavaleta, 2009). Hence, we must take effective measures to reduce the negative effect of climate change on threatened plants within nature reserves, particularly Wudaoxia nature reserve as it is projected to suffer most severely in term of decreasing habitat suitability for threatened plant species.

## Conclusions

Our method serves as an important reference for the conservation of plant diversity in the face of climate change. This goal will require both increased research and a continually developed capacity to forecast future climate conditions, as well as identification of the responses of threatened plant species to climate change. An integrative assessment of climate suitability using occurrence localities will enhance the conservation status system for threatened plant species. As climatically suitable habitats decrease for threatened plant species, niche gaps may increase in the future. Climate change may threaten habitat suitability for more than 60 threatened plant species within Chinese nature reserves across more than 130 nature reserves. Hence, climate change is likely to threaten habitat suitability for threatened plant species throughout Chinese nature reserves. Future studies should consider more local scales when making assessments of conservation status for threatened plant species. We urgently need innovative evaluation approaches for threatened plant species at all scales.

##  Supplemental Information

10.7717/peerj.2091/supp-1 Table S1 Study species with the numbers of occurrence localities (Record column) and AUC values

10.7717/peerj.2091/supp-2 Table S2 Environmental variables and loadings on the first two principal components (PC1 and PC2) based on a principal component analysis. Bold text indicates the variables used as input for Maxent modelling

10.7717/peerj.2091/supp-3 Table S3 Pearson's correlation coefficient between the environmental variables considered for analysis. Environmental variable codes (i.e., the first column) are given in the first column of Table S2

10.7717/peerj.2091/supp-4 Table S4 Permutation importance of predictor variables. Environmental variables codes (i.e., the column headers) are given in the first column of Table S2

10.7717/peerj.2091/supp-5 Table S5 Summary of changes in climate suitability for threatened plant species

10.7717/peerj.2091/supp-6 Table S6 Summary of changes in climate suitability for threatened plant species according to nature reserve

10.7717/peerj.2091/supp-7 Table S7 Summary of overlap between current and future climatically suitable habitat for threatened plant species

10.7717/peerj.2091/supp-8 Table S8 Summary of overlap between current and future climatically suitable habitats for threatened plant species organized by nature reserve

10.7717/peerj.2091/supp-9 Figure S1 Relationships between the average PI values and the affected proportion of the total plant species based on PC results for (A) tree, (B) shrub, (C) herb, and (D) fern species

10.7717/peerj.2091/supp-10 Figure S2 Distributions of habitats exhibiting suitable climate change for threatened plant species in Chinese nature reserves under (B and C) low, (D and E) medium, and (F and G) high greenhouse gas concentration scenarios for both the 2050s and 2080s (respectively)

10.7717/peerj.2091/supp-11 Data S1 References for raw data sources

## Additional Information and Declarations

### Data Availability

The following information was supplied regarding data availability: The raw data came from scientific research reports of nature reserves that were published or assessed by the Chinese government in China. The reference list can be found in Data S1.

## References

[ref-1] Alberto FJ, Aitken SN, Alía R, González-Martínez SC, Hänninen H, Kremer A, Lefèvre F, Lenormand T, Yeaman S, Whetten R, Savolainen O (2013). Potential for evolutionary responses to climate change---evidence from tree populations. Global Change Biology.

[ref-2] Aranda SC, Lobo JM (2011). How well does presence-only-based species distribution modelling predict assemblage diversity? A case study of the Tenerife flora. Ecography.

[ref-3] Araújo MB, Alagador D, Cabeza M, Nogués-Bravo D, Thuiller W (2011). Climate change threatens European conservation areas. Ecology Letters.

[ref-4] Araújo MB, Cabeza M, Thuiller W, Hannah L, Williams PH (2004). Would climate change drive species out of reserves? An assessment of existing reserve-selection methods. Global Change Biology.

[ref-5] Bastos A, Gouveia CM, Trigo RM, Running SW (2014). Analysing the spatio-temporal impacts of the 2003 and 2010 extreme heatwaves on plant productivity in Europe. Biogeosciences.

[ref-6] Bellard C, Bertelsmeier C, Leadley P, Thuiller W, Courchamp F (2012). Impacts of climate change on the future of biodiversity. Ecology Letters.

[ref-7] China's State Forestry Administration and the Institute of Botany, Chinese Academy of Sciences (2013). Rare and endangered plants in China.

[ref-8] Corlett RT, Westcott DA (2013). Will plant movements keep up with climate change?. Trends in Ecology and Evolution.

[ref-9] Costion CM, Simpson L, Pert PL, Carlsen MM, Kress WJ, Crayn D (2015). Will tropical mountaintop plant species survive climate change? Identifying key knowledge gaps using species distribution modelling in Australia. Biological Conservation.

[ref-10] Dilts TE, Weisberg PJ, Dencker CM, Chambers JC (2015). Functionally relevant climate variables for arid lands: a climatic water deficit approach for modelling desert shrub distributions. Journal of Biogeography.

[ref-11] Elith J, Phillips SJ, Hastie T, Dudík M, Chee YE, Yates CJ (2011). A statistical explanation of MaxEnt for ecologists. Diversity and Distributions.

[ref-12] Ervin GN, Holly DC (2011). Examining local transferability of predictive species distribution models for invasive plants: an example with cogongrass (*Imperata cylindrica*). Invasive Plant Science and Management.

[ref-13] Farashi A, Najafabadi MS (2015). Modeling the spread of invasive nutrias (Myocastor coypus) over Iran. Ecological Complexity.

[ref-14] Fiedler PL (2012). Conservation biology: the theory and practice of nature conservation preservation and management.

[ref-15] Fordham DA, Akçakaya HR, Araújo MB, Elith J, Keith DA, Pearson R, Auld TD, Mellin C, Morgan JW, Regan TJ, Tozer M, Watts MJ, White M, Wintle BA, Yates C, Brook BW (2012). Plant extinction risk under climate change: are forecast range shifts alone a good indicator of species vulnerability to global warming?. Global Change Biology.

[ref-16] Fordham DA, Akçakaya HR, Araújo MB, Keith DA, Brook BW (2013). Tools for integrating range change, extinction risk and climate change information into conservation management. Ecography.

[ref-17] Groom QJ (2013). Some poleward movement of British native vascular plants is occurring, but the fingerprint of climate change is not evident. PeerJ.

[ref-18] Groves C, Game E, Anderson M, Cross M, Enquist C, Ferdaña Z, Girvetz E, Gondor A, Hall K, Higgins J, Marshall R, Popper K, Schill S, Shafer S (2012). Incorporating climate change into systematic conservation planning. Biodiversity and Conservation.

[ref-19] Guisan A, Petitpierre B, Broennimann O, Daehler C, Kueffer C (2014). Unifying niche shift studies: insights from biological invasions. Trends in Ecology & Evolution.

[ref-20] Hansen AJ, Spies TA, Swanson FJ, Ohmann JL (1991). Conserving biodiversity in managed forests. BioScience.

[ref-21] Heller NE, Zavaleta ES (2009). Biodiversity management in the face of climate change: a review of 22 years of recommendations. Biological Conservation.

[ref-22] Hijmans RJ, Graham CH (2006). The ability of climate envelope models to predict the effect of climate change on species distributions. Global Change Biology.

[ref-23] Iverson LR, McKenzie D (2013). Tree-species range shifts in a changing climate: detecting, modeling, assisting. Landscape Ecology.

[ref-24] Keith DA, Akçakaya HR, Thuiller W, Midgley GF, Pearson RG, Phillips SJ, Regan HM, Araújo MB, Rebelo TG (2008). Predicting extinction risks under climate change: coupling stochastic population models with dynamic bioclimatic habitat models. Biology Letters.

[ref-25] Kusari S, Zühlke S, Spiteller M (2009). An endophytic fungus from *Camptotheca acuminata*that produces camptothecin and analogues. Journal of Natural Products.

[ref-26] Lawson CR, Bennie JJ, Thomas CD, Hodgson JA, Wilson RJ (2012). Local and landscape management of an expanding range margin under climate change. Journal of Applied Ecology.

[ref-27] Liu J, Diamond J (2005). China's environment in a globalizing world. Nature.

[ref-28] Ma Y, Chen G, Grumbine RE, Dao Z, Sun W, Guo H (2013). Conserving plant species with extremely small populations (PSESP) in China. Biodiversity and Conservation.

[ref-29] Marschner P, Crowley D, Yang CH (2004). Development of specific rhizosphere bacterial communities in relation to plant species, nutrition and soil type. Plant and Soil.

[ref-30] McConkey KR, Prasad S, Corlett RT, Campos-Arceiz A, Brodie JF, Rogers H, Santamaria L (2012). Seed dispersal in changing landscapes. Biological Conservation.

[ref-31] Merow C, Smith MJ, Silander JA (2013). A practical guide to MaxEnt for modeling species' distributions: what it does, and why inputs and settings matter. Ecography.

[ref-32] Oke OA, Thompson KA (2015). Distribution models for mountain plant species: the value of elevation. Ecological Modelling.

[ref-33] Pearson RG, Raxworthy CJ, Nakamura M, Townsend Peterson A (2007). Predicting species distributions from small numbers of occurrence records: a test case using cryptic geckos in Madagascar. Journal of Biogeography.

[ref-34] Pineda E, Lobo JM (2012). The performance of range maps and species distribution models representing the geographic variation of species richness at different resolutions. Global Ecology and Biogeography.

[ref-35] Qiao NA, Schaefer D, Blagodatskaya E, Zou X, Xu X, Kuzyakov Y (2014). Labile carbon retention compensates for CO2 released by priming in forest soils. Global Change Biology.

[ref-36] Ren H, Shen WJ, Lu HF, Wen XY, Jian SG (2007). Degraded ecosystems in China: status, causes, and restoration efforts. Landscape and Ecological Engineering.

[ref-37] Rödder D, Engler JO (2011). Quantitative metrics of overlaps in Grinnellian niches: advances and possible drawbacks. Global Ecology and Biogeography.

[ref-38] Saetersdal M, Line JM, Birks HJB (1993). How to maximize biological diversity in nature reserve selection: vascular plants and breeding birds in deciduous woodlands, western Norway. Biological Conservation.

[ref-39] Summers DM, Bryan BA, Crossman ND, Meyer WS (2012). Species vulnerability to climate change: impacts on spatial conservation priorities and species representation. Global Change Biology.

[ref-40] Thuiller W, Lavergne S, Roquet C, Boulangeat I, Lafourcade B, Araujo MB (2011). Consequences of climate change on the tree of life in Europe. Nature.

[ref-41] Thuiller W, Lavorel S, Araújo MB, Sykes MT, Prentice IC (2005). Climate change threats to plant diversity in Europe. Proceedings of the National Academy of Sciences of the United States of America.

[ref-42] Tingley R, Vallinoto M, Sequeira F, Kearney MR (2014). Realized niche shift during a global biological invasion. Proceedings of the National Academy of Sciences of the United States of America.

[ref-43] Van Andel TR, Croft S, Van Loon EE, Quiroz D, Towns AM, Raes N (2015). Prioritizing West African medicinal plants for conservation and sustainable extraction studies based on market surveys and species distribution models. Biological Conservation.

[ref-44] Walsh M, Haseeb MA (2015). Modeling the ecologic niche of plague in sylvan and domestic animal hosts to delineate sources of human exposure in the western United States. PeerJ.

[ref-45] Wan J, Wang C, Yu J, Nie S, Han S, Zu Y, Chen C, Liu J, Wang Q (2014). Model-based conservation planning of the genetic diversity of *Phellodendron amurense* Rupr due to climate change. Ecology and Evolution.

[ref-46] Wang CJ, Wan JZ, Mu XY, Zhang ZX (2015). Management planning for endangered plant species in priority protected areas. Biodiversity and Conservation.

[ref-47] Warren DL, Glor RE, Turelli M (2008). Environmental niche equivalency versus conservatism: quantitative approaches to niche evolution. Evolution.

[ref-48] Warren DL, Glor RE, Turelli M (2010). ENMTools: a toolbox for comparative studies of environmental niche models. Ecography.

[ref-49] Wu R, Zhang S, Yu DW, Zhao P, Li X, Wang L, Wang L, Qian Y, Long Y (2011). Effectiveness of China's nature reserves in representing ecological diversity. Frontiers in Ecology and the Environment.

[ref-50] Wujeska-Klause A, Bossinger G, Tausz M (2015). Responses to heatwaves of gas exchange, chlorophyll fluorescence and antioxidants ascorbic acid and glutathione in congeneric pairs of Acacia and Eucalyptus species from relatively cooler and warmer climates. Trees.

[ref-51] Xu J, Melick DR (2007). Rethinking the effectiveness of public protected areas in southwestern China. Conservation Biology.

[ref-52] Yang W, Ma K, Kreft H (2014). Environmental and socio-economic factors shaping the geography of floristic collections in China. Global Ecology and Biogeography.

[ref-53] Yu J, Wang C, Wan J, Han S, Wang Q, Nie S (2014). A model-based method to evaluate the ability of nature reserves to protect endangered tree species in the context of climate change. Forest Ecology and Management.

[ref-54] Zhang Z, He JS, Li J, Tang Z (2015). Distribution and conservation of threatened plants in China. Biological Conservation.

[ref-55] Zhang MG, Zhou ZK, Chen WY, Cannon CH, Raes N, Slik JW (2014). Major declines of woody plant species ranges under climate change in Yunnan, China. Diversity and Distributions.

[ref-56] Zinta G, AbdElgawad H, Domagalska MA, Vergauwen L, Knapen D, Nijs I, Janssens IA, Beemster GTS, Asard H (2014). Physiological, biochemical, and genome-wide transcriptional analysis reveals that elevated CO_2_ mitigates the impact of combined heat wave and drought stress in Arabidopsis thaliana at multiple organizational levels. Global Change Biology.

