# Supplementary material for: Climate change may threaten habitat suitability of threatened plant species within Chinese nature reserves
Source: PeerJ. 2016 Jun 14;4:e2091. doi: 10.7717/peerj.2091 (PMC4911960; doi:10.7717/peerj.2091)
Supplement: Table S1 [file peerj-04-2091-s001.docx]

**Table S1.** Study species with the numbers of occurrence localities (Record column) and AUC values.

| Name | Family | Type | Record | Test AUC | Training AUC |
| --- | --- | --- | --- | --- | --- |
| *Magnolia officinalis* subsp. *biloba* | Magnoliaceae | Tree | 95 | 0.962 | 0.975 |
| *Torreya fargesii* | Taxaceae | Tree | 48 | 0.977 | 0.987 |
| *Pseudotaxus chienii* | Taxaceae | Shrub | 12 | 0.944 | 0.968 |
| *Semiliquidambar cathayensis* | Hamamelidaceae | Tree | 33 | 0.977 | 0.989 |
| *Cephalotaxus oliveri* | Cephalotaxaceae | Shrub | 69 | 0.971 | 0.980 |
| *Bretschneidera sinensis* | Bretschneideraceae | Tree | 117 | 0.966 | 0.981 |
| *Thuja koraiensis* | Cupressaceae | Tree | 15 | 0.996 | 0.998 |
| *Phellodendron chinense* | Rutaceae | Tree | 77 | 0.976 | 0.984 |
| *Brasenia schreberi* | Nymphaeaceae | Herb | 13 | 0.932 | 0.954 |
| *Alsophila denticulata* | Cyatheaceae | Fern | 30 | 0.972 | 0.986 |
| *Picea neoveitchii* | Pinaceae | Tree | 48 | 0.989 | 0.992 |
| *Alsophila gigantea* | Cyatheaceae | Fern | 14 | 0.970 | 0.984 |
| *Taxus cuspidata* | Taxaceae | Tree | 40 | 0.967 | 0.992 |
| *Caryota obtusa* | Palmae | Tree | 15 | 0.974 | 0.986 |
| *Kingdonia uniflora* | Ranunculaceae | Herb | 11 | 0.889 | 0.938 |
| *Michelia wilsonii* | Magnoliaceae | Tree | 17 | 0.993 | 0.995 |
| *Liriodendron chinense* | Magnoliaceae | Tree | 67 | 0.942 | 0.965 |
| *Torreya grandis* | Taxaceae | Tree | 42 | 0.970 | 0.982 |
| *Sagittaria natans* | Alismataceae | Herb | 38 | 0.977 | 0.986 |
| *Fokienia hodginsii* | Cupressaceae | Tree | 40 | 0.967 | 0.982 |
| *Davidia involucrata* | Nyssaceae | Tree | 92 | 0.979 | 0.987 |
| *Davidia involucrata* var. *vilmoriniana* | Nyssaceae | Tree | 46 | 0.988 | 0.992 |
| *Gmelina hainanensis* | Verbenaceae | Tree | 11 | 0.973 | 0.982 |
| *Alsophila podophylla* | Cyatheaceae | Fern | 29 | 0.973 | 0.987 |
| *Toona ciliata* | Meliaceae | Tree | 60 | 0.930 | 0.956 |
| *Taxus wallichiana* var. *chinensis* | Taxaceae | Tree | 156 | 0.966 | 0.979 |
| *Ormosia hosiei* | Leguminosae | Tree | 44 | 0.956 | 0.976 |
| *Meconopsis punicea* | Papaveraceae | Herb | 22 | 0.982 | 0.989 |
| *Pinus koraiensis* | Pinaceae | Tree | 95 | 0.971 | 0.980 |
| *Magnolia officinalis* | Magnoliaceae | Tree | 111 | 0.962 | 0.979 |
| *Ormosia henryi* | Leguminosae | Tree | 103 | 0.962 | 0.976 |
| *Pinus kwangtungensis* | Pinaceae | Tree | 30 | 0.967 | 0.985 |
| *Castanopsis concinna* | Fagaceae | Tree | 10 | 0.975 | 0.983 |
| *Phellodendron amurense* | Rutaceae | Tree | 159 | 0.936 | 0.965 |
| *Pseudotsuga sinensis* | Pinaceae | Tree | 32 | 0.982 | 0.991 |
| *Cibotium barometz* | Dicksoniaceae | Fern | 80 | 0.945 | 0.963 |
| *Pseudolarix amabilis* | Pinaceae | Tree | 18 | 0.976 | 0.991 |
| *Fagopyrum dibotrys* | Polygonaceae | Herb | 118 | 0.938 | 0.962 |
| *Zelkova schneideriana* | Ulmaceae | Tree | 111 | 0.954 | 0.970 |
| *Cercidiphyllum japonicum* | Cercidiphyllaceae | Tree | 152 | 0.968 | 0.979 |
| *Nelumbo nucifera* | Nymphaeaceae | Herb | 84 | 0.902 | 0.939 |
| *Rhoiptelea chiliantha* | Rhoipteleaceae | Tree | 11 | 0.943 | 0.963 |
| *Toona ciliata* var. *pubescens* | Meliaceae | Tree | 27 | 0.937 | 0.967 |
| *Phoebe bournei* | Lauraceae | Tree | 81 | 0.967 | 0.977 |
| *Aldrovanda vesiculosa* | Droseraceae | Herb | 30 | 0.985 | 0.994 |
| *Taxus wallichiana* var. *mairei* | Taxaceae | Tree | 153 | 0.942 | 0.966 |
| *Phoebe zhennan* | Lauraceae | Tree | 67 | 0.979 | 0.984 |
| *Abies chensiensis* | Pinaceae | Tree | 41 | 0.969 | 0.984 |
| *Zenia insignis* | Leguminosae | Tree | 42 | 0.967 | 0.981 |
| *Machilus nanmu* | Lauraceae | Tree | 16 | 0.923 | 0.956 |
| *Eurycorymbus cavaleriei* | Sapindaceae | Tree | 60 | 0.974 | 0.982 |
| *Euchresta japonica* | Leguminosae | Shrub | 16 | 0.943 | 0.964 |
| *Anisodus tanguticus* | Solanaceae | Herb | 15 | 0.994 | 0.997 |
| *Dipentodon sinicus* | Celastraceae | Shrub | 10 | 0.889 | 0.935 |
| *Ceratopteris thalictroides* | Parkeriaceae | Fern | 17 | 0.927 | 0.951 |
| [*Tetracentron sinense*](http://foc.eflora.cn/content.aspx?TaxonId=200008490) | Magnoliaceae | Tree | 183 | 0.956 | 0.975 |
| *Fraxinus mandschurica* | Oleaceae | Tree | 117 | 0.954 | 0.973 |
| [*Metasequoia glyptostroboides*](http://foc.eflora.cn/content.aspx?TaxonId=200005396) | Taxodiaceae | Tree | 61 | 0.916 | 0.945 |
| *Larix mastersiana* | Pinaceae | Tree | 10 | 0.966 | 0.974 |
| *Brainea insignis* | Blechnaceae | Fern | 15 | 0.923 | 0.946 |
| *Malania oleifera* | Olacaceae | Tree | 11 | 0.998 | 1.000 |
| *Alsophila spinulosa* | Cyatheaceae | Fern | 34 | 0.950 | 0.972 |
| *Taiwania cryptomerioides* | Taxodiaceae | Tree | 10 | 0.968 | 0.983 |
| *Cinnamomum japonicum* | Lauraceae | Tree | 14 | 0.878 | 0.927 |
| *Myriophyllum ussuriense* | Haloragidaceae | Herb | 44 | 0.953 | 0.976 |
| *Oyama wilsonii* | Magnoliaceae | Shrub | 18 | 0.923 | 0.943 |
| *Camptotheca acuminata* | Nyssaceae | Tree | 118 | 0.953 | 0.972 |
| *Emmenopterys henryi* | Rubiaceae | Tree | 182 | 0.958 | 0.971 |
| *Alsophila metteniana* | Cyatheaceae | Fern | 21 | 0.968 | 0.985 |
| *Triaenophora rupestris* | Scrophulariaceae | Herb | 17 | 0.965 | 0.981 |
| *Glycine soja* | Leguminosae | Herb | 419 | 0.879 | 0.907 |
| *Ginkgo biloba* | Ginkgoaceae | Tree | 148 | 0.932 | 0.959 |
| *Picea brachytyla* var. *complanata* | Pinaceae | Tree | 22 | 0.974 | 0.991 |
| *Cinnamomum longepaniculatum* | Lauraceae | Tree | 27 | 0.975 | 0.986 |
| *Cinnamomum camphora* | Lauraceae | Tree | 166 | 0.944 | 0.961 |
| *Phoebe chekiangensis* | Lauraceae | Tree | 14 | 0.936 | 0.983 |
| *Zoysia sinica* | Gramineae | Herb | 42 | 0.940 | 0.965 |
| *Platycrater arguta* | Saxifragaceae | Shrub | 10 | 0.982 | 0.986 |
| *Acer amplum* subsp. *catalpifolium* | Aceraceae | Tree | 20 | 0.971 | 0.985 |
| *Tilia amurensis* | Tiliaceae | Tree | 148 | 0.960 | 0.976 |
| *Madhuca pasquieri* | Sapotaceae | Tree | 14 | 0.967 | 0.990 |
| *Chosenia arbutifolia* | Salicaceae | Tree | 107 | 0.975 | 0.983 |
