# Supplementary material for: Climate change may threaten habitat suitability of threatened plant species within Chinese nature reserves
Source: PeerJ. 2016 Jun 14;4:e2091. doi: 10.7717/peerj.2091 (PMC4911960; doi:10.7717/peerj.2091)
Supplement: Table S2 [file peerj-04-2091-s002.docx]

**Table S2.** Environmental variables and loadings on the first two principal components (PC1 and PC2) based on a principal component analysis. Bold text indicates the variables used as input for Maxent modelling.

| Code | Variables | Type | Source | PC1 | PC2 |
| --- | --- | --- | --- | --- | --- |
| **BLD** | **Bulk Density** | **Soil** | [**www.soilgrids.org**](http://www.soilgrids.org/) | **-0.129** | **-0.101** |
| **CEC** | **Cation Exchange Capacity** | **Soil** | [**www.soilgrids.org**](http://www.soilgrids.org/) | **-0.182** | **0.076** |
| **CLYPPT** | **Soil Texture Fraction Clay** | **Soil** | [**www.soilgrids.org**](http://www.soilgrids.org/) | **0.064** | **-0.149** |
| **CRFVOL** | **Coarse Fragments Volumetric** | **Soil** | [**www.soilgrids.org**](http://www.soilgrids.org/) | **-0.039** | **0.016** |
| **OCSTHA** | **Soil Organic Carbon Stock** | **Soil** | [**www.soilgrids.org**](http://www.soilgrids.org/) | **-0.158** | **0.069** |
| ORCDRC | Soil Organic Carbon Content | Soil | [www.soilgrids.org](http://www.soilgrids.org/) | -0.139 | 0.068 |
| **PHIHOX** | **Soil PH** | **Soil** | [**www.soilgrids.org**](http://www.soilgrids.org/) | **-0.201** | **-0.001** |
| **SLTPPT** | **Soil Texture Fraction Silt** | **Soil** | [**www.soilgrids.org**](http://www.soilgrids.org/) | **-0.232** | **0.049** |
| **SNDPPT** | **Soil Texture Fraction Sand** | **Soil** | [**www.soilgrids.org**](http://www.soilgrids.org/) | **0.028** | **-0.115** |
| Alt | Altitude | Topography | www.worldclim.org | 0.175 | 0.253 |
| **Aspect** | **Aspect** | **Topography** | www.worldclim.org | **-0.018** | **-0.024** |
| **Slope** | **Slope** | **Topography** | www.worldclim.org | **-0.008** | **0.003** |
| **Globcover** | **Land Use and Land Cover** | **Degree of Naturalness** | due.esrin.esa.int | **0.014** | **0.094** |
| **Bio1** | **Annual Mean Temperature** | **Climate** | www.worldclim.org | **0.203** | **0.251** |
| **Bio2** | **Mean Diurnal Range** | **Climate** | www.worldclim.org | **-0.119** | **0.240** |
| **Bio3** | **Isothermality** | **Climate** | www.worldclim.org | **0.205** | **0.151** |
| **Bio4** | **Temperature Seasonality** | **Climate** | www.worldclim.org | **-0.240** | **0.109** |
| Bio5 | Max Temperature of Warmest Month | Climate | www.worldclim.org | 0.197 | 0.256 |
| Bio6 | Min Temperature of Coldest Month | Climate | www.worldclim.org | 0.210 | 0.245 |
| Bio7 | Temperature Annual Range (BIO5-BIO6) | Climate | www.worldclim.org | -0.250 | 0.112 |
| Bio8 | Mean Temperature of Wettest Quarter | Climate | www.worldclim.org | 0.196 | 0.256 |
| Bio9 | Mean Temperature of Driest Quarter | Climate | www.worldclim.org | 0.209 | 0.246 |
| Bio10 | Mean Temperature of Warmest Quarter | Climate | www.worldclim.org | 0.198 | 0.255 |
| Bio11 | Mean Temperature of Coldest Quarter | Climate | www.worldclim.org | 0.208 | 0.247 |
| **Bio12** | **Annual Precipitation** | **Climate** | www.worldclim.org | **0.214** | **-0.231** |
| Bio13 | Precipitation of Wettest Month | Climate | www.worldclim.org | 0.184 | -0.218 |
| Bio14 | Precipitation of Driest Month | Climate | www.worldclim.org | 0.201 | -0.202 |
| **Bio15** | **Precipitation Seasonality** | **Climate** | www.worldclim.org | **-0.221** | **0.105** |
| Bio16 | Precipitation of Wettest Quarter | Climate | www.worldclim.org | 0.191 | -0.219 |
| Bio17 | Precipitation of Driest Quarter | Climate | www.worldclim.org | 0.199 | -0.200 |
| Bio18 | Precipitation of Warmest Quarter | Climate | www.worldclim.org | 0.158 | -0.194 |
| Bio19 | Precipitation of Coldest Quarter | Climate | www.worldclim.org | 0.198 | -0.197 |
