# Supplementary material for: Climate change may threaten habitat suitability of threatened plant species within Chinese nature reserves
Source: PeerJ. 2016 Jun 14;4:e2091. doi: 10.7717/peerj.2091 (PMC4911960; doi:10.7717/peerj.2091)
Supplement: Table S3 [file peerj-04-2091-s003.docx]

**Table S3.** Pearson's correlation coefficient between the environmental variables considered for analysis. Environmental variable codes (i.e., the first column) are given in the first column of Table S2.

| Variables | SNDPPT | SLTPPT | PHIHOX | ORCDRC | OCSTHA | Globcover | CRFVOL | CLYPPT | CEC | BLD | Aspect | Alt | Slope | Bio1 | Bio2 | Bio3 |
| --- | --- | --- | --- | --- | --- | --- | --- | --- | --- | --- | --- | --- | --- | --- | --- | --- |
| SNDPPT | 1 | -0.324 | 0.033 | 0.228 | 0.116 | -0.089 | 0.203 | -0.122 | -0.333 | 0.260 | -0.029 | -0.131 | 0.150 | -0.216 | -0.205 | 0.029 |
| SLTPPT | -0.324 | 1 | 0.628 | 0.331 | 0.446 | -0.074 | 0.100 | -0.061 | 0.612 | 0.597 | 0.073 | -0.247 | -0.069 | -0.257 | 0.395 | -0.422 |
| PHIHOX | 0.033 | 0.628 | 1 | 0.049 | 0.061 | -0.314 | 0.126 | 0.278 | 0.547 | 0.614 | 0.012 | -0.253 | 0.119 | -0.334 | 0.134 | -0.308 |
| ORCDRC | 0.228 | 0.331 | 0.049 | 1 | 0.910 | 0.216 | 0.394 | -0.443 | 0.394 | 0.076 | 0.018 | 0.039 | 0.092 | -0.109 | 0.341 | -0.137 |
| OCSTHA | 0.116 | 0.446 | 0.061 | 0.910 | 1 | 0.228 | 0.080 | -0.420 | 0.351 | 0.178 | 0.011 | -0.073 | -0.097 | -0.119 | 0.439 | -0.243 |
| Globcover | -0.089 | -0.074 | -0.314 | 0.216 | 0.228 | 1 | 0.043 | -0.357 | 0.039 | -0.275 | 0.009 | 0.176 | -0.076 | 0.205 | 0.207 | 0.107 |
| CRFVOL | 0.203 | 0.100 | 0.126 | 0.394 | 0.080 | 0.043 | 1 | -0.177 | 0.327 | 0.156 | 0.053 | 0.159 | 0.458 | -0.069 | -0.062 | 0.142 |
| CLYPPT | -0.122 | -0.061 | 0.278 | -0.443 | -0.420 | -0.357 | -0.177 | 1 | 0.131 | 0.233 | -0.053 | -0.175 | 0.079 | -0.186 | -0.533 | -0.005 |
| CEC | -0.333 | 0.612 | 0.547 | 0.394 | 0.351 | 0.039 | 0.327 | 0.131 | 1 | 0.189 | 0.020 | 0.007 | 0.227 | -0.158 | 0.186 | -0.211 |
| BLD | 0.260 | 0.597 | 0.614 | 0.076 | 0.178 | -0.275 | 0.156 | 0.233 | 0.189 | 1 | 0.024 | -0.411 | -0.018 | -0.386 | -0.024 | -0.297 |
| Aspect | -0.029 | 0.073 | 0.012 | 0.018 | 0.011 | 0.009 | 0.053 | -0.053 | 0.020 | 0.024 | 1 | -0.082 | -0.052 | -0.073 | -0.052 | -0.115 |
| Alt | -0.131 | -0.247 | -0.253 | 0.039 | -0.073 | 0.176 | 0.159 | -0.175 | 0.007 | -0.411 | -0.082 | 1 | 0.158 | 0.918 | 0.297 | 0.752 |
| Slope | 0.150 | -0.069 | 0.119 | 0.092 | -0.097 | -0.076 | 0.458 | 0.079 | 0.227 | -0.018 | -0.052 | 0.158 | 1 | -0.053 | -0.135 | 0.153 |
| Bio1 | -0.216 | -0.257 | -0.334 | -0.109 | -0.119 | 0.205 | -0.069 | -0.186 | -0.158 | -0.386 | -0.073 | 0.918 | -0.053 | 1 | 0.324 | 0.707 |
| Bio2 | -0.205 | 0.395 | 0.134 | 0.341 | 0.439 | 0.207 | -0.062 | -0.533 | 0.186 | -0.024 | -0.052 | 0.297 | -0.135 | 0.324 | 1 | 0.256 |
| Bio3 | 0.029 | -0.422 | -0.308 | -0.137 | -0.243 | 0.107 | 0.142 | -0.005 | -0.211 | -0.297 | -0.115 | 0.752 | 0.153 | 0.707 | 0.256 | 1 |
| Bio4 | -0.260 | 0.669 | 0.300 | 0.361 | 0.555 | 0.135 | -0.220 | -0.464 | 0.293 | 0.192 | 0.057 | -0.274 | -0.318 | -0.169 | 0.658 | -0.527 |
| Bio5 | -0.224 | -0.239 | -0.327 | -0.099 | -0.103 | 0.210 | -0.074 | -0.200 | -0.150 | -0.382 | -0.072 | 0.915 | -0.062 | 1.000 | 0.344 | 0.696 |
| Bio6 | -0.207 | -0.279 | -0.343 | -0.123 | -0.139 | 0.199 | -0.062 | -0.168 | -0.168 | -0.390 | -0.073 | 0.921 | -0.043 | 0.999 | 0.294 | 0.717 |
| Bio7 | -0.229 | 0.676 | 0.322 | 0.397 | 0.577 | 0.130 | -0.179 | -0.477 | 0.310 | 0.201 | 0.044 | -0.271 | -0.275 | -0.194 | 0.719 | -0.472 |
| Bio8 | -0.226 | -0.238 | -0.324 | -0.099 | -0.104 | 0.211 | -0.071 | -0.200 | -0.146 | -0.383 | -0.071 | 0.916 | -0.059 | 1.000 | 0.345 | 0.698 |
| Bio9 | -0.206 | -0.275 | -0.343 | -0.119 | -0.133 | 0.200 | -0.064 | -0.173 | -0.168 | -0.389 | -0.074 | 0.920 | -0.046 | 1.000 | 0.303 | 0.718 |
| Bio10 | -0.223 | -0.242 | -0.328 | -0.101 | -0.106 | 0.209 | -0.073 | -0.198 | -0.151 | -0.383 | -0.071 | 0.915 | -0.060 | 1.000 | 0.340 | 0.697 |
| Bio11 | -0.208 | -0.274 | -0.341 | -0.119 | -0.133 | 0.201 | -0.062 | -0.173 | -0.165 | -0.389 | -0.074 | 0.921 | -0.044 | 1.000 | 0.304 | 0.718 |
| Bio12 | 0.294 | -0.470 | -0.426 | -0.298 | -0.331 | -0.134 | -0.072 | 0.428 | -0.457 | 0.006 | 0.042 | -0.159 | -0.054 | -0.101 | -0.722 | 0.097 |
| bio13 | 0.284 | -0.418 | -0.418 | -0.261 | -0.272 | -0.096 | -0.086 | 0.332 | -0.471 | 0.016 | 0.036 | -0.212 | -0.088 | -0.135 | -0.526 | 0.178 |
| bio14 | 0.251 | -0.423 | -0.435 | -0.306 | -0.262 | -0.103 | -0.252 | 0.302 | -0.538 | 0.007 | 0.022 | -0.183 | -0.200 | -0.038 | -0.614 | -0.054 |
| bio15 | -0.140 | 0.449 | 0.357 | 0.289 | 0.336 | 0.066 | 0.058 | -0.343 | 0.325 | 0.111 | 0.003 | -0.158 | -0.004 | -0.214 | 0.681 | -0.068 |
| bio16 | 0.288 | -0.426 | -0.412 | -0.255 | -0.285 | -0.107 | -0.046 | 0.372 | -0.452 | 0.026 | 0.039 | -0.178 | -0.056 | -0.124 | -0.574 | 0.192 |
| bio17 | 0.259 | -0.408 | -0.433 | -0.292 | -0.247 | -0.089 | -0.248 | 0.270 | -0.538 | 0.009 | 0.027 | -0.185 | -0.198 | -0.038 | -0.596 | -0.054 |
| bio18 | 0.195 | -0.336 | -0.323 | -0.235 | -0.282 | -0.057 | 0.022 | 0.381 | -0.320 | 0.037 | 0.034 | -0.164 | -0.006 | -0.141 | -0.512 | 0.221 |
| bio19 | 0.294 | -0.412 | -0.450 | -0.264 | -0.215 | -0.107 | -0.244 | 0.232 | -0.559 | 0.014 | 0.032 | -0.184 | -0.206 | -0.033 | -0.542 | -0.018 |
| **Continued** |  |  |  |  |  |  |  |  |  |  |  |  |  |  |  |  |
| Variables | Bio4 | Bio5 | Bio6 | Bio7 | Bio8 | Bio9 | Bio10 | Bio11 | Bio12 | Bio13 | Bio14 | Bio15 | Bio16 | Bio17 | Bio18 | Bio19 |
| SNDPPT | -0.260 | -0.224 | -0.207 | -0.229 | -0.226 | -0.206 | -0.223 | -0.208 | 0.294 | 0.284 | 0.251 | -0.140 | 0.288 | 0.259 | 0.195 | 0.294 |
| SLTPPT | 0.669 | -0.239 | -0.279 | 0.676 | -0.238 | -0.275 | -0.242 | -0.274 | -0.470 | -0.418 | -0.423 | 0.449 | -0.426 | -0.408 | -0.336 | -0.412 |
| PHIHOX | 0.300 | -0.327 | -0.343 | 0.322 | -0.324 | -0.343 | -0.328 | -0.341 | -0.426 | -0.418 | -0.435 | 0.357 | -0.412 | -0.433 | -0.323 | -0.450 |
| ORCDRC | 0.361 | -0.099 | -0.123 | 0.397 | -0.099 | -0.119 | -0.101 | -0.119 | -0.298 | -0.261 | -0.306 | 0.289 | -0.255 | -0.292 | -0.235 | -0.264 |
| OCSTHA | 0.555 | -0.103 | -0.139 | 0.577 | -0.104 | -0.133 | -0.106 | -0.133 | -0.331 | -0.272 | -0.262 | 0.336 | -0.285 | -0.247 | -0.282 | -0.215 |
| Globcover | 0.135 | 0.210 | 0.199 | 0.130 | 0.211 | 0.200 | 0.209 | 0.201 | -0.134 | -0.096 | -0.103 | 0.066 | -0.107 | -0.089 | -0.057 | -0.107 |
| CRFVOL | -0.220 | -0.074 | -0.062 | -0.179 | -0.071 | -0.064 | -0.073 | -0.062 | -0.072 | -0.086 | -0.252 | 0.058 | -0.046 | -0.248 | 0.022 | -0.244 |
| CLYPPT | -0.464 | -0.200 | -0.168 | -0.477 | -0.200 | -0.173 | -0.198 | -0.173 | 0.428 | 0.332 | 0.302 | -0.343 | 0.372 | 0.270 | 0.381 | 0.232 |
| CEC | 0.293 | -0.150 | -0.168 | 0.310 | -0.146 | -0.168 | -0.151 | -0.165 | -0.457 | -0.471 | -0.538 | 0.325 | -0.452 | -0.538 | -0.320 | -0.559 |
| BLD | 0.192 | -0.382 | -0.390 | 0.201 | -0.383 | -0.389 | -0.383 | -0.389 | 0.006 | 0.016 | 0.007 | 0.111 | 0.026 | 0.009 | 0.037 | 0.014 |
| Aspect | 0.057 | -0.072 | -0.073 | 0.044 | -0.071 | -0.074 | -0.071 | -0.074 | 0.042 | 0.036 | 0.022 | 0.003 | 0.039 | 0.027 | 0.034 | 0.032 |
| Alt | -0.274 | 0.915 | 0.921 | -0.271 | 0.916 | 0.920 | 0.915 | 0.921 | -0.159 | -0.212 | -0.183 | -0.158 | -0.178 | -0.185 | -0.164 | -0.184 |
| Slope | -0.318 | -0.062 | -0.043 | -0.275 | -0.059 | -0.046 | -0.060 | -0.044 | -0.054 | -0.088 | -0.200 | -0.004 | -0.056 | -0.198 | -0.006 | -0.206 |
| Bio1 | -0.169 | 1.000 | 0.999 | -0.194 | 1.000 | 1.000 | 1.000 | 1.000 | -0.101 | -0.135 | -0.038 | -0.214 | -0.124 | -0.038 | -0.141 | -0.033 |
| Bio2 | 0.658 | 0.344 | 0.294 | 0.719 | 0.345 | 0.303 | 0.340 | 0.304 | -0.722 | -0.526 | -0.614 | 0.681 | -0.574 | -0.596 | -0.512 | -0.542 |
| Bio3 | -0.527 | 0.696 | 0.717 | -0.472 | 0.698 | 0.718 | 0.697 | 0.718 | 0.097 | 0.178 | -0.054 | -0.068 | 0.192 | -0.054 | 0.221 | -0.018 |
| Bio4 | 1 | -0.141 | -0.202 | 0.990 | -0.142 | -0.195 | -0.146 | -0.194 | -0.682 | -0.574 | -0.467 | 0.607 | -0.626 | -0.451 | -0.588 | -0.439 |
| Bio5 | -0.141 | 1 | 0.998 | -0.167 | 1.000 | 0.998 | 1.000 | 0.999 | -0.122 | -0.154 | -0.052 | -0.198 | -0.143 | -0.052 | -0.160 | -0.046 |
| Bio6 | -0.202 | 0.998 | 1 | -0.228 | 0.998 | 1.000 | 0.998 | 1.000 | -0.075 | -0.115 | -0.018 | -0.238 | -0.102 | -0.019 | -0.120 | -0.015 |
| Bio7 | 0.990 | -0.167 | -0.228 | 1 | -0.168 | -0.221 | -0.172 | -0.220 | -0.710 | -0.579 | -0.518 | 0.672 | -0.632 | -0.501 | -0.590 | -0.479 |
| Bio8 | -0.142 | 1.000 | 0.998 | -0.168 | 1 | 0.998 | 1.000 | 0.999 | -0.125 | -0.156 | -0.059 | -0.194 | -0.145 | -0.059 | -0.158 | -0.053 |
| Bio9 | -0.195 | 0.998 | 1.000 | -0.221 | 0.998 | 1 | 0.999 | 1.000 | -0.079 | -0.117 | -0.021 | -0.232 | -0.104 | -0.022 | -0.123 | -0.016 |
| Bio10 | -0.146 | 1.000 | 0.998 | -0.172 | 1.000 | 0.999 | 1 | 0.999 | -0.118 | -0.150 | -0.049 | -0.201 | -0.140 | -0.049 | -0.156 | -0.043 |
| Bio11 | -0.194 | 0.999 | 1.000 | -0.220 | 0.999 | 1.000 | 0.999 | 1 | -0.082 | -0.120 | -0.025 | -0.230 | -0.107 | -0.026 | -0.125 | -0.021 |
| Bio12 | -0.682 | -0.122 | -0.075 | -0.710 | -0.125 | -0.079 | -0.118 | -0.082 | 1 | 0.913 | 0.869 | -0.679 | 0.935 | 0.862 | 0.815 | 0.864 |
| Bio13 | -0.574 | -0.154 | -0.115 | -0.579 | -0.156 | -0.117 | -0.150 | -0.120 | 0.913 | 1 | 0.731 | -0.372 | 0.984 | 0.730 | 0.896 | 0.760 |
| Bio14 | -0.467 | -0.052 | -0.018 | -0.518 | -0.059 | -0.021 | -0.049 | -0.025 | 0.869 | 0.731 | 1 | -0.767 | 0.726 | 0.992 | 0.519 | 0.976 |
| Bio15 | 0.607 | -0.198 | -0.238 | 0.672 | -0.194 | -0.232 | -0.201 | -0.230 | -0.679 | -0.372 | -0.767 | 1 | -0.414 | -0.760 | -0.277 | -0.703 |
| Bio16 | -0.626 | -0.143 | -0.102 | -0.632 | -0.145 | -0.104 | -0.140 | -0.107 | 0.935 | 0.984 | 0.726 | -0.414 | 1 | 0.721 | 0.911 | 0.747 |
| Bio17 | -0.451 | -0.052 | -0.019 | -0.501 | -0.059 | -0.022 | -0.049 | -0.026 | 0.862 | 0.730 | 0.992 | -0.760 | 0.721 | 1 | 0.517 | 0.977 |
| Bio18 | -0.588 | -0.160 | -0.120 | -0.590 | -0.158 | -0.123 | -0.156 | -0.125 | 0.815 | 0.896 | 0.519 | -0.277 | 0.911 | 0.517 | 1 | 0.509 |
| Bio19 | -0.439 | -0.046 | -0.015 | -0.479 | -0.053 | -0.016 | -0.043 | -0.021 | 0.864 | 0.760 | 0.976 | -0.703 | 0.747 | 0.977 | 0.509 | 1 |
