# Supplementary material for: Climate change may threaten habitat suitability of threatened plant species within Chinese nature reserves
Source: PeerJ. 2016 Jun 14;4:e2091. doi: 10.7717/peerj.2091 (PMC4911960; doi:10.7717/peerj.2091)
Supplement: Table S4 [file peerj-04-2091-s004.docx]

**Table S4.** Permutation importance of predictor variables. Environmental variables codes (i.e., the column headers) are given in the first column of Table S2.

| Name | BLD | CEC | CLYPPT | CRFVOL | OCSTHA | PHIHOX | SLTPPT | SNDPPT | Aspect | Slope | Globcover | Bio1 | Bio2 | Bio3 | Bio4 | Bio12 | Bio15 |
| --- | --- | --- | --- | --- | --- | --- | --- | --- | --- | --- | --- | --- | --- | --- | --- | --- | --- |
| *Magnolia officinalis* subsp. *biloba* | 2.848 | 0.430 | 0.483 | 0.376 | 7.774 | 5.541 | 0.118 | 5.391 | 1.041 | 5.801 | 2.418 | 10.506 | 8.243 | 0.851 | 14.577 | 30.324 | 3.281 |
| *Torreya fargesii* | 1.197 | 0.683 | 0.016 | 0.011 | 0.043 | 3.432 | 0.000 | 0.358 | 0.194 | 2.340 | 0.566 | 44.172 | 0.000 | 1.159 | 3.928 | 8.753 | 33.149 |
| *Pseudotaxus chienii* | 0.000 | 0.000 | 0.000 | 0.533 | 0.318 | 28.066 | 0.000 | 0.000 | 0.000 | 1.519 | 0.000 | 5.690 | 51.348 | 0.000 | 0.749 | 0.019 | 11.760 |
| *Semiliquidambar cathayensis* | 0.046 | 0.098 | 5.377 | 0.109 | 8.946 | 24.498 | 1.131 | 0.000 | 1.377 | 0.162 | 0.189 | 5.144 | 41.236 | 5.524 | 2.120 | 2.929 | 1.113 |
| *Cephalotaxus oliveri* | 0.025 | 1.795 | 1.553 | 0.443 | 0.661 | 5.112 | 0.218 | 0.370 | 0.945 | 2.021 | 0.394 | 15.801 | 0.456 | 0.984 | 1.306 | 4.331 | 63.586 |
| *Bretschneidera sinensis* | 2.761 | 0.106 | 0.710 | 0.667 | 2.095 | 2.633 | 0.029 | 1.301 | 0.596 | 1.355 | 1.472 | 14.339 | 34.945 | 2.264 | 5.615 | 19.300 | 9.814 |
| *Thuja koraiensis* | 0.000 | 0.001 | 0.000 | 0.093 | 0.000 | 1.305 | 0.003 | 0.000 | 0.010 | 0.021 | 0.001 | 51.987 | 0.000 | 26.327 | 11.264 | 8.987 | 0.000 |
| *Phellodendron chinense* | 0.019 | 0.177 | 0.000 | 0.258 | 0.445 | 0.204 | 0.019 | 0.607 | 0.030 | 7.954 | 0.317 | 22.633 | 0.535 | 10.410 | 8.611 | 30.580 | 17.202 |
| *Brasenia schreberi* | 0.716 | 1.311 | 0.013 | 0.000 | 0.000 | 0.000 | 0.005 | 1.307 | 0.000 | 0.340 | 0.000 | 8.252 | 4.476 | 39.613 | 5.396 | 31.928 | 6.644 |
| *Alsophila denticulata* | 0.274 | 0.010 | 0.047 | 0.118 | 1.269 | 1.602 | 0.001 | 0.000 | 0.017 | 7.577 | 0.025 | 0.135 | 57.746 | 0.071 | 30.360 | 0.008 | 0.740 |
| *Picea neoveitchii* | 0.001 | 0.038 | 0.115 | 0.061 | 0.105 | 0.594 | 0.000 | 0.158 | 0.040 | 0.325 | 0.014 | 30.120 | 1.498 | 20.980 | 44.301 | 0.887 | 0.763 |
| *Alsophila gigantea* | 0.000 | 0.000 | 0.062 | 0.060 | 3.971 | 90.218 | 0.874 | 0.000 | 0.375 | 0.706 | 0.066 | 0.792 | 0.214 | 0.126 | 0.161 | 0.123 | 2.252 |
| *Taxus cuspidata* | 0.163 | 0.003 | 0.000 | 0.578 | 0.024 | 0.517 | 0.106 | 0.088 | 0.002 | 1.768 | 0.450 | 53.952 | 0.898 | 19.428 | 1.366 | 17.723 | 2.935 |
| *Caryota obtusa* | 0.000 | 0.168 | 2.445 | 0.000 | 4.184 | 0.000 | 0.000 | 0.249 | 0.000 | 0.991 | 1.356 | 0.167 | 0.053 | 0.044 | 84.841 | 2.923 | 2.579 |
| *Kingdonia uniflora* | 0.425 | 0.119 | 0.010 | 0.700 | 0.000 | 0.250 | 0.507 | 0.000 | 3.875 | 14.271 | 2.749 | 27.966 | 0.000 | 0.000 | 35.899 | 0.108 | 13.122 |
| *Michelia wilsonii* | 0.003 | 0.000 | 0.101 | 0.000 | 0.000 | 0.001 | 0.000 | 0.000 | 0.008 | 0.005 | 0.014 | 7.463 | 0.009 | 41.272 | 51.017 | 0.025 | 0.084 |
| *Liriodendron chinense* | 1.231 | 0.000 | 0.207 | 0.159 | 1.730 | 3.192 | 0.000 | 3.761 | 0.480 | 0.238 | 8.881 | 30.089 | 17.382 | 4.235 | 0.228 | 0.548 | 27.639 |
| *Torreya grandis* | 1.545 | 0.547 | 1.603 | 0.037 | 0.007 | 1.221 | 0.598 | 0.001 | 2.923 | 0.680 | 0.564 | 4.249 | 1.212 | 2.916 | 3.821 | 5.821 | 72.255 |
| *Sagittaria natans* | 0.491 | 0.006 | 9.621 | 2.274 | 1.763 | 17.434 | 12.865 | 0.000 | 0.268 | 0.146 | 0.092 | 16.283 | 0.183 | 5.591 | 11.079 | 2.375 | 19.530 |
| *Fokienia hodginsii* | 0.029 | 0.000 | 0.560 | 0.242 | 0.081 | 14.108 | 1.277 | 0.000 | 0.252 | 5.639 | 0.268 | 2.898 | 62.799 | 6.811 | 0.163 | 2.589 | 2.285 |
| *Davidia involucrata* | 0.000 | 0.000 | 0.001 | 0.049 | 0.075 | 0.655 | 0.175 | 1.602 | 0.286 | 0.989 | 1.316 | 34.743 | 2.294 | 4.285 | 43.842 | 4.075 | 5.611 |
| *Davidia involucrata* var. *vilmoriniana* | 0.000 | 0.032 | 0.000 | 0.168 | 0.144 | 1.450 | 0.089 | 1.259 | 0.113 | 0.267 | 0.045 | 36.175 | 1.590 | 10.040 | 44.313 | 0.423 | 3.892 |
| *Gmelina hainanensis* | 0.007 | 0.000 | 0.000 | 3.173 | 0.000 | 9.996 | 0.000 | 0.000 | 0.000 | 0.003 | 0.000 | 80.866 | 0.000 | 3.767 | 0.000 | 1.396 | 0.792 |
| *Alsophila podophylla* | 0.004 | 0.651 | 0.055 | 0.314 | 0.128 | 0.587 | 0.000 | 0.009 | 1.650 | 3.575 | 0.205 | 66.535 | 0.004 | 3.664 | 1.483 | 16.453 | 4.686 |
| *Toona ciliata* | 0.269 | 0.000 | 7.572 | 4.173 | 3.251 | 0.053 | 0.838 | 0.124 | 3.064 | 11.562 | 4.030 | 3.418 | 15.177 | 0.258 | 1.597 | 33.981 | 10.633 |
| *Taxus wallichiana* var. *chinensis* | 0.369 | 0.144 | 1.502 | 0.536 | 0.110 | 4.632 | 0.022 | 0.434 | 0.494 | 0.702 | 0.336 | 26.947 | 3.247 | 22.190 | 33.259 | 1.183 | 3.895 |
| *Ormosia hosiei* | 0.155 | 0.038 | 1.666 | 0.808 | 0.356 | 2.494 | 1.173 | 0.003 | 0.278 | 0.414 | 2.101 | 22.403 | 2.537 | 10.263 | 7.742 | 2.104 | 45.464 |
| *Meconopsis punicea* | 0.025 | 0.000 | 0.000 | 0.000 | 0.033 | 0.457 | 0.135 | 1.827 | 0.292 | 1.910 | 1.127 | 55.147 | 0.372 | 0.061 | 33.651 | 4.939 | 0.023 |
| *Pinus koraiensis* | 0.616 | 0.032 | 1.256 | 0.658 | 0.003 | 2.002 | 0.621 | 0.019 | 0.063 | 1.038 | 0.191 | 63.757 | 4.068 | 10.850 | 0.208 | 14.496 | 0.123 |
| *Magnolia officinalis* | 2.799 | 0.730 | 0.224 | 0.168 | 2.775 | 2.459 | 3.051 | 2.703 | 0.933 | 2.890 | 2.931 | 33.176 | 3.074 | 2.192 | 15.195 | 17.194 | 7.506 |
| *Ormosia henryi* | 0.577 | 0.127 | 1.937 | 0.220 | 0.701 | 5.296 | 0.104 | 1.195 | 0.440 | 2.092 | 1.627 | 4.613 | 51.553 | 4.254 | 10.613 | 13.671 | 0.981 |
| *Pinus kwangtungensis* | 0.050 | 0.000 | 0.288 | 0.000 | 0.290 | 16.881 | 0.204 | 0.000 | 0.045 | 2.126 | 0.718 | 3.240 | 50.263 | 0.000 | 21.326 | 3.276 | 1.294 |
| *Castanopsis concinna* | 0.000 | 4.806 | 0.000 | 2.435 | 0.000 | 3.874 | 0.104 | 0.000 | 0.688 | 0.000 | 0.000 | 73.027 | 6.804 | 0.057 | 0.000 | 7.444 | 0.761 |
| *Phellodendron amurense* | 0.795 | 0.656 | 1.169 | 0.347 | 0.151 | 1.031 | 0.346 | 1.728 | 0.618 | 2.964 | 1.950 | 21.261 | 7.390 | 5.191 | 37.832 | 15.896 | 0.676 |
| *Pseudotsuga sinensis* | 0.369 | 0.139 | 0.733 | 0.001 | 0.771 | 0.063 | 0.642 | 0.000 | 0.728 | 0.117 | 0.448 | 6.727 | 0.129 | 10.848 | 28.945 | 0.130 | 49.212 |
| *Cibotium barometz* | 0.458 | 0.184 | 0.212 | 0.406 | 0.187 | 2.253 | 0.222 | 2.008 | 0.180 | 1.746 | 0.417 | 6.697 | 19.719 | 0.053 | 4.929 | 57.700 | 2.629 |
| *Pseudolarix amabilis* | 0.784 | 0.079 | 0.043 | 0.324 | 0.113 | 0.000 | 0.000 | 0.393 | 0.270 | 2.708 | 0.644 | 7.480 | 0.024 | 56.699 | 0.000 | 15.951 | 14.489 |
| *Fagopyrum dibotrys* | 1.598 | 0.413 | 1.195 | 2.501 | 0.676 | 4.041 | 0.289 | 1.283 | 1.644 | 3.184 | 2.485 | 13.174 | 4.631 | 3.407 | 8.610 | 13.200 | 37.671 |
| *Zelkova schneideriana* | 0.006 | 0.074 | 0.272 | 0.344 | 0.968 | 1.782 | 0.015 | 0.762 | 0.118 | 0.720 | 1.720 | 5.397 | 0.181 | 0.353 | 3.433 | 0.957 | 82.900 |
| *Cercidiphyllum japonicum* | 0.102 | 0.082 | 0.238 | 0.047 | 0.382 | 0.311 | 0.264 | 0.319 | 0.258 | 0.732 | 1.814 | 52.830 | 1.201 | 6.874 | 16.353 | 10.943 | 7.252 |
| *Nelumbo nucifera* | 2.150 | 2.789 | 10.791 | 0.815 | 0.903 | 0.011 | 1.137 | 0.175 | 0.090 | 2.673 | 0.076 | 4.580 | 17.957 | 11.527 | 7.386 | 33.926 | 3.016 |
| *Rhoiptelea chiliantha* | 2.014 | 7.876 | 11.044 | 0.000 | 0.000 | 49.056 | 0.000 | 0.000 | 0.342 | 0.056 | 0.000 | 0.000 | 28.684 | 0.000 | 0.000 | 0.927 | 0.000 |
| *Toona ciliata* var. *pubescens* | 1.377 | 0.085 | 0.007 | 0.027 | 0.026 | 0.708 | 0.032 | 0.144 | 0.447 | 37.353 | 1.447 | 3.083 | 1.451 | 2.009 | 0.000 | 24.764 | 27.041 |
| *Phoebe bournei* | 1.088 | 0.295 | 1.728 | 0.114 | 0.810 | 6.440 | 0.000 | 0.300 | 0.519 | 0.797 | 0.960 | 6.072 | 1.046 | 0.410 | 2.892 | 2.045 | 74.485 |
| *Aldrovanda vesiculosa* | 0.329 | 0.054 | 3.600 | 3.581 | 0.054 | 3.545 | 0.071 | 0.625 | 0.845 | 1.291 | 0.056 | 60.532 | 2.468 | 16.789 | 0.505 | 0.000 | 5.656 |
| *Taxus wallichiana* var. *mairei* | 4.898 | 2.333 | 3.595 | 3.309 | 0.264 | 16.091 | 0.389 | 0.305 | 1.569 | 3.302 | 3.133 | 19.186 | 7.364 | 6.313 | 8.920 | 7.298 | 11.732 |
| *Phoebe zhennan* | 0.001 | 0.013 | 0.001 | 0.074 | 0.092 | 0.004 | 0.002 | 0.005 | 0.011 | 2.877 | 0.403 | 35.044 | 1.388 | 10.451 | 3.967 | 35.076 | 10.591 |
| *Abies chensiensis* | 0.093 | 0.029 | 0.000 | 0.012 | 0.053 | 0.187 | 0.389 | 0.000 | 0.000 | 0.003 | 0.726 | 41.873 | 2.944 | 5.351 | 40.788 | 1.081 | 6.472 |
| *Zenia insignis* | 0.098 | 0.139 | 4.613 | 0.084 | 0.442 | 7.901 | 0.000 | 0.037 | 1.955 | 3.606 | 0.137 | 0.631 | 13.624 | 0.639 | 2.246 | 63.713 | 0.136 |
| *Machilus nanmu* | 0.842 | 0.000 | 0.128 | 0.035 | 0.401 | 0.000 | 0.000 | 0.550 | 0.041 | 1.491 | 0.001 | 3.476 | 27.174 | 5.559 | 60.077 | 0.000 | 0.227 |
| *Eurycorymbus cavaleriei* | 0.027 | 0.007 | 1.350 | 0.296 | 0.708 | 6.142 | 0.035 | 0.000 | 0.662 | 0.434 | 0.529 | 4.377 | 4.048 | 0.851 | 19.003 | 1.784 | 59.748 |
| *Euchresta japonica* | 0.000 | 0.000 | 0.000 | 1.918 | 0.000 | 22.633 | 0.000 | 0.000 | 1.133 | 4.516 | 13.242 | 9.381 | 0.083 | 10.702 | 8.503 | 0.239 | 27.651 |
| *Anisodus tanguticus* | 0.767 | 0.038 | 0.020 | 0.000 | 0.002 | 0.000 | 0.626 | 0.000 | 0.175 | 0.001 | 0.069 | 47.578 | 0.075 | 8.684 | 41.657 | 0.307 | 0.000 |
| *Dipentodon sinicus* | 1.175 | 0.000 | 14.514 | 0.000 | 0.000 | 44.103 | 0.000 | 0.000 | 0.404 | 0.000 | 0.010 | 0.000 | 1.280 | 0.000 | 38.515 | 0.000 | 0.000 |
| *Ceratopteris thalictroides* | 6.503 | 0.000 | 0.460 | 0.000 | 0.000 | 0.640 | 0.000 | 0.000 | 0.000 | 8.549 | 0.000 | 48.523 | 15.360 | 0.000 | 0.000 | 19.062 | 0.904 |
| [*Tetracentron sinense*](http://foc.eflora.cn/content.aspx?TaxonId=200008490) | 0.953 | 0.194 | 0.463 | 0.203 | 0.210 | 1.558 | 0.127 | 0.263 | 0.652 | 0.976 | 1.416 | 21.940 | 1.066 | 9.615 | 47.664 | 3.125 | 9.576 |
| *Fraxinus mandschurica* | 1.896 | 0.104 | 0.822 | 0.262 | 0.383 | 2.308 | 0.640 | 0.165 | 0.351 | 2.478 | 0.990 | 53.179 | 8.509 | 8.661 | 1.000 | 18.092 | 0.161 |
| [*Metasequoia glyptostroboides*](http://foc.eflora.cn/content.aspx?TaxonId=200005396) | 0.618 | 0.344 | 2.110 | 0.142 | 0.480 | 0.060 | 0.089 | 0.000 | 0.380 | 0.625 | 1.096 | 21.533 | 29.522 | 17.578 | 17.731 | 3.982 | 3.711 |
| *Larix mastersiana* | 0.000 | 0.021 | 0.000 | 0.000 | 0.000 | 1.285 | 3.135 | 0.000 | 2.401 | 5.530 | 0.018 | 0.035 | 7.144 | 0.679 | 69.220 | 6.114 | 4.419 |
| *Brainea insignis* | 0.000 | 2.633 | 4.530 | 4.556 | 2.190 | 10.520 | 32.579 | 0.000 | 3.717 | 1.843 | 0.239 | 25.890 | 0.004 | 0.000 | 1.108 | 7.137 | 3.055 |
| *Malania oleifera* | 0.014 | 0.000 | 0.000 | 0.136 | 0.152 | 0.000 | 0.458 | 1.437 | 0.000 | 0.000 | 0.028 | 95.615 | 0.134 | 0.000 | 1.958 | 0.039 | 0.029 |
| *Alsophila spinulosa* | 0.074 | 0.560 | 6.898 | 5.205 | 1.447 | 22.179 | 0.061 | 0.000 | 5.544 | 5.055 | 2.926 | 4.666 | 3.314 | 1.507 | 3.321 | 36.353 | 0.890 |
| *Taiwania cryptomerioides* | 2.037 | 0.162 | 15.978 | 3.110 | 0.000 | 1.187 | 0.000 | 0.000 | 0.088 | 0.000 | 0.000 | 21.710 | 31.899 | 2.862 | 5.573 | 0.000 | 15.395 |
| *Cinnamomum japonicum* | 0.000 | 0.918 | 0.000 | 5.924 | 12.789 | 0.000 | 0.000 | 0.000 | 0.736 | 0.428 | 1.611 | 0.198 | 0.040 | 2.351 | 0.247 | 0.000 | 74.761 |
| *Myriophyllum ussuriense* | 0.045 | 0.033 | 0.701 | 0.935 | 1.890 | 0.945 | 18.442 | 4.731 | 1.483 | 2.652 | 1.354 | 0.830 | 0.783 | 6.610 | 20.712 | 13.642 | 24.214 |
| *Oyama wilsonii* | 0.000 | 0.000 | 0.000 | 0.000 | 3.759 | 0.000 | 1.158 | 0.000 | 0.000 | 0.105 | 0.000 | 0.004 | 0.000 | 0.000 | 92.327 | 2.623 | 0.024 |
| *Camptotheca acuminata* | 2.392 | 1.565 | 1.671 | 1.673 | 3.020 | 9.990 | 1.214 | 2.256 | 2.114 | 3.982 | 3.283 | 12.440 | 0.549 | 5.585 | 12.941 | 13.677 | 21.648 |
| *Emmenopterys henryi* | 0.983 | 0.370 | 0.515 | 0.625 | 1.620 | 4.039 | 0.088 | 0.861 | 0.199 | 6.041 | 5.957 | 29.094 | 6.691 | 4.270 | 2.432 | 15.825 | 20.391 |
| *Alsophila metteniana* | 0.312 | 0.026 | 0.711 | 0.012 | 2.096 | 6.539 | 1.163 | 0.000 | 0.037 | 2.561 | 3.188 | 1.347 | 52.433 | 0.232 | 24.305 | 2.154 | 2.883 |
| *Triaenophora rupestris* | 0.103 | 0.261 | 0.039 | 0.048 | 0.000 | 0.020 | 0.000 | 0.413 | 0.046 | 0.730 | 0.000 | 20.253 | 0.000 | 13.760 | 45.649 | 1.517 | 17.161 |
| *Glycine soja* | 1.595 | 0.796 | 0.887 | 0.686 | 0.655 | 1.677 | 0.536 | 1.372 | 1.067 | 3.218 | 0.649 | 10.489 | 0.573 | 13.184 | 17.874 | 36.972 | 7.770 |
| *Ginkgo biloba* | 0.339 | 0.160 | 2.200 | 0.944 | 0.466 | 1.764 | 0.681 | 0.442 | 1.383 | 2.281 | 1.635 | 22.517 | 1.018 | 17.359 | 4.448 | 40.807 | 1.557 |
| *Picea brachytyla* var. *complanata* | 0.018 | 0.000 | 0.000 | 0.433 | 0.000 | 0.936 | 0.117 | 0.850 | 0.388 | 0.105 | 0.096 | 45.486 | 0.002 | 0.206 | 47.135 | 2.950 | 1.277 |
| *Cinnamomum longepaniculatum* | 0.452 | 0.105 | 0.000 | 0.069 | 0.086 | 0.158 | 0.000 | 0.032 | 0.257 | 1.241 | 0.067 | 1.159 | 1.036 | 23.058 | 71.654 | 0.609 | 0.018 |
| *Cinnamomum camphora* | 2.022 | 2.583 | 0.350 | 2.104 | 0.127 | 2.914 | 0.602 | 0.294 | 0.917 | 3.100 | 1.726 | 11.886 | 31.464 | 3.819 | 3.191 | 26.319 | 6.584 |
| *Phoebe chekiangensis* | 0.038 | 0.003 | 0.016 | 0.277 | 0.872 | 0.000 | 0.000 | 0.072 | 0.042 | 0.232 | 0.008 | 0.512 | 7.078 | 4.871 | 0.707 | 75.076 | 10.197 |
| *Zoysia sinica* | 0.815 | 0.207 | 0.041 | 0.087 | 2.614 | 0.088 | 0.487 | 0.000 | 0.102 | 0.332 | 0.002 | 5.183 | 0.041 | 30.774 | 11.921 | 0.312 | 46.995 |
| *Platycrater arguta* | 0.000 | 0.000 | 4.945 | 0.029 | 0.094 | 0.000 | 0.000 | 1.153 | 0.000 | 0.000 | 0.026 | 0.040 | 0.094 | 4.658 | 0.000 | 88.711 | 0.251 |
| *Acer amplum* subsp. *catalpifolium* | 0.000 | 0.874 | 0.000 | 0.162 | 0.512 | 0.733 | 0.261 | 0.583 | 1.189 | 2.782 | 0.031 | 3.510 | 3.981 | 8.656 | 70.987 | 5.732 | 0.007 |
| *Tilia amurensis* | 0.257 | 0.223 | 0.491 | 1.744 | 0.003 | 0.174 | 1.086 | 0.149 | 0.451 | 1.117 | 1.229 | 30.192 | 6.542 | 1.132 | 35.685 | 18.011 | 1.515 |
| *Madhuca pasquieri* | 0.000 | 5.853 | 0.000 | 0.418 | 0.892 | 36.220 | 0.000 | 0.000 | 0.252 | 2.312 | 0.759 | 43.303 | 1.957 | 0.000 | 1.794 | 0.189 | 6.054 |
| *Chosenia arbutifolia* | 1.076 | 0.044 | 0.147 | 0.360 | 0.009 | 4.023 | 0.056 | 0.327 | 0.017 | 0.068 | 0.067 | 77.024 | 2.639 | 1.604 | 2.689 | 7.564 | 2.287 |
