# Supplementary material for: Climate change may threaten habitat suitability of threatened plant species within Chinese nature reserves
Source: PeerJ. 2016 Jun 14;4:e2091. doi: 10.7717/peerj.2091 (PMC4911960; doi:10.7717/peerj.2091)
Supplement: Table S5 [file peerj-04-2091-s005.docx]

**Table S5.** Summary of changes in climate suitability for threatened plant species.

| Name | Type | 2050s-Low | 2050s-Medium | 2050s-High | 2080s-Low | 2080s-Medium | 2080s-High |
| --- | --- | --- | --- | --- | --- | --- | --- |
| *Magnolia officinalis* subsp. *biloba* | Tree | -0.181 | -0.212 | -0.270 | -0.168 | -0.258 | -0.440 |
| *Torreya fargesii* | Tree | -0.317 | -0.330 | -0.447 | -0.274 | -0.440 | -0.778 |
| *Pseudotaxus chienii* | Shrub | -0.128 | -0.176 | -0.210 | -0.142 | -0.235 | -0.331 |
| *Semiliquidambar cathayensis* | Tree | -0.246 | -0.305 | -0.390 | -0.264 | -0.409 | -0.612 |
| *Cephalotaxus oliveri* | Shrub | -0.511 | -0.488 | -0.646 | -0.491 | -0.646 | -0.892 |
| *Bretschneidera sinensis* | Tree | -0.281 | -0.337 | -0.427 | -0.288 | -0.408 | -0.645 |
| *Thuja koraiensis* | Tree | -0.425 | -0.571 | -0.727 | -0.407 | -0.761 | -0.989 |
| *Phellodendron chinense* | Tree | -0.348 | -0.360 | -0.474 | -0.306 | -0.461 | -0.730 |
| *Brasenia schreberi* | Herb | -0.125 | -0.178 | -0.199 | -0.106 | -0.206 | -0.301 |
| *Alsophila denticulata* | Fern | -0.116 | -0.174 | -0.186 | -0.123 | -0.225 | -0.265 |
| *Picea neoveitchii* | Tree | -0.500 | -0.608 | -0.663 | -0.508 | -0.695 | -0.898 |
| *Alsophila gigantea* | Fern | 0.010 | 0.016 | 0.040 | 0.021 | 0.024 | 0.047 |
| *Taxus cuspidata* | Tree | -0.093 | -0.169 | -0.204 | -0.058 | -0.235 | -0.504 |
| *Caryota obtusa* | Tree | 0.053 | 0.054 | 0.019 | 0.034 | 0.065 | 0.068 |
| *Kingdonia uniflora* | Herb | 0.022 | 0.003 | -0.010 | 0.046 | 0.001 | -0.059 |
| *Michelia wilsonii* | Tree | -0.715 | -0.804 | -0.852 | -0.693 | -0.842 | -0.971 |
| *Liriodendron chinense* | Tree | -0.328 | -0.359 | -0.455 | -0.315 | -0.463 | -0.705 |
| *Torreya grandis* | Tree | -0.169 | -0.166 | -0.266 | -0.138 | -0.182 | -0.380 |
| *Sagittaria natans* | Herb | 0.034 | 0.057 | 0.096 | 0.073 | 0.060 | -0.091 |
| *Fokienia hodginsii* | Tree | -0.185 | -0.229 | -0.298 | -0.167 | -0.312 | -0.434 |
| *Davidia involucrata* | Tree | -0.432 | -0.468 | -0.570 | -0.401 | -0.545 | -0.789 |
| *Davidia involucrata* var. *vilmoriniana* | Tree | -0.463 | -0.531 | -0.627 | -0.441 | -0.618 | -0.864 |
| *Gmelina hainanensis* | Tree | 0.244 | 0.276 | 0.326 | 0.255 | 0.328 | 0.429 |
| *Alsophila podophylla* | Fern | 0.061 | 0.077 | 0.062 | 0.103 | 0.096 | 0.134 |
| *Toona ciliata* | Tree | -0.093 | -0.125 | -0.142 | -0.096 | -0.159 | -0.229 |
| *Taxus wallichiana* var. *chinensis* | Tree | -0.291 | -0.325 | -0.399 | -0.283 | -0.396 | -0.635 |
| *Ormosia hosiei* | Tree | -0.255 | -0.260 | -0.378 | -0.214 | -0.353 | -0.646 |
| *Meconopsis punicea* | Herb | -0.033 | -0.057 | -0.115 | -0.026 | -0.095 | -0.300 |
| *Pinus koraiensis* | Tree | -0.027 | -0.111 | -0.171 | -0.014 | -0.192 | -0.546 |
| *Magnolia officinalis* | Tree | -0.234 | -0.264 | -0.385 | -0.217 | -0.327 | -0.621 |
| *Ormosia henryi* | Tree | -0.194 | -0.239 | -0.283 | -0.179 | -0.308 | -0.407 |
| *Pinus kwangtungensis* | Tree | -0.441 | -0.517 | -0.594 | -0.475 | -0.644 | -0.808 |
| *Castanopsis concinna* | Tree | 0.177 | 0.200 | 0.226 | 0.182 | 0.232 | 0.317 |
| *Phellodendron amurense* | Tree | 0.001 | -0.027 | -0.075 | 0.003 | -0.048 | -0.160 |
| *Pseudotsuga sinensis* | Tree | -0.486 | -0.490 | -0.602 | -0.431 | -0.579 | -0.846 |
| *Cibotium barometz* | Fern | -0.129 | -0.184 | -0.249 | -0.101 | -0.217 | -0.325 |
| *Pseudolarix amabilis* | Tree | -0.400 | -0.458 | -0.586 | -0.384 | -0.592 | -0.817 |
| *Fagopyrum dibotrys* | Herb | -0.258 | -0.266 | -0.371 | -0.242 | -0.327 | -0.579 |
| *Zelkova schneideriana* | Tree | -0.284 | -0.268 | -0.395 | -0.259 | -0.361 | -0.645 |
| *Cercidiphyllum japonicum* | Tree | -0.299 | -0.318 | -0.401 | -0.282 | -0.396 | -0.617 |
| *Nelumbo nucifera* | Herb | 0.012 | -0.008 | 0.028 | 0.038 | 0.000 | 0.049 |
| *Rhoiptelea chiliantha* | Tree | -0.070 | -0.080 | -0.079 | -0.069 | -0.122 | -0.113 |
| *Toona ciliata* var. *pubescens* | Tree | -0.037 | -0.016 | -0.032 | -0.028 | -0.027 | -0.027 |
| *Phoebe bournei* | Tree | -0.273 | -0.294 | -0.429 | -0.276 | -0.392 | -0.669 |
| *Aldrovanda vesiculosa* | Herb | -0.189 | -0.221 | -0.328 | -0.144 | -0.309 | -0.746 |
| *Taxus wallichiana* var. *mairei* | Tree | -0.236 | -0.271 | -0.352 | -0.226 | -0.339 | -0.557 |
| *Phoebe zhennan* | Tree | -0.517 | -0.546 | -0.670 | -0.495 | -0.644 | -0.872 |
| *Abies chensiensis* | Tree | -0.291 | -0.384 | -0.422 | -0.282 | -0.463 | -0.666 |
| *Zenia insignis* | Tree | -0.089 | -0.124 | -0.125 | -0.144 | -0.221 | -0.264 |
| *Machilus nanmu* | Tree | -0.215 | -0.279 | -0.301 | -0.218 | -0.317 | -0.451 |
| *Eurycorymbus cavaleriei* | Tree | -0.397 | -0.394 | -0.541 | -0.416 | -0.556 | -0.832 |
| *Euchresta japonica* | Shrub | -0.164 | -0.172 | -0.239 | -0.180 | -0.231 | -0.367 |
| *Anisodus tanguticus* | Herb | -0.299 | -0.316 | -0.372 | -0.295 | -0.411 | -0.592 |
| *Dipentodon sinicus* | Shrub | -0.014 | -0.012 | -0.026 | -0.009 | -0.017 | -0.031 |
| *Ceratopteris thalictroides* | Fern | 0.154 | 0.152 | 0.176 | 0.160 | 0.190 | 0.272 |
| [*Tetracentron sinense*](http://foc.eflora.cn/content.aspx?TaxonId=200008490) | Tree | -0.260 | -0.309 | -0.345 | -0.267 | -0.358 | -0.577 |
| *Fraxinus mandschurica* | Tree | -0.126 | -0.186 | -0.206 | -0.119 | -0.251 | -0.411 |
| [*Metasequoia glyptostroboides*](http://foc.eflora.cn/content.aspx?TaxonId=200005396) | Tree | -0.225 | -0.292 | -0.355 | -0.225 | -0.370 | -0.593 |
| *Larix mastersiana* | Tree | 0.027 | 0.032 | 0.046 | 0.088 | 0.010 | 0.017 |
| *Brainea insignis* | Fern | 0.067 | 0.073 | 0.082 | 0.072 | 0.094 | 0.138 |
| *Malania oleifera* | Tree | 0.503 | 0.503 | 0.504 | 0.503 | 0.504 | 0.504 |
| *Alsophila spinulosa* | Fern | 0.021 | 0.024 | 0.021 | 0.008 | 0.009 | 0.002 |
| *Taiwania cryptomerioides* | Tree | -0.282 | -0.262 | -0.361 | -0.228 | -0.359 | -0.556 |
| *Cinnamomum japonicum* | Tree | -0.024 | -0.034 | -0.009 | -0.009 | -0.015 | 0.007 |
| *Myriophyllum ussuriense* | Herb | 0.003 | 0.035 | 0.045 | 0.029 | 0.036 | -0.062 |
| *Oyama wilsonii* | Shrub | 0.010 | -0.002 | -0.025 | -0.001 | -0.026 | -0.065 |
| *Camptotheca acuminata* | Tree | -0.191 | -0.180 | -0.286 | -0.203 | -0.267 | -0.492 |
| *Emmenopterys henryi* | Tree | -0.275 | -0.299 | -0.397 | -0.253 | -0.373 | -0.627 |
| *Alsophila metteniana* | Fern | -0.197 | -0.242 | -0.288 | -0.226 | -0.338 | -0.501 |
| *Triaenophora rupestris* | Herb | -0.520 | -0.563 | -0.618 | -0.490 | -0.617 | -0.837 |
| *Glycine soja* | Herb | -0.111 | -0.119 | -0.156 | -0.086 | -0.158 | -0.265 |
| *Ginkgo biloba* | Tree | -0.345 | -0.376 | -0.447 | -0.324 | -0.457 | -0.640 |
| *Picea brachytyla* var. *complanata* | Tree | -0.226 | -0.257 | -0.349 | -0.199 | -0.311 | -0.571 |
| *Cinnamomum longepaniculatum* | Tree | -0.303 | -0.387 | -0.436 | -0.329 | -0.480 | -0.704 |
| *Cinnamomum camphora* | Tree | -0.179 | -0.196 | -0.231 | -0.143 | -0.218 | -0.233 |
| *Phoebe chekiangensis* | Tree | 0.059 | 0.091 | 0.025 | 0.101 | 0.159 | 0.166 |
| *Zoysia sinica* | Herb | -0.349 | -0.364 | -0.397 | -0.409 | -0.507 | -0.488 |
| *Platycrater arguta* | Shrub | 0.014 | -0.004 | -0.057 | 0.040 | 0.071 | 0.050 |
| *Acer amplum* subsp. *catalpifolium* | Tree | -0.095 | -0.133 | -0.149 | -0.109 | -0.182 | -0.292 |
| *Tilia amurensis* | Tree | -0.059 | -0.125 | -0.207 | -0.054 | -0.163 | -0.372 |
| *Madhuca pasquieri* | Tree | 0.145 | 0.177 | 0.221 | 0.158 | 0.211 | 0.301 |
| *Chosenia arbutifolia* | Tree | -0.096 | -0.154 | -0.272 | -0.084 | -0.240 | -0.535 |
