# Supplementary material for: Climate change may threaten habitat suitability of threatened plant species within Chinese nature reserves
Source: PeerJ. 2016 Jun 14;4:e2091. doi: 10.7717/peerj.2091 (PMC4911960; doi:10.7717/peerj.2091)
Supplement: Table S6 [file peerj-04-2091-s006.docx]

**Table S6.** Summary of changes in climate suitability for threatened plant species according to nature reserve.

| Nature | 2050s-Low | 2050s-Medium | 2050s-High | 2080s-Low | 2080s-Medium | 2080s-High |
| --- | --- | --- | --- | --- | --- | --- |
| Banqiao | -0.159 | -0.193 | -0.287 | -0.126 | -0.242 | -0.537 |
| Gujingyuan | -0.243 | -0.299 | -0.359 | -0.250 | -0.331 | -0.536 |
| Qingliangfeng | -0.334 | -0.358 | -0.434 | -0.286 | -0.388 | -0.585 |
| Songshan | -0.016 | 0.015 | -0.109 | 0.078 | -0.040 | -0.127 |
| Daiyunshan | -0.405 | -0.434 | -0.485 | -0.418 | -0.479 | -0.577 |
| E'meifeng | -0.383 | -0.394 | -0.513 | -0.410 | -0.452 | -0.587 |
| Longqishan | -0.274 | -0.274 | -0.432 | -0.309 | -0.361 | -0.577 |
| Minjiangyuan | -0.154 | -0.152 | -0.239 | -0.175 | -0.230 | -0.332 |
| Tingjiangyuan | -0.494 | -0.516 | -0.618 | -0.517 | -0.554 | -0.674 |
| Xiongjianghuangchulin | -0.358 | -0.383 | -0.436 | -0.348 | -0.413 | -0.539 |
| Lianhuashan | 0.016 | 0.009 | 0.053 | 0.061 | -0.020 | -0.068 |
| Qinzhouzhenxishuishengyeshengdongwu | -0.249 | -0.379 | -0.379 | -0.213 | -0.427 | -0.642 |
| Taizishan | 0.043 | 0.019 | -0.019 | 0.046 | 0.008 | -0.148 |
| Yuhe | -0.172 | -0.326 | -0.353 | -0.128 | -0.314 | -0.542 |
| Haifengniaolei | 0.130 | 0.154 | 0.169 | 0.131 | 0.172 | 0.205 |
| Lianzhoutianxin | -0.230 | -0.266 | -0.357 | -0.257 | -0.354 | -0.502 |
| Luokeng'exi | -0.116 | -0.169 | -0.209 | -0.141 | -0.217 | -0.298 |
| Shimentai | -0.118 | -0.151 | -0.151 | -0.129 | -0.169 | -0.176 |
| Xiangtoushan | -0.022 | -0.018 | -0.019 | -0.008 | -0.027 | -0.013 |
| Yunkaishan | -0.031 | -0.064 | -0.040 | -0.059 | -0.081 | -0.054 |
| Bangliangchangbiyuan | -0.200 | -0.185 | -0.238 | -0.174 | -0.259 | -0.299 |
| Chongzuobaitouyehou | 0.074 | 0.083 | 0.037 | 0.075 | -0.151 | 0.045 |
| Daguishan'exi | -0.030 | -0.045 | -0.006 | -0.063 | -0.077 | -0.055 |
| Dayaoshan | -0.226 | -0.238 | -0.278 | -0.262 | -0.348 | -0.408 |
| Encheng | 0.146 | 0.145 | 0.135 | 0.174 | 0.117 | 0.167 |
| Fangchengjinhuacha | -0.028 | -0.025 | -0.015 | -0.036 | -0.072 | -0.021 |
| Huaping | -0.199 | -0.240 | -0.332 | -0.232 | -0.368 | -0.560 |
| Jiuwanshan | -0.311 | -0.311 | -0.385 | -0.316 | -0.415 | -0.501 |
| Qichong | -0.126 | -0.134 | -0.170 | -0.126 | -0.166 | -0.168 |
| Shiwandashan | -0.039 | -0.039 | -0.037 | -0.044 | -0.091 | -0.037 |
| Yinzhulaoshanziyuanlengshan | -0.018 | -0.067 | -0.096 | -0.003 | -0.119 | -0.318 |
| Yuanbaoshan | -0.389 | -0.375 | -0.475 | -0.378 | -0.481 | -0.606 |
| Dashahe | -0.395 | -0.407 | -0.554 | -0.384 | -0.503 | -0.747 |
| Fodingshan | -0.482 | -0.468 | -0.653 | -0.426 | -0.578 | -0.860 |
| Leigongshan | -0.357 | -0.312 | -0.468 | -0.284 | -0.443 | -0.655 |
| Yinggeling | -0.087 | -0.104 | -0.122 | -0.104 | -0.128 | -0.139 |
| Changlihuangjinhai'an | -0.027 | -0.324 | -0.202 | -0.229 | -0.447 | -0.450 |
| Qingyazhai | -0.209 | -0.287 | -0.421 | -0.192 | -0.467 | -0.521 |
| Tuoliang | 0.050 | 0.057 | -0.038 | 0.060 | -0.035 | -0.138 |
| Xiaowutaishan | -0.138 | -0.119 | -0.230 | -0.048 | -0.209 | -0.325 |
| Baotianman | -0.291 | -0.403 | -0.450 | -0.278 | -0.490 | -0.599 |
| He'nandabieshan | -0.077 | -0.133 | -0.245 | -0.084 | -0.236 | -0.475 |
| Gaoleshan | -0.410 | -0.468 | -0.463 | -0.371 | -0.497 | -0.508 |
| Huangheshidi | -0.333 | -0.440 | -0.511 | -0.299 | -0.550 | -0.617 |
| Jigongshan | -0.456 | -0.497 | -0.590 | -0.450 | -0.574 | -0.741 |
| Beijicun | 0.017 | 0.031 | -0.059 | 0.053 | 0.031 | -0.131 |
| Chuonahe | 0.222 | 0.197 | 0.137 | 0.180 | 0.191 | 0.063 |
| Daxiagu | -0.045 | -0.095 | -0.080 | -0.024 | -0.145 | -0.293 |
| Dongbeihu | -0.057 | -0.100 | -0.150 | -0.048 | -0.144 | -0.338 |
| Dongfanghong | -0.049 | -0.104 | -0.136 | -0.051 | -0.131 | -0.296 |
| Duobuku'er | 0.035 | 0.059 | -0.009 | 0.063 | 0.047 | -0.182 |
| Fenglin | -0.037 | -0.076 | -0.115 | -0.025 | -0.116 | -0.294 |
| Fenghuangshan | -0.034 | -0.074 | -0.092 | 0.001 | -0.120 | -0.340 |
| Gongbielahe | 0.075 | 0.054 | -0.006 | 0.069 | 0.066 | -0.139 |
| Lingfeng | 0.053 | 0.096 | 0.106 | 0.081 | 0.150 | 0.119 |
| Maolan'gou | 0.029 | 0.023 | -0.004 | 0.053 | 0.011 | -0.138 |
| Mingshui | -0.094 | -0.193 | -0.158 | -0.026 | -0.166 | -0.153 |
| Mudanfeng | -0.027 | -0.119 | -0.142 | -0.007 | -0.188 | -0.469 |
| Pingdingshan | -0.022 | -0.062 | -0.084 | -0.017 | -0.108 | -0.270 |
| Qixingfengdongbeihu | -0.031 | -0.068 | -0.104 | -0.016 | -0.137 | -0.292 |
| Sanhuanpao | -0.084 | -0.065 | -0.128 | -0.075 | -0.105 | -0.287 |
| Shankou | 0.063 | 0.026 | -0.026 | 0.071 | 0.001 | -0.180 |
| Taipinggou | 0.049 | 0.031 | 0.005 | 0.084 | -0.007 | -0.179 |
| Wuyiling | 0.008 | -0.003 | -0.001 | 0.034 | -0.009 | -0.180 |
| Wuyu'erhe | 0.229 | 0.145 | 0.235 | 0.239 | 0.170 | 0.313 |
| Wudalianchihuoshandizhiyiji | 0.028 | 0.004 | -0.049 | 0.043 | -0.019 | -0.145 |
| Xiaobeihu | -0.023 | -0.065 | -0.060 | -0.010 | -0.105 | -0.263 |
| Xinqingbaitouhe | 0.041 | 0.026 | -0.011 | 0.051 | -0.011 | -0.169 |
| Youhao | -0.014 | -0.038 | -0.062 | 0.010 | -0.068 | -0.282 |
| Zhongyangzhanheizuisongji | 0.353 | 0.305 | 0.204 | 0.330 | 0.288 | 0.040 |
| Badongjinsihou | -0.213 | -0.233 | -0.337 | -0.189 | -0.319 | -0.569 |
| Duheyuan | -0.212 | -0.238 | -0.315 | -0.184 | -0.292 | -0.616 |
| Hubeidabieshan | -0.154 | -0.206 | -0.302 | -0.169 | -0.280 | -0.509 |
| Mulinzi | -0.077 | -0.090 | -0.150 | -0.092 | -0.161 | -0.502 |
| Nanhe | -0.575 | -0.589 | -0.683 | -0.516 | -0.676 | -0.807 |
| Qizimeishan | -0.633 | -0.637 | -0.710 | -0.621 | -0.698 | -0.822 |
| Saiwudang | -0.293 | -0.316 | -0.398 | -0.236 | -0.372 | -0.602 |
| Sanxiadalaoling | -0.295 | -0.305 | -0.408 | -0.267 | -0.385 | -0.625 |
| Shennongjia | -0.190 | -0.213 | -0.258 | -0.174 | -0.255 | -0.468 |
| Shibalichangxia | -0.084 | -0.094 | -0.145 | -0.062 | -0.129 | -0.359 |
| Wudaoxia | -0.653 | -0.679 | -0.764 | -0.604 | -0.745 | -0.891 |
| Xianfengzhongjianheda'ni | -0.529 | -0.537 | -0.667 | -0.521 | -0.653 | -0.857 |
| Xingdoushan | -0.333 | -0.350 | -0.472 | -0.321 | -0.459 | -0.692 |
| Ye'rengu | -0.214 | -0.232 | -0.328 | -0.171 | -0.308 | -0.599 |
| Baiyunshan | -0.386 | -0.430 | -0.568 | -0.361 | -0.537 | -0.746 |
| Dong'anshunhuangshan | -0.104 | -0.160 | -0.259 | -0.122 | -0.242 | -0.415 |
| Dongdongtinghu | -0.420 | -0.448 | -0.475 | -0.444 | -0.540 | -0.535 |
| Gaowangjie | -0.437 | -0.462 | -0.586 | -0.443 | -0.590 | -0.750 |
| Hupingshan | -0.224 | -0.249 | -0.361 | -0.218 | -0.349 | -0.649 |
| Jintongshan | -0.304 | -0.358 | -0.430 | -0.319 | -0.442 | -0.612 |
| Jiuyishan | -0.177 | -0.202 | -0.279 | -0.186 | -0.293 | -0.474 |
| Wuyunjie | -0.310 | -0.421 | -0.462 | -0.296 | -0.410 | -0.592 |
| Xidongtinghu | -0.059 | -0.056 | -0.042 | -0.018 | -0.027 | 0.025 |
| Baishanyuanshe | -0.097 | -0.196 | -0.200 | -0.058 | -0.232 | -0.370 |
| Boluohu | -0.007 | -0.197 | -0.149 | -0.178 | -0.017 | 0.000 |
| Hunchundongbeihu | -0.116 | -0.188 | -0.239 | -0.101 | -0.243 | -0.435 |
| Ji'an | -0.318 | -0.434 | -0.482 | -0.260 | -0.507 | -0.709 |
| Jingyu | -0.164 | -0.273 | -0.282 | -0.139 | -0.351 | -0.601 |
| Shihu | -0.017 | -0.099 | -0.143 | 0.011 | -0.175 | -0.433 |
| Wangqing | -0.059 | -0.118 | -0.159 | -0.031 | -0.156 | -0.384 |
| Yanminghu | -0.064 | -0.115 | -0.085 | -0.042 | -0.141 | -0.218 |
| Yanchengshidizhenqin | -0.283 | -0.318 | -0.349 | -0.257 | -0.350 | -0.466 |
| Ganjiangyuan | -0.326 | -0.340 | -0.444 | -0.336 | -0.459 | -0.595 |
| Jiulingshan | -0.127 | -0.174 | -0.228 | -0.087 | -0.189 | -0.409 |
| Lushan | -0.291 | -0.302 | -0.351 | -0.234 | -0.305 | -0.389 |
| Qiyunshan | -0.161 | -0.175 | -0.246 | -0.150 | -0.240 | -0.432 |
| Tongboshan | -0.155 | -0.166 | -0.244 | -0.161 | -0.228 | -0.397 |
| Wuyuansenlinniaolei | -0.222 | -0.240 | -0.322 | -0.214 | -0.300 | -0.479 |
| Yangjifeng | -0.315 | -0.334 | -0.440 | -0.335 | -0.427 | -0.602 |
| Bailangshan | -0.274 | -0.324 | -0.321 | -0.160 | -0.453 | -0.585 |
| Daheishan | 0.104 | 0.106 | -0.167 | 0.201 | -0.018 | -0.162 |
| Hongluoshan | 0.051 | -0.062 | -0.420 | -0.110 | -0.228 | -0.521 |
| Louzishan | -0.234 | -0.268 | -0.339 | -0.094 | -0.413 | -0.561 |
| Nulu'erhushan | 0.107 | -0.028 | -0.177 | 0.162 | -0.015 | -0.189 |
| Qinglonghe | -0.267 | -0.359 | -0.321 | -0.219 | -0.465 | -0.574 |
| Yalujiangkoushidi | -0.343 | -0.436 | -0.458 | -0.267 | -0.508 | -0.704 |
| Zhanggutai | -0.046 | -0.131 | -0.130 | 0.021 | -0.193 | -0.262 |
| Alu | 0.145 | 0.164 | 0.187 | 0.157 | 0.207 | 0.188 |
| Bilahe | 0.086 | 0.092 | 0.000 | 0.072 | 0.050 | -0.119 |
| Gaogesitaihanwula | 0.198 | 0.152 | -0.119 | 0.273 | 0.254 | -0.177 |
| Hanshan | 0.520 | 0.531 | 0.387 | 0.561 | 0.563 | 0.564 |
| Hanma | 0.161 | 0.188 | 0.204 | 0.174 | 0.224 | 0.190 |
| Qingshan | 0.148 | 0.117 | 0.001 | 0.186 | 0.135 | 0.018 |
| Wulanba | 0.919 | 0.881 | 0.611 | 0.844 | 0.862 | 0.405 |
| Datongbeichuanheyuanqu | -0.360 | -0.368 | -0.401 | -0.359 | -0.444 | -0.512 |
| Huanghesanjiaozhou | -0.292 | -0.375 | -0.374 | -0.303 | -0.409 | -0.505 |
| Heichashan | -0.112 | -0.095 | -0.119 | -0.088 | -0.151 | -0.068 |
| Lingkongshan | 0.290 | -0.075 | 0.266 | 0.362 | 0.172 | 0.272 |
| Guanyinshan | -0.118 | -0.206 | -0.241 | -0.111 | -0.265 | -0.569 |
| Hanchenghuanglongshanhemaji | -0.157 | -0.231 | -0.280 | -0.140 | -0.301 | -0.489 |
| Huangbaiyuan | -0.114 | -0.150 | -0.156 | -0.105 | -0.200 | -0.350 |
| Huanglongshanhemaji | 0.013 | -0.030 | -0.060 | 0.033 | -0.079 | -0.301 |
| Lueyuangzhenxishuishengdongwu | -0.185 | -0.241 | -0.335 | -0.187 | -0.347 | -0.663 |
| Micangshan | -0.205 | -0.251 | -0.304 | -0.208 | -0.310 | -0.602 |
| Motianling | -0.130 | -0.185 | -0.240 | -0.125 | -0.233 | -0.528 |
| Pingheliang | -0.197 | -0.305 | -0.326 | -0.239 | -0.320 | -0.503 |
| Taibaishan | -0.171 | -0.249 | -0.249 | -0.189 | -0.247 | -0.398 |
| Taibaixushuihe | -0.137 | -0.195 | -0.198 | -0.130 | -0.213 | -0.360 |
| Wuliangshan | -0.342 | -0.510 | -0.568 | -0.387 | -0.536 | -0.755 |
| Zhouzhilaoxiancheng | -0.095 | -0.119 | -0.126 | -0.071 | -0.167 | -0.336 |
| Anzihe | -0.143 | -0.142 | -0.242 | -0.126 | -0.223 | -0.441 |
| Baihe | -0.219 | -0.255 | -0.208 | -0.130 | -0.276 | -0.300 |
| Caopo | -0.243 | -0.246 | -0.249 | -0.242 | -0.319 | -0.309 |
| Gexigou | -0.095 | -0.148 | -0.242 | -0.076 | -0.201 | -0.455 |
| Heizhugou | -0.372 | -0.314 | -0.399 | -0.309 | -0.372 | -0.495 |
| Jiudingshan | -0.291 | -0.309 | -0.416 | -0.241 | -0.418 | -0.650 |
| Laojunshan | -0.517 | -0.528 | -0.627 | -0.494 | -0.591 | -0.728 |
| Liziping | -0.361 | -0.348 | -0.437 | -0.380 | -0.443 | -0.641 |
| Nuoshuihezhenxishuishengdongwu | -0.172 | -0.265 | -0.326 | -0.178 | -0.336 | -0.599 |
| Qianfoshan | -0.126 | -0.131 | -0.210 | -0.037 | -0.214 | -0.473 |
| Xiaozhaizigou | -0.161 | -0.114 | -0.128 | -0.066 | -0.175 | -0.322 |
| Xuebaoding | -0.151 | -0.154 | -0.163 | -0.076 | -0.205 | -0.277 |
| Ailaoshan | -0.605 | -0.331 | -0.714 | -0.477 | -0.622 | -0.887 |
| Daweishan | -0.134 | -0.151 | -0.148 | -0.119 | -0.199 | -0.170 |
| Jiaozishan | -0.241 | -0.182 | -0.260 | -0.197 | -0.200 | -0.268 |
| Lvchunhuanglianshan | -0.086 | -0.077 | -0.096 | -0.038 | -0.144 | -0.185 |
| Nangunhe | -0.051 | -0.072 | -0.110 | -0.065 | -0.055 | -0.068 |
| Tongbiguan | -0.025 | -0.014 | -0.005 | -0.024 | -0.019 | -0.007 |
| Wenshan | -0.209 | -0.148 | -0.294 | -0.131 | -0.250 | -0.348 |
| Wumengshan | -0.460 | -0.478 | -0.670 | -0.438 | -0.558 | -0.775 |
| Yuanjiang | -0.170 | -0.006 | -0.205 | -0.015 | -0.152 | -0.269 |
| Yunlongtianchi | -0.213 | -0.203 | -0.266 | -0.203 | -0.255 | -0.355 |
| Wuyanling | -0.081 | -0.118 | -0.169 | -0.086 | -0.177 | -0.291 |
| Changxingyangzi'e | -0.272 | -0.299 | -0.365 | -0.203 | -0.314 | -0.404 |
| Dabashan | -0.262 | -0.309 | -0.362 | -0.248 | -0.352 | -0.605 |
| Jinfoshan | -0.464 | -0.489 | -0.609 | -0.456 | -0.560 | -0.760 |
| Wulipo | -0.305 | -0.333 | -0.398 | -0.286 | -0.393 | -0.607 |
| Xuebaoshan | -0.217 | -0.252 | -0.311 | -0.209 | -0.311 | -0.583 |
