# Supplementary material for: Climate change may threaten habitat suitability of threatened plant species within Chinese nature reserves
Source: PeerJ. 2016 Jun 14;4:e2091. doi: 10.7717/peerj.2091 (PMC4911960; doi:10.7717/peerj.2091)
Supplement: Table S7 [file peerj-04-2091-s007.docx]

**Table S7.** Summary of overlap between current and future climatically suitable habitat for threatened plant species.

| Name | Type | 2050s-Low | 2050s-Medium | 2050s-High | 2080s-Low | 2080s-Medium | 2080s-High |
| --- | --- | --- | --- | --- | --- | --- | --- |
| *Magnolia officinalis* subsp. *biloba* | Tree | 0.904 | 0.895 | 0.881 | 0.904 | 0.878 | 0.840 |
| *Torreya fargesii* | Tree | 0.824 | 0.815 | 0.764 | 0.835 | 0.765 | 0.637 |
| *Pseudotaxus chienii* | Shrub | 0.974 | 0.968 | 0.965 | 0.972 | 0.965 | 0.961 |
| *Semiliquidambar cathayensis* | Tree | 0.892 | 0.885 | 0.864 | 0.893 | 0.850 | 0.795 |
| *Cephalotaxus oliveri* | Shrub | 0.740 | 0.739 | 0.675 | 0.744 | 0.651 | 0.511 |
| *Bretschneidera sinensis* | Tree | 0.852 | 0.827 | 0.794 | 0.844 | 0.800 | 0.719 |
| *Thuja koraiensis* | Tree | 0.752 | 0.696 | 0.623 | 0.761 | 0.619 | 0.524 |
| *Phellodendron chinense* | Tree | 0.817 | 0.808 | 0.746 | 0.826 | 0.757 | 0.594 |
| *Brasenia schreberi* | Herb | 0.962 | 0.952 | 0.946 | 0.964 | 0.951 | 0.929 |
| *Alsophila denticulata* | Fern | 0.947 | 0.931 | 0.929 | 0.948 | 0.935 | 0.898 |
| *Picea neoveitchii* | Tree | 0.721 | 0.668 | 0.619 | 0.719 | 0.613 | 0.479 |
| *Alsophila gigantea* | Fern | 0.994 | 0.995 | 0.992 | 0.994 | 0.994 | 0.988 |
| *Taxus cuspidata* | Tree | 0.950 | 0.935 | 0.923 | 0.956 | 0.921 | 0.849 |
| *Caryota obtusa* | Tree | 0.980 | 0.984 | 0.990 | 0.982 | 0.983 | 0.980 |
| *Kingdonia uniflora* | Herb | 0.980 | 0.982 | 0.983 | 0.981 | 0.981 | 0.975 |
| *Michelia wilsonii* | Tree | 0.607 | 0.576 | 0.545 | 0.620 | 0.531 | 0.403 |
| *Liriodendron chinense* | Tree | 0.826 | 0.822 | 0.776 | 0.831 | 0.777 | 0.685 |
| *Torreya grandis* | Tree | 0.899 | 0.900 | 0.863 | 0.904 | 0.884 | 0.827 |
| *Sagittaria natans* | Herb | 0.953 | 0.951 | 0.940 | 0.959 | 0.944 | 0.886 |
| *Fokienia hodginsii* | Tree | 0.899 | 0.893 | 0.888 | 0.904 | 0.879 | 0.851 |
| *Davidia involucrata* | Tree | 0.753 | 0.733 | 0.652 | 0.778 | 0.687 | 0.484 |
| *Davidia involucrata* var. *vilmoriniana* | Tree | 0.745 | 0.699 | 0.623 | 0.760 | 0.637 | 0.416 |
| *Gmelina hainanensis* | Tree | 0.957 | 0.954 | 0.945 | 0.954 | 0.942 | 0.921 |
| *Alsophila podophylla* | Fern | 0.956 | 0.957 | 0.962 | 0.941 | 0.938 | 0.937 |
| *Toona ciliata* | Tree | 0.949 | 0.944 | 0.936 | 0.941 | 0.929 | 0.914 |
| *Taxus wallichiana* var. *chinensis* | Tree | 0.855 | 0.826 | 0.789 | 0.846 | 0.797 | 0.628 |
| *Ormosia hosiei* | Tree | 0.821 | 0.817 | 0.753 | 0.818 | 0.767 | 0.649 |
| *Meconopsis punicea* | Herb | 0.952 | 0.948 | 0.929 | 0.954 | 0.930 | 0.850 |
| *Pinus koraiensis* | Tree | 0.918 | 0.891 | 0.873 | 0.922 | 0.860 | 0.733 |
| *Magnolia officinalis* | Tree | 0.819 | 0.821 | 0.756 | 0.827 | 0.776 | 0.668 |
| *Ormosia henryi* | Tree | 0.918 | 0.913 | 0.901 | 0.919 | 0.891 | 0.872 |
| *Pinus kwangtungensis* | Tree | 0.868 | 0.849 | 0.823 | 0.861 | 0.815 | 0.757 |
| *Castanopsis concinna* | Tree | 0.978 | 0.977 | 0.975 | 0.975 | 0.969 | 0.947 |
| *Phellodendron amurense* | Tree | 0.950 | 0.946 | 0.929 | 0.956 | 0.930 | 0.903 |
| *Pseudotsuga sinensis* | Tree | 0.773 | 0.787 | 0.705 | 0.799 | 0.715 | 0.557 |
| *Cibotium barometz* | Fern | 0.928 | 0.929 | 0.910 | 0.934 | 0.919 | 0.893 |
| *Pseudolarix amabilis* | Tree | 0.870 | 0.879 | 0.848 | 0.891 | 0.845 | 0.823 |
| *Fagopyrum dibotrys* | Herb | 0.873 | 0.866 | 0.817 | 0.876 | 0.839 | 0.737 |
| *Zelkova schneideriana* | Tree | 0.846 | 0.854 | 0.786 | 0.846 | 0.799 | 0.688 |
| *Cercidiphyllum japonicum* | Tree | 0.810 | 0.807 | 0.756 | 0.812 | 0.749 | 0.550 |
| *Nelumbo nucifera* | Herb | 0.969 | 0.957 | 0.960 | 0.972 | 0.962 | 0.958 |
| *Rhoiptelea chiliantha* | Tree | 0.984 | 0.984 | 0.985 | 0.984 | 0.981 | 0.983 |
| *Toona ciliata* var. *pubescens* | Tree | 0.976 | 0.978 | 0.977 | 0.980 | 0.976 | 0.975 |
| *Phoebe bournei* | Tree | 0.879 | 0.881 | 0.834 | 0.874 | 0.846 | 0.766 |
| *Aldrovanda vesiculosa* | Herb | 0.905 | 0.854 | 0.827 | 0.908 | 0.818 | 0.646 |
| *Taxus wallichiana* var. *mairei* | Tree | 0.870 | 0.863 | 0.819 | 0.871 | 0.825 | 0.730 |
| *Phoebe zhennan* | Tree | 0.642 | 0.622 | 0.536 | 0.662 | 0.554 | 0.393 |
| *Abies chensiensis* | Tree | 0.795 | 0.759 | 0.721 | 0.799 | 0.709 | 0.511 |
| *Zenia insignis* | Tree | 0.952 | 0.955 | 0.963 | 0.942 | 0.937 | 0.934 |
| *Machilus nanmu* | Tree | 0.963 | 0.947 | 0.949 | 0.963 | 0.950 | 0.914 |
| *Eurycorymbus cavaleriei* | Tree | 0.835 | 0.851 | 0.796 | 0.839 | 0.814 | 0.720 |
| *Euchresta japonica* | Shrub | 0.944 | 0.942 | 0.921 | 0.941 | 0.924 | 0.893 |
| *Anisodus tanguticus* | Herb | 0.786 | 0.758 | 0.707 | 0.786 | 0.696 | 0.532 |
| *Dipentodon sinicus* | Shrub | 0.997 | 0.995 | 0.994 | 0.997 | 0.995 | 0.992 |
| *Ceratopteris thalictroides* | Fern | 0.984 | 0.982 | 0.977 | 0.978 | 0.972 | 0.957 |
| [*Tetracentron sinense*](http://foc.eflora.cn/content.aspx?TaxonId=200008490) | Tree | 0.856 | 0.837 | 0.803 | 0.840 | 0.813 | 0.648 |
| *Fraxinus mandschurica* | Tree | 0.890 | 0.875 | 0.856 | 0.885 | 0.841 | 0.774 |
| [*Metasequoia glyptostroboides*](http://foc.eflora.cn/content.aspx?TaxonId=200005396) | Tree | 0.881 | 0.863 | 0.837 | 0.885 | 0.828 | 0.748 |
| *Larix mastersiana* | Tree | 0.958 | 0.955 | 0.963 | 0.959 | 0.951 | 0.955 |
| *Brainea insignis* | Fern | 0.985 | 0.985 | 0.982 | 0.984 | 0.980 | 0.969 |
| *Malania oleifera* | Tree | 0.906 | 0.905 | 0.905 | 0.906 | 0.905 | 0.905 |
| *Alsophila spinulosa* | Fern | 0.974 | 0.972 | 0.982 | 0.971 | 0.967 | 0.965 |
| *Taiwania cryptomerioides* | Tree | 0.938 | 0.941 | 0.930 | 0.946 | 0.931 | 0.899 |
| *Cinnamomum japonicum* | Tree | 0.971 | 0.968 | 0.955 | 0.959 | 0.976 | 0.939 |
| *Myriophyllum ussuriense* | Herb | 0.978 | 0.978 | 0.976 | 0.980 | 0.976 | 0.969 |
| *Oyama wilsonii* | Shrub | 0.992 | 0.990 | 0.987 | 0.990 | 0.987 | 0.974 |
| *Camptotheca acuminata* | Tree | 0.899 | 0.905 | 0.859 | 0.878 | 0.857 | 0.772 |
| *Emmenopterys henryi* | Tree | 0.824 | 0.817 | 0.763 | 0.829 | 0.774 | 0.663 |
| *Alsophila metteniana* | Fern | 0.940 | 0.928 | 0.911 | 0.937 | 0.905 | 0.861 |
| *Triaenophora rupestris* | Herb | 0.700 | 0.673 | 0.645 | 0.709 | 0.646 | 0.535 |
| *Glycine soja* | Herb | 0.881 | 0.872 | 0.853 | 0.885 | 0.841 | 0.785 |
| *Ginkgo biloba* | Tree | 0.820 | 0.813 | 0.779 | 0.826 | 0.779 | 0.676 |
| *Picea brachytyla* var. *complanata* | Tree | 0.871 | 0.853 | 0.802 | 0.872 | 0.832 | 0.688 |
| *Cinnamomum longepaniculatum* | Tree | 0.899 | 0.875 | 0.848 | 0.892 | 0.856 | 0.741 |
| *Cinnamomum camphora* | Tree | 0.878 | 0.876 | 0.865 | 0.890 | 0.863 | 0.844 |
| *Phoebe chekiangensis* | Tree | 0.985 | 0.984 | 0.978 | 0.977 | 0.977 | 0.974 |
| *Zoysia sinica* | Herb | 0.930 | 0.882 | 0.839 | 0.854 | 0.857 | 0.855 |
| *Platycrater arguta* | Shrub | 0.979 | 0.980 | 0.978 | 0.977 | 0.974 | 0.974 |
| *Acer amplum* subsp. *catalpifolium* | Tree | 0.966 | 0.956 | 0.952 | 0.960 | 0.945 | 0.911 |
| *Tilia amurensis* | Tree | 0.938 | 0.918 | 0.904 | 0.933 | 0.904 | 0.864 |
| *Madhuca pasquieri* | Tree | 0.967 | 0.970 | 0.965 | 0.969 | 0.965 | 0.955 |
| *Chosenia arbutifolia* | Tree | 0.907 | 0.882 | 0.861 | 0.917 | 0.854 | 0.733 |
