# Supplementary material for: Climate change may threaten habitat suitability of threatened plant species within Chinese nature reserves
Source: PeerJ. 2016 Jun 14;4:e2091. doi: 10.7717/peerj.2091 (PMC4911960; doi:10.7717/peerj.2091)
Supplement: Table S8 [file peerj-04-2091-s008.docx]

**Table S8.** Summary of overlap between current and future climatically suitable habitats for threatened plant species organized by nature reserve.

| Nature | 2050s-Low | 2050s-Medium | 2050s-High | 2080s-Low | 2080s-Medium | 2080s-High |
| --- | --- | --- | --- | --- | --- | --- |
| Banqiao | 0.962 | 0.943 | 0.911 | 0.956 | 0.912 | 0.803 |
| Gujingyuan | 0.921 | 0.893 | 0.867 | 0.919 | 0.855 | 0.777 |
| Qingliangfeng | 0.879 | 0.868 | 0.827 | 0.872 | 0.826 | 0.686 |
| Songshan | 0.935 | 0.959 | 0.903 | 0.948 | 0.926 | 0.877 |
| Daiyunshan | 0.800 | 0.787 | 0.739 | 0.777 | 0.759 | 0.657 |
| E'meifeng | 0.832 | 0.832 | 0.746 | 0.797 | 0.771 | 0.621 |
| Longqishan | 0.882 | 0.891 | 0.838 | 0.871 | 0.859 | 0.761 |
| Minjiangyuan | 0.843 | 0.829 | 0.784 | 0.827 | 0.455 | 0.747 |
| Tingjiangyuan | 0.773 | 0.754 | 0.687 | 0.718 | 0.700 | 0.578 |
| Xiongjianghuangchulin | 0.811 | 0.806 | 0.769 | 0.817 | 0.787 | 0.673 |
| Lianhuashan | 0.920 | 0.926 | 0.943 | 0.920 | 0.924 | 0.966 |
| Qinzhouzhenxishuishengyeshengdongwu | 0.852 | 0.810 | 0.792 | 0.855 | 0.801 | 0.647 |
| Taizishan | 0.977 | 0.973 | 0.968 | 0.978 | 0.968 | 0.933 |
| Yuhe | 0.850 | 0.809 | 0.776 | 0.846 | 0.787 | 0.688 |
| Haifengniaolei | 0.992 | 0.993 | 0.987 | 0.990 | 0.982 | 0.980 |
| Lianzhoutianxin | 0.889 | 0.866 | 0.838 | 0.868 | 0.823 | 0.719 |
| Luokeng'exi | 0.918 | 0.880 | 0.864 | 0.888 | 0.850 | 0.787 |
| Shimentai | 0.871 | 0.831 | 0.828 | 0.843 | 0.798 | 0.742 |
| Xiangtoushan | 0.922 | 0.904 | 0.894 | 0.912 | 0.885 | 0.870 |
| Yunkaishan | 0.943 | 0.920 | 0.922 | 0.930 | 0.894 | 0.875 |
| Bangliangchangbiyuan | 0.876 | 0.900 | 0.885 | 0.905 | 0.861 | 0.834 |
| Chongzuobaitouyehou | 0.941 | 0.919 | 0.909 | 0.937 | 0.879 | 0.875 |
| Daguishan'exi | 0.898 | 0.875 | 0.871 | 0.872 | 0.823 | 0.780 |
| Dayaoshan | 0.890 | 0.885 | 0.850 | 0.863 | 0.823 | 0.769 |
| Encheng | 0.866 | 0.866 | 0.861 | 0.875 | 0.851 | 0.877 |
| Fangchengjinhuacha | 0.941 | 0.934 | 0.941 | 0.935 | 0.900 | 0.928 |
| Huaping | 0.904 | 0.897 | 0.875 | 0.884 | 0.854 | 0.764 |
| Jiuwanshan | 0.829 | 0.838 | 0.800 | 0.831 | 0.781 | 0.713 |
| Qichong | 0.834 | 0.818 | 0.780 | 0.812 | 0.763 | 0.708 |
| Shiwandashan | 0.927 | 0.922 | 0.917 | 0.925 | 0.887 | 0.902 |
| Yinzhulaoshanziyuanlengshan | 0.933 | 0.931 | 0.934 | 0.935 | 0.905 | 0.839 |
| Yuanbaoshan | 0.832 | 0.845 | 0.792 | 0.834 | 0.782 | 0.697 |
| Dashahe | 0.773 | 0.771 | 0.711 | 0.778 | 0.726 | 0.598 |
| Fodingshan | 0.801 | 0.829 | 0.767 | 0.828 | 0.795 | 0.711 |
| Leigongshan | 0.849 | 0.876 | 0.817 | 0.880 | 0.820 | 0.692 |
| Yinggeling | 0.911 | 0.897 | 0.881 | 0.898 | 0.880 | 0.865 |
| Changlihuangjinhai'an | 0.994 | 0.881 | 0.874 | 0.870 | 0.780 | 0.692 |
| Qingyazhai | 0.872 | 0.845 | 0.828 | 0.874 | 0.830 | 0.773 |
| Tuoliang | 0.975 | 0.943 | 0.940 | 0.967 | 0.943 | 0.880 |
| Xiaowutaishan | 0.871 | 0.875 | 0.833 | 0.900 | 0.849 | 0.784 |
| Baotianman | 0.825 | 0.781 | 0.758 | 0.820 | 0.758 | 0.607 |
| He'nandabieshan | 0.935 | 0.928 | 0.884 | 0.936 | 0.887 | 0.781 |
| Gaoleshan | 0.789 | 0.747 | 0.655 | 0.779 | 0.680 | 0.546 |
| Huangheshidi | 0.863 | 0.859 | 0.860 | 0.865 | 0.868 | 0.691 |
| Jigongshan | 0.798 | 0.763 | 0.715 | 0.787 | 0.717 | 0.571 |
| Beijicun | 0.888 | 0.888 | 0.865 | 0.896 | 0.888 | 0.827 |
| Chuonahe | 0.897 | 0.891 | 0.884 | 0.903 | 0.892 | 0.859 |
| Daxiagu | 0.927 | 0.907 | 0.904 | 0.932 | 0.888 | 0.820 |
| Dongbeihu | 0.966 | 0.954 | 0.934 | 0.968 | 0.935 | 0.851 |
| Dongfanghong | 0.962 | 0.932 | 0.912 | 0.959 | 0.914 | 0.821 |
| Duobuku'er | 0.960 | 0.969 | 0.969 | 0.965 | 0.963 | 0.860 |
| Fenglin | 0.952 | 0.943 | 0.917 | 0.952 | 0.925 | 0.823 |
| Fenghuangshan | 0.938 | 0.916 | 0.898 | 0.939 | 0.893 | 0.826 |
| Gongbielahe | 0.983 | 0.989 | 0.971 | 0.987 | 0.992 | 0.909 |
| Lingfeng | 0.966 | 0.954 | 0.955 | 0.962 | 0.960 | 0.952 |
| Maolan'gou | 0.976 | 0.981 | 0.917 | 0.978 | 0.979 | 0.921 |
| Mingshui | 0.888 | 0.953 | 0.955 | 0.929 | 0.884 | 0.895 |
| Mudanfeng | 0.953 | 0.939 | 0.925 | 0.956 | 0.910 | 0.815 |
| Pingdingshan | 0.957 | 0.942 | 0.922 | 0.956 | 0.921 | 0.851 |
| Qixingfengdongbeihu | 0.940 | 0.928 | 0.906 | 0.944 | 0.898 | 0.795 |
| Sanhuanpao | 0.910 | 0.879 | 0.857 | 0.916 | 0.854 | 0.757 |
| Shankou | 0.954 | 0.958 | 0.960 | 0.958 | 0.962 | 0.924 |
| Taipinggou | 0.980 | 0.977 | 0.968 | 0.989 | 0.965 | 0.883 |
| Wuyiling | 0.970 | 0.965 | 0.941 | 0.972 | 0.952 | 0.886 |
| Wuyu'erhe | 0.941 | 0.884 | 0.833 | 0.934 | 0.930 | 0.885 |
| Wudalianchihuoshandizhiyiji | 0.953 | 0.957 | 0.955 | 0.961 | 0.967 | 0.947 |
| Xiaobeihu | 0.967 | 0.955 | 0.941 | 0.969 | 0.935 | 0.842 |
| Xinqingbaitouhe | 0.975 | 0.979 | 0.967 | 0.977 | 0.971 | 0.903 |
| Youhao | 0.943 | 0.930 | 0.910 | 0.945 | 0.923 | 0.861 |
| Zhongyangzhanheizuisongji | 0.872 | 0.873 | 0.846 | 0.869 | 0.863 | 0.834 |
| Badongjinsihou | 0.869 | 0.850 | 0.819 | 0.873 | 0.815 | 0.652 |
| Duheyuan | 0.893 | 0.875 | 0.844 | 0.899 | 0.852 | 0.695 |
| Hubeidabieshan | 0.900 | 0.887 | 0.838 | 0.893 | 0.835 | 0.720 |
| Mulinzi | 0.941 | 0.939 | 0.904 | 0.935 | 0.891 | 0.722 |
| Nanhe | 0.760 | 0.743 | 0.700 | 0.774 | 0.692 | 0.639 |
| Qizimeishan | 0.687 | 0.702 | 0.625 | 0.697 | 0.650 | 0.504 |
| Saiwudang | 0.810 | 0.793 | 0.749 | 0.829 | 0.760 | 0.659 |
| Sanxiadalaoling | 0.818 | 0.812 | 0.753 | 0.833 | 0.768 | 0.636 |
| Shennongjia | 0.881 | 0.871 | 0.851 | 0.890 | 0.853 | 0.763 |
| Shibalichangxia | 0.935 | 0.926 | 0.898 | 0.939 | 0.902 | 0.796 |
| Wudaoxia | 0.747 | 0.710 | 0.672 | 0.755 | 0.681 | 0.615 |
| Xianfengzhongjianheda'ni | 0.757 | 0.765 | 0.702 | 0.761 | 0.720 | 0.539 |
| Xingdoushan | 0.847 | 0.840 | 0.792 | 0.847 | 0.787 | 0.657 |
| Ye'rengu | 0.867 | 0.848 | 0.814 | 0.880 | 0.809 | 0.693 |
| Baiyunshan | 0.850 | 0.874 | 0.791 | 0.850 | 0.800 | 0.649 |
| Dong'anshunhuangshan | 0.860 | 0.846 | 0.814 | 0.843 | 0.801 | 0.708 |
| Dongdongtinghu | 0.837 | 0.816 | 0.765 | 0.791 | 0.769 | 0.741 |
| Gaowangjie | 0.808 | 0.817 | 0.726 | 0.808 | 0.742 | 0.563 |
| Hupingshan | 0.892 | 0.895 | 0.845 | 0.900 | 0.849 | 0.667 |
| Jintongshan | 0.866 | 0.853 | 0.824 | 0.858 | 0.813 | 0.719 |
| Jiuyishan | 0.926 | 0.920 | 0.902 | 0.919 | 0.895 | 0.816 |
| Wuyunjie | 0.825 | 0.805 | 0.757 | 0.823 | 0.758 | 0.633 |
| Xidongtinghu | 0.912 | 0.919 | 0.896 | 0.911 | 0.891 | 0.859 |
| Baishanyuanshe | 0.939 | 0.872 | 0.873 | 0.949 | 0.841 | 0.716 |
| Boluohu | 0.987 | 0.961 | 0.958 | 0.965 | 0.980 | 0.971 |
| Hunchundongbeihu | 0.930 | 0.900 | 0.868 | 0.931 | 0.864 | 0.748 |
| Ji'an | 0.872 | 0.821 | 0.801 | 0.911 | 0.777 | 0.634 |
| Jingyu | 0.949 | 0.926 | 0.914 | 0.949 | 0.893 | 0.765 |
| Shihu | 0.970 | 0.948 | 0.926 | 0.973 | 0.906 | 0.720 |
| Wangqing | 0.961 | 0.933 | 0.917 | 0.958 | 0.912 | 0.821 |
| Yanminghu | 0.930 | 0.915 | 0.890 | 0.929 | 0.891 | 0.824 |
| Yanchengshidizhenqin | 0.848 | 0.849 | 0.773 | 0.836 | 0.775 | 0.612 |
| Ganjiangyuan | 0.873 | 0.867 | 0.816 | 0.845 | 0.791 | 0.703 |
| Jiulingshan | 0.925 | 0.916 | 0.901 | 0.923 | 0.900 | 0.792 |
| Lushan | 0.806 | 0.790 | 0.762 | 0.820 | 0.763 | 0.683 |
| Qiyunshan | 0.911 | 0.899 | 0.879 | 0.898 | 0.869 | 0.795 |
| Tongboshan | 0.885 | 0.890 | 0.854 | 0.878 | 0.854 | 0.764 |
| Wuyuansenlinniaolei | 0.897 | 0.889 | 0.853 | 0.885 | 0.851 | 0.730 |
| Yangjifeng | 0.864 | 0.859 | 0.805 | 0.846 | 0.810 | 0.689 |
| Bailangshan | 0.891 | 0.906 | 0.833 | 0.936 | 0.911 | 0.914 |
| Daheishan | 0.967 | 0.946 | 0.965 | 0.929 | 0.951 | 0.879 |
| Hongluoshan | 0.942 | 0.926 | 0.778 | 0.925 | 0.821 | 0.802 |
| Louzishan | 0.872 | 0.871 | 0.827 | 0.938 | 0.911 | 0.891 |
| Nulu'erhushan | 0.954 | 0.882 | 0.881 | 0.936 | 0.929 | 0.887 |
| Qinglonghe | 0.869 | 0.915 | 0.806 | 0.854 | 0.910 | 0.818 |
| Yalujiangkoushidi | 0.833 | 0.793 | 0.739 | 0.800 | 0.736 | 0.653 |
| Zhanggutai | 0.915 | 0.876 | 0.852 | 0.931 | 0.841 | 0.706 |
| Alu | 0.940 | 0.941 | 0.942 | 0.942 | 0.947 | 0.940 |
| Bilahe | 0.900 | 0.901 | 0.908 | 0.895 | 0.906 | 0.899 |
| Gaogesitaihanwula | 0.971 | 0.964 | 0.957 | 0.968 | 0.964 | 0.957 |
| Hanshan | 0.870 | 0.865 | 0.865 | 0.860 | 0.860 | 0.861 |
| Hanma | 0.954 | 0.950 | 0.946 | 0.957 | 0.952 | 0.950 |
| Qingshan | 0.963 | 0.957 | 0.916 | 0.969 | 0.959 | 0.904 |
| Wulanba | 0.827 | 0.838 | 0.846 | 0.867 | 0.831 | 0.776 |
| Datongbeichuanheyuanqu | 0.773 | 0.739 | 0.677 | 0.765 | 0.666 | 0.535 |
| Huanghesanjiaozhou | 0.817 | 0.904 | 0.748 | 0.812 | 0.849 | 0.652 |
| Heichashan | 0.849 | 0.851 | 0.826 | 0.853 | 0.832 | 0.791 |
| Lingkongshan | 0.926 | 0.916 | 0.861 | 0.928 | 0.850 | 0.841 |
| Guanyinshan | 0.941 | 0.905 | 0.882 | 0.937 | 0.874 | 0.682 |
| Hanchenghuanglongshanhemaji | 0.901 | 0.886 | 0.874 | 0.903 | 0.868 | 0.841 |
| Huangbaiyuan | 0.939 | 0.923 | 0.906 | 0.937 | 0.891 | 0.789 |
| Huanglongshanhemaji | 0.985 | 0.968 | 0.956 | 0.983 | 0.956 | 0.957 |
| Lueyuangzhenxishuishengdongwu | 0.906 | 0.923 | 0.886 | 0.905 | 0.891 | 0.711 |
| Micangshan | 0.893 | 0.876 | 0.848 | 0.886 | 0.848 | 0.716 |
| Motianling | 0.914 | 0.894 | 0.847 | 0.906 | 0.871 | 0.719 |
| Pingheliang | 0.865 | 0.781 | 0.762 | 0.826 | 0.782 | 0.687 |
| Taibaishan | 0.863 | 0.808 | 0.790 | 0.850 | 0.796 | 0.681 |
| Taibaixushuihe | 0.912 | 0.870 | 0.854 | 0.906 | 0.865 | 0.747 |
| Wuliangshan | 0.809 | 0.785 | 0.764 | 0.792 | 0.737 | 0.682 |
| Zhouzhilaoxiancheng | 0.954 | 0.934 | 0.919 | 0.953 | 0.905 | 0.793 |
| Anzihe | 0.884 | 0.881 | 0.834 | 0.894 | 0.844 | 0.754 |
| Baihe | 0.819 | 0.805 | 0.786 | 0.837 | 0.776 | 0.734 |
| Caopo | 0.864 | 0.868 | 0.854 | 0.856 | 0.845 | 0.746 |
| Gexigou | 0.890 | 0.877 | 0.858 | 0.891 | 0.862 | 0.791 |
| Heizhugou | 0.743 | 0.786 | 0.737 | 0.805 | 0.766 | 0.733 |
| Jiudingshan | 0.852 | 0.839 | 0.772 | 0.876 | 0.797 | 0.632 |
| Laojunshan | 0.708 | 0.711 | 0.615 | 0.735 | 0.657 | 0.492 |
| Liziping | 0.808 | 0.788 | 0.777 | 0.787 | 0.742 | 0.632 |
| Nuoshuihezhenxishuishengdongwu | 0.937 | 0.913 | 0.890 | 0.936 | 0.882 | 0.744 |
| Qianfoshan | 0.860 | 0.846 | 0.815 | 0.854 | 0.822 | 0.710 |
| Xiaozhaizigou | 0.899 | 0.912 | 0.904 | 0.922 | 0.892 | 0.818 |
| Xuebaoding | 0.901 | 0.898 | 0.871 | 0.914 | 0.861 | 0.782 |
| Ailaoshan | 0.790 | 0.909 | 0.831 | 0.924 | 0.761 | 0.879 |
| Daweishan | 0.875 | 0.869 | 0.841 | 0.885 | 0.831 | 0.825 |
| Jiaozishan | 0.783 | 0.828 | 0.766 | 0.831 | 0.834 | 0.781 |
| Lvchunhuanglianshan | 0.888 | 0.899 | 0.874 | 0.908 | 0.858 | 0.832 |
| Nangunhe | 0.869 | 0.877 | 0.848 | 0.881 | 0.844 | 0.805 |
| Tongbiguan | 0.952 | 0.953 | 0.949 | 0.954 | 0.940 | 0.926 |
| Wenshan | 0.841 | 0.871 | 0.795 | 0.894 | 0.826 | 0.709 |
| Wumengshan | 0.795 | 0.782 | 0.761 | 0.804 | 0.753 | 0.636 |
| Yuanjiang | 0.903 | 0.957 | 0.887 | 0.955 | 0.901 | 0.814 |
| Yunlongtianchi | 0.828 | 0.830 | 0.782 | 0.847 | 0.783 | 0.682 |
| Wuyanling | 0.944 | 0.938 | 0.919 | 0.940 | 0.899 | 0.840 |
| Changxingyangzi'e | 0.858 | 0.872 | 0.835 | 0.875 | 0.850 | 0.740 |
| Dabashan | 0.841 | 0.819 | 0.794 | 0.842 | 0.793 | 0.656 |
| Jinfoshan | 0.793 | 0.786 | 0.734 | 0.795 | 0.734 | 0.598 |
| Wulipo | 0.797 | 0.785 | 0.748 | 0.809 | 0.758 | 0.633 |
| Xuebaoshan | 0.858 | 0.839 | 0.810 | 0.857 | 0.808 | 0.693 |
