# Supplementary figures and images for: Climate change may threaten habitat suitability of threatened plant species within Chinese nature reserves
Source: PeerJ. 2016 Jun 14;4:e2091. doi: 10.7717/peerj.2091 (PMC4911960; doi:10.7717/peerj.2091)

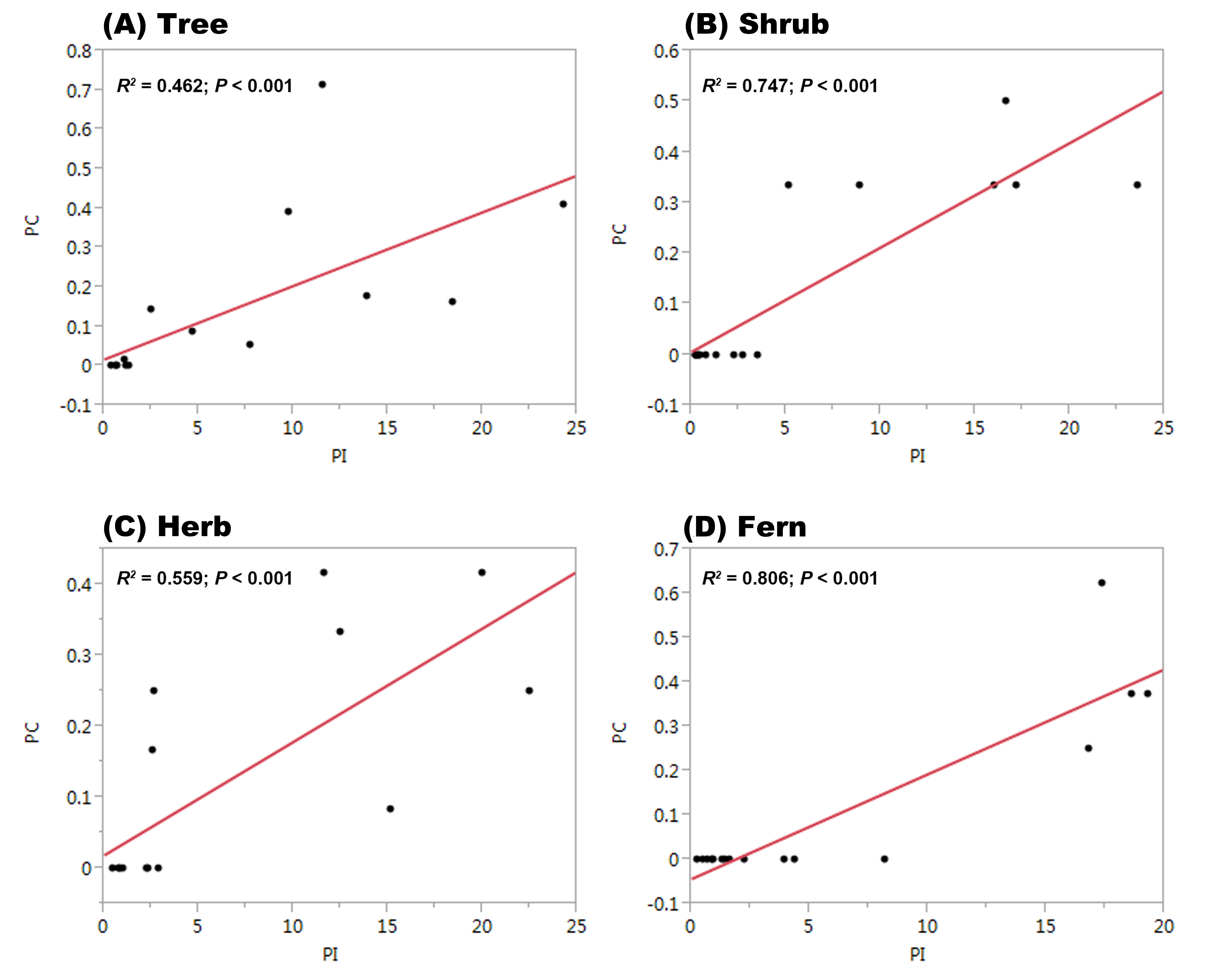

Supplement: Figure S1 [file peerj-04-2091-s009.jpg]

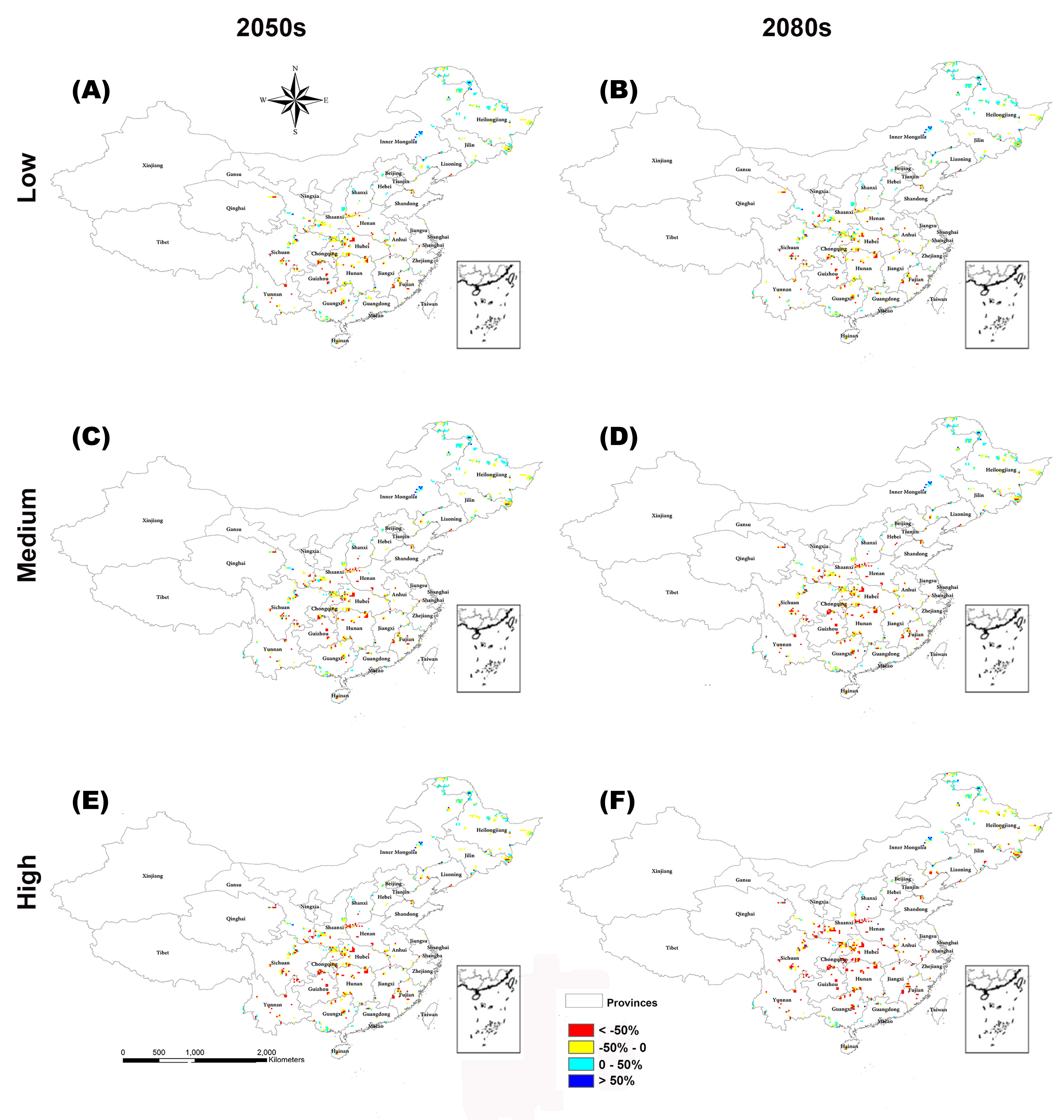

Supplement: Figure S2 [file peerj-04-2091-s010.jpg]
