# Supplementary material for: Climate change may threaten habitat suitability of threatened plant species within Chinese nature reserves
Source: PeerJ. 2016 Jun 14;4:e2091. doi: 10.7717/peerj.2091 (PMC4911960; doi:10.7717/peerj.2091)
Supplement: Data S1 [file peerj-04-2091-s011.docx]

References on nature reserves

| Names of nature reserves | Reference |
| --- | --- |
| Ailaoshan | Institute of Tourism, Yunnan University *et al.* (2012) Ecological Tourism Planning of Yunnan Ailaoshan National Nature Reserve. |
| Alu | Northeast Forestry University *et al.* (2012) Inner Mongolia Alu Nature Reserve Scientific Survey Reports. |
| Anzihe | College of Life Sciences, Sichuan University. (2010) Scientific Survey Report for Sichuan Anzihe Nature Reserve. |
| Badongjinsihou | Hubei Badongjinsihou Nature Reserve Group of the Scientific Inspection. (2013) Hubei Badongjinsihou Nature Reserve Scientific Survey Report. |
| Baihe | College of Life Science, China West Normal University. (2013) Report on Comprehensive Scientific Survey in Baihe Nature Reserve of Sichuan. |
| Bailangshan | Ma WL. (2007) Comprehensive Scientific Investigation Reports on Liaoning Bailangshan Nature Reserve. |
| Baishanyuanshe | Jilin Forestry Survey and Design Research Institute. (2009) Comprehensive Scientific Survey in Jilin Baishanyuanshe Nature Reserve. |
| Baiyunshan | Li DQ, Su XJ. (2010) Comprehensive Scientific Survey Report of Hunan Baiyunshan Nature Reserve. |
| Bangliangchangbiyuan | Guangxi Forestry Survey and Design Institute. (2010) Investigation Report of Guangxi Bangliangchangbiyuan Natural Reserve. |
| Banqiao | Zhang GF. (2007) Plant Biodiversity of Banqiao Natural Reserve in Anhui Province. Nanjing: Nanjing Normal University Press. |
| Baotianman | Song CS. (1994) Scientific Survey of Henan Baotianman National Nature Reserve. Beijing: China Forestry Press. |
| Beijicun | Northeast Forestry University *et al.* (2013) Comprehensive Investigation Report of Heilongjiang Beijicun Nature Reserve. |
| Bilahe | Northeast Forestry University *et al.* (2011) Inner Mongolia Bilahe Nature Reserve Scientific Survey Reports. |
| Boluohu | Jilin Boluohu Nature Reserve Group of the Scientific Inspection. (2009) The Report of Science Survey for Boluo Lake Nature Reserve of Jilin Province. |
| Caopo | College of Life Science, China West Normal University. (2013) Report on Comprehensive Scientific Survey in Caopo Nature Reserve of Sichuan. |
| Changlihuangjinhai'an | Hebei Normal University. (2012) Scientific Survey Report of Hebei Changli Gold Coast Nature Reserve. |
| Changxingyangzi'e | Research Institute of Subtropical Forestry, Chinses Academy of Forestry *et al.* (2013) Scientific Investigation Report of Zhejiang Changxing Nature Reserve of Chinese Alligator. |
| Chongzuobaitouyehou | The Forestry Department of Guangxi Autonomous Region. (2010) Scientific Survey Report of Guangxi Chongzuo *Trachypithecus poliocephalus* Nature Reserve. |
| Chuonahe | College of Wildlife Resources, Northeast Forestry University. (2007) Comprehensive Investigation Report of Heilongjiang Chuonahe Nature Reserve. |
| Dabashan | Chongqing Institute of Medicinal Plant Cultivation *et al.* (2008) Comprehensive Investigation Report on the Scope And Function Adjustment of Chongqing Dabashan National Nature Reserve. |
| Dafengmilu | Nanjing Forestry University *et al.* (2012) Report on Comprehensive Scientific Survey in Dafeng *Elaphurus davidianus* Nature Reserve of Jiangsu. |
| Daguishan'exi | Zhang JJ, Yu BC. (2010) Scientific Survey Report of Guangxi Daguishan Chinese Xenosaurs Nature Reserve. |
| Daheishan | Liaoning Daheishan Nature Reserve Group of the Scientific Inspection. (2011) Liaoning Daheishan Nature Reserve Scientific Survey Report. |
| Daiyunshan | Lin P. (2003) Fujian Daiyunshan Natural Reserve Scientific Integrated Survey Report. Xiamen: Xiamen University Press. |
| Dashahe | Guizhou University. (2011) Comprehensive Investigation Report of Guizhou Dashahe Nature Reserve. |
| Datongbeichuanheyuanqu | Qinghai Datongbeichuanheyuanqu Nature Reserve Group of the Scientific Inspection. (2012) Qinghai Datongbeichuanheyuanqu Nature Reserve Scientific Survey Report. |
| Daweishan | Southwest Forestry University *et al.* (1999) Scientific Survey Report of Yunnan Daweishan Nature Reserve. |
| Daxiagu | Northeast Forestry University *et al.* (2012) Comprehensive Investigation Report of Heilongjiang Daxiagu Natural Reserve. |
| Dayaoshan | Kunming Forestry Survey and Design Institute, State Forestry Administration et al. (2011) Master Plan of Guangxi Dayaoshan National Nature Reserve. |
| Dong'anshunhuangshan | Central South University of Forestry and Technology *et al.* (2010) Comprehensive Scientific Survey Report of Hunan Dong'anshunhuangshan Nature Reserve. |
| Dongdongtinghu | Central South Forestry Survey and Design Institute, State Forestry Bureau. (2013) Comprehensive Scientific Investigation Report on the Scope and Function of the National Nature Reserve of Hunan East Dongting Lake. |
| Dongfanghong | College of Wildlife Resources, Northeast Forestry University. (2014) Scientific Survey of Heilongjiang Dongfanghong Nature Reserve. |
| Duheyuan | Hubei Duheyuan Nature Reserve Group of the Scientific Inspection. (2010) Scientific Survey Report on Duheyuan Nature Reserve of Hubei Province. |
| Duobuku'er | College of Wildlife Resources, Northeast Forestry University. (2009) Scientific Survey of Heilongjiang Duobuku'er Nature Reserve. |
| E'meifeng | Xiamen University. (2013) Fujian E'meifeng Natural Reserve Scientific Integrated Survey Report. |
| Encheng | Guangxi Encheng Nature Reserve Group of the Scientific Inspection. (2011) Scientific Survey Report of Guangxi Encheng Nature Reserve. |
| Fangchengjinhuacha | Guangxi Forestry Survey and Design Institute. (2010) Scientific Survey of Guangxi Fangchengjinhuacha National Nature Reserve. |
| Fenghuangshan | Northeast Forestry University. (2011) Heilongjiang Fenghuangshan Nature Reserve Scientific Survey. |
| Fenglin | State Forestry Bureaue Survey Scheme Designing Institute *et al*. (2013) Construction Implementation Plan of Heilongjiang Fenglin National Nature Reserve. |
| Fodingshan | Forestry Department of Guizhou Province *et al.* (2013) Comprehensive Investigation Report of Guizhou Fodingshan Nature Reserve. |
| Ganjiangyuan | Editorial Committee of Comprehensive Scientific Investigation Reports on Jiangxi Ganjiangyuan Nature Reserve. (2010) Comprehensive Scientific Investigation Reports on Jiangxi Ganjiangyuan Nature Reserve. |
| Gaogesitaihanwula | Inner Mongolia Forestry Survey and Design Institute. (2005) Comprehensive Scientific Investigation Reports on Inner Mongolia Gaogesitaihanwula Nature Reserve. |
| Gaoleshan | Ye YZ, Wang JT. (2013) Scientific Survey of Henan Gaoleshan Nature Reserve. Beijing: Science Press. |
| Gaowangjie | Central South Forestry Survey and Design Institute, State Forestry Bureau *et al.* (2006) Scientific Survey Report of Hunan Gaowangjie Nature Reserve. |
| Gexigou | Sichuan Forestry Science Research Institute. (2010) Natural Protection Area Planning of Sichuan Gexigou Nature Reserve. |
| Gongbielahe | Northeast Forestry University. (2014) Heilongjiang Gongbiela River Nature Reserve Scientific Survey. |
| Guanyinshan | Dang KL, Li DW. (2006) The Comprehensive Scientific Investigation and Biodiversity Research of the Guanyin Mountain Nature Reserve in Shaanxi. Beijing: China Forestry Press. |
| Gujingyuan | State Forestry Bureaue Survey Scheme Designing Institute. (2013) Anhui Gujingyuan Natural Reserve Scientific Integrated Survey Report. |
| Haifengniaolei | Sun Yat-sen University. (2006) Comprehensive Investigation Report of Bird Natural Reserve in Haifeng County, Guangdong Province. |
| Hanchenghuanglongshanhemaji | Zhang FC, Yang XZ. (2006) Comprehensive Scientific Investigation Reports on Shaanxi Hanchenghuanglong Mountain *Crossoptilon mantchuricum* Nature Reserve. Shaanxi: Shaanxi Science and Technology Press. |
| Hanma | State Forestry Bureaue Survey Scheme Designing Institute. (2012) Construction Implementation Plan of Inner Mongolia Hanma National Nature Reserve. |
| Hanshan | Inner Mongolia Second Forestry Monitoring and Planning Institute. (2012) Comprehensive Scientific Investigation Reports on Inner Mongolia Hanshan Nature Reserve. |
| Heichashan | College of Environment and Resources, Shanxi University *et al.* (2009) Comprehensive Scientific Investigation Reports on Shanxi Heichashan Nature Reserve. |
| Heizhugou | College of Life Sciences, Sichuan University *et al.* (2005) Scientific Survey Report for Sichuan Heizhugou Nature Reserve. |
| He'nandabieshan | Ye YZ, Zhuo WH, Zheng XX. (2012) Scientific Survey Report of Henan Dabieshan Nature Reserve. Beijing: Science Press. |
| Hengshuihu | Beijing Forestry University *et al*. (2010) Range Adjustment of Scientific Survey Report of Hebei Hengshui Lake National Nature Reserve. |
| Hongluoshan | Liaoning Hongluoshan Nature Reserve Group of the Scientific Inspection. (2011) Comprehensive Scientific Investigation Reports on Liaoning Hongluoshan Nature Reserve. |
| Huangbaiyuan | Dang KL, Li DW. (2009) Comprehensive Scientific Investigation Reports on Shaanxi Huangbaiyuan Nature Reserve. Shaanxi: Northwest A&F University Press. |
| Huanghesanjiaozhou | Beijing Normal University *et al.* (2010) Comprehensive Demonstration Report on Adjustment of Function Area of The Yellow River Delta National Nature Reserve in Shandong. |
| Huangheshidi | Henan Forestry Department. (2001) Scientific Survey of Henan Yellow River Wetland Nature Reserve. Beijing: China Environmental Press. |
| Huanglongshanhemaji | Li WZ, Zhao PX, Jia SP. (2006) The Overall Planning of Shaanxi Huanglong Mountain *Crossoptilon mantchuricum* Nature Reserve. |
| Huaping | State Forestry Bureaue Survey Scheme Designing Institute. (2014) Scientific Survey of Guangxi Huaping National Nature Reserve. |
| Hubeidabieshan | Planning and Design Institute of Forest Products Industry, State Foresty Administration. (2012) Study Scientific Survey of Dabieshan Nature Reserve in Hubei Province. |
| Hunchundongbeihu | Jilin Forestry Survey and Design Research Institute. (2013) Comprehensive Scientific Survey Report in Jilin Hunchun National Nature Reserve. |
| Hupingshan | Planning and Design Institute of Forest Products Industry, State Foresty Administration *et al*. (2011) The Comprehensive Scientific Investigation Report on the Adjustment of the Function Area of Hunan Hupingshan National Nature Reserve. |
| Ji'an | Jilin Forestry Survey and Planning Institute *et al.* (2012) Comprehensive Scientific Survey Report in Jilin Ji'an National Nature Reserve. |
| Jiaozishan | Kunming Forestry Survey and Design Institute, State Forestry Bureau. (2009) The Overall Planning of Yunnan Jiaozishan Nature Reserve. |
| Jigongshan | Song CS. (1994) Scientific Survey of He'nan Jigong Mountian China National Nature Reserve. Beijing: China Forestry Press. |
| Jinfoshan | State Forestry Bureaue Survey Scheme Designing Institute *et al*. (2012) The Comprehensive Scientific Investigation Report on the Adjustment of the Scope and Function Area of Chongqing Jinfoshan National Nature Reserve. |
| Jingyu | Jilin Resources Department. (2009) Comprehensive Scientific Survey Report in Jilin Jingyu National Nature Reserve. |
| Jintongshan | Deng XJ, Jiang ZG. (2010) Comprehensive Scientific Survey Report of Hunan Jintongshan Nature Reserve. |
| Jiudingshan | College of Life Sciences, Sichuan University *et al.* (2012) Scientific Survey Report for Sichuan Jiudingshan Nature Reserve. |
| Jiulingshan | State Forestry Bureaue Survey Scheme Designing Institute *et al*. (2008) Jiangxi Jiulingshan Nature Reserve Master Plan. |
| Jiushanliedao | Cai YH, Zhang HB. (2007) Comprehensive Scientific Investigation Report of Zhejiang Jiushan Islands Marine Ecological Nature Reserve. |
| Jiuwanshan | Guangxi Forestry Survey and Design Institute. (2014) The Comprehensive Scientific Investigation Report on the Adjustment of the Scope and Function Area of Guangxi Jiuwanshan National Nature Reserve. |
| Jiuyishan | Central South University of Forestry and Technology *et al.* (2011) Comprehensive Scientific Survey Report in Hunan Jiuyishan Nature Reserve. |
| Laojunshan | College of Life Sciences, Sichuan University. (2007) Scientific Survey Report for Laojunshan Nature Reserve in Pingshan County, Sichuan Province. |
| Laoyelingdongbeihu | Feline Research Center, the State Forestry Administration *et al.* (2012) Heilongjiang Laoyeling Amur Tiger Nature Reserve Scientific Investigation Report. |
| Leigongshan | State Forestry Bureaue Survey Scheme Designing Institute *et al*.(2009) Master Plan of Guizhou Leigongshan National Nature Reserve. |
| Lianhuashan | State Forestry Bureaue Survey Scheme Designing Institute. (2012) Construction Implementation Plan of Gansu Lianhuashan National Natural Reserve. |
| Lianzhoutianxin | Guangzhou Lianzhoutianxin Nature Reserve Roup of the Scientific Inspection. (2011) Comprehensive Investigation Report of Guangzhou Lianzhoutianxin Natural Reserve. |
| Lingfeng | Daxing'an Mountains Forestry Survey and Design Institute, State Forestry Bureau. (2013) Comprehensive Investigation Reports on Heilongjiang Lingfeng Nature Reserve. |
| Lingkongshan | Shanxi University *et al.* (2012) Comprehensive Scientific Investigation Reports on Shanxi Lingkongshan Nature Reserve. |
| Liziping | Institute of Rare Animal and Plant Research, China West Normal University. (2012) The Scientific Survey Report on Liziping Nature Reserve in Sichuan Province, China. |
| Longqishan | Li ZY. (1994) Plants of Longqishan Mountain, Fujian, China. Beijing: Science and Technology of China Press |
| Louzishan | Liaoning Louzishan Nature Reserve Group of the Scientific Inspection. (2013) Comprehensive Scientific Investigation Reports on Liaoning Louzishan Nature Reserve. |
| Lueyangzhenxishuishengdongwu | Wang GY. (2010) Report of Comprehensive Investigation of Nature Reserve in Shaanxi Lueyang Rare Aquatic Animal. |
| Luokeng'exi | Guangzhou University *et al.* (2006) Scientific Survey Report of Luokeng *Shinisaurus crocodilurus* Nature Reserve in Guangdong Province. |
| Lushan | Jiangxi Lushan Nature Reserve. (2010) Comprehensive Scientific Investigation Reports on Jiangxi Lushan Nature Reserve. |
| Lvchunhuanglianshan | Kunming Insitute of Botany, Chinese Academy of Sciences *et al.* (2001) Scientific Survey of Yunnan Lvchunhuanglianshan Nature Reserve. |
| Maolan'gou | Northeast Forestry University. (2011) Heilongjiang Maolan'gou Nature Reserve Scientific Survey. |
| Micangshan | Ren Y, Wen ZQ. (2008) Comprehensive Scientific Investigation Reports on Shaanxi Micangshan Nature Reserve. Beijing: Science Press. |
| Mingshui | Northeast Forestry University. (2011) Heilongjiang Mingshui Nature Reserve Scientific Survey Report. |
| Minjianghekoushidi | Yu X. (2011) Natural Reserve Scientific Integrated Survey Report of Fujian Minjiang Estuary Wetland. |
| Minjiangyuan | Fujian Forestry Survey and Planning Institute. (2012) Scientific Integrated Survey Report of Fujian Minjiangyuan National Nature Reserve. |
| Motianling | Guo WY, Dang KL. (2007) Comprehensive Rcientific Investigation and Research on Shaanxi Motianling Nature Reserve. Shaanxi: Shaanxi Science and Technology Press |
| Mudanfeng | Northeast Forestry University *et al.* (2011) Comprehensive Investigation Reports on Heilongjiang Mudanfeng National Nature Reserve. |
| Mulinzi | Ge JW, Hu HX. (2009) Scientific Survey and Study on Forest Biodiversity in Mulinzi Nature Reserve.of Hubei, Central China. Beijing: Science Press. |
| Nangunhe | Yang YM, Du F. (2001) Nangun River National Nature Reserve of China. Kunming: Yunnan Science & Technology Press. |
| Nanhe | College of Resource and Environment, Hubei University *et al.* (2012) Comprehensive Investigation Report of Hubei Nanhe Nature Reserve. |
| Nansihu | State Forestry Bureaue Survey Scheme Designing Institute *et al*. (2011) Comprehensive Scientific Investigation Reports on Shandong Nansi Lake Nature Reserve. |
| Nulu'erhushan | Liaoning Nulu'erhushan National Nature Reserve Group of the Scientific Inspection. (2011) Liaoning Nulu'erhushan National Nature Reserve Scientific Survey Report. |
| Nuoshuihezhenxishuishengdongwu | College of Life Sciences, Sichuan University *et al.* (2009) Scientific Survey Report for Rare Water Animal Nature Reserve in Nuoshuihe, Tongjiang County of Sichuan Province. |
| Pingdingshan | The Third Heilongjiang Forestry Survey and Design Institute. (2013) Comprehensive Investigation Reports on of Heilongjiang Pingdingshan Nature Reserve. |
| Pingheliang | Li ZG, Kang KG. (2008) Comprehensive Scientific Investigation and Plant Diversity of the Provincial Nature Reserve in Shaanxi Pingheliang. Shaanxi: Shaanxi Science and Technology Press. |
| Qianfoshan | College of Life Sciences, Sichuan University. (2012) Scientific Survey Report for Sichuan Qianfoshan Nature Reserve. |
| Qichong | Liu ZL, Lin C. (2012) Guangxi Qichong Nature Reserve Scientific Survey Report. |
| Qingliangfeng | Anhui Academy of Forestry. (2007) Anhui Qingliangfeng Natural Reserve Scientific Integrated Survey Report. |
| Qinglonghe | Luo JC. (2013) Comprehensive Scientific Investigation Reports on Liaoning Qinglonghe Nature Reserve. Beijing: China Forestry Press. |
| Qingshan | Beijing Forestry University *et al.* (2012) Scientific Investigation Report of Inner Mongolia Qingshan Nature Reserve. |
| Qingyazhai | Hebei Normal University *et al.* (2008) Scientific Survey Report of Hebei Qingyazhai Nature Reserve. |
| Qinzhouzhenxishuishengyeshengdongwu | Yangtze River Fisheries Research Institute, Chinese Academy of Fishery Sciences *et al*. (2013) Comprehensive Scientific Investigation Reports on Rare Aquatic Wild Animal Nature Reserve in Qinzhou . |
| Qixingfengdongbeihu | Heilongjiang Institute of Wildlife Research *et al.* (2014) Comprehensive Investigation Reports on Heilongjiang Qixingfeng Nature Reserve. |
| Qiyunshan | Liu XM, Guo YR. (2008) Comprehensive Scientific Investigation Reports on Jiangxi Qiyunshan Nature Reserve. |
| Qizimeishan | Hubei Qizimeishan Nationnal Nature Reserve. (2012) Comprehensive Survey Report on Adjustment of Function Area of Hubei Qizimeishan Nationnal Nature Reserve. |
| Saiwudang | Hubei Saiwudang Nature Reserve Group of the Scientific Inspection. (2008) Hubei Saiwudang Nature Reserve Scientific Survey Report. |
| Sanhuanpao | Heilongjiang Forestry Survey and Design Institute. (2009) Comprehensive Investigation Reports on Heilongjiang Sanhuanpao Nature Reserve. |
| Sanxiadalaoling | College of Urban and Environmental Sciences, Peking University *et al.* (2010) Hubei Sanxiadalaoling Nature Reserve Scientific Investigation and Research Report. |
| Shankou | Wudalianchi Municipal People's Government. (2008) Comprehensive Investigation Reports on Heilongjiang Shankou Nature Reserve. |
| Shedaolaotieshan | Wildlife Research Institute, Northeast Forestry University *et al.* (2009) Comprehensive Scientific Investigation Reports on Liaoning laotieshan Snake Island National Nature Reserve. |
| Shennongjia | State Forestry Bureaue Survey Scheme Designing Institute *et al*. (2011) Construction Implementation Plan of Hubei Shennongjia National Nature Reserve. |
| Shibalichangxia | Gong MH, Lin C. (2011) The Scientific Survey and Research on Shibalichangxia Nature Reserve in Hubei, China. Beijing: Beijing Press. |
| Shihu | Jilin Forestry Survey and Design Research Institute. (2013) Comprehensive Scientific Survey Report in Jilin Shihu Nature Reserve. |
| Shimentai | State Forestry Bureaue Survey Scheme Designing Institute *et al*. (2008) The Overall Planning of Guangdong Shimentai Nature Reserve. |
| Shiwandashan | Tan WF. (2005) Biodiversity and Conservation System of Guangxi Shiwandashan Nature Reserve. Beijing: China Environmental Press. |
| Songshan | Beijing Forestry University. (2011) Scientific Survey of Beijing Songshan National Nature Reserve. |
| Taibaishan | Shaanxi Forestry Survey and Design Institute. (2010) The Overall Planning of Shaanxi Taibai Mountain National Nature Reserve. |
| Taibaixushuihe | Ministry of Agriculture of the People's Republic of China. (2010) Report of Comprehensive Investigation of Shaanxi Taibaixushuihe Nature Reserve For Rare Aquatic Animals. |
| Taipinggou | Northeast Forestry University. (2013) Heilongjiang Taipinggou Nature Reserve Scientific Survey. |
| Taizishan | Yu BC, Sun XG. (2007) Gansu Taizishan Nature Reserve Scientific Survey. |
| Tingjiangyuan | Xiamen University. (2012) Natural Reserve Scientific Integrated Survey Report of Fujian Tingjiangyuan. |
| Tongbiguan | National Plateau Wetlands Research Center. (2011) Scientific Survey Report of Yunnan Tongbiguan Nature Reserve. |
| Tongboshan | Wu YH, Yang GY. (2012) Comprehensive Scientific Investigation Reports on Jiangxi Tongboshan Nature Reserve. |
| Tuoliang | Hebei Forestry Survey and Design Institute *et al*. (2008) Master Plan of Hebei Tuoliang Nature Reserve. |
| Wangqing | School of Urban and Environmental Science, Northeast Normal University *et al.* (2009) Comprehensive Scientific Survey Report in Jilin Wangqing Nature Reserve. |
| Wenshan | Kunming Insitute of Botany, Chinese Academy of Sciences *et al.* (2013) Regional Comprehensive Scientific Investigation Report of Wenshan National Nature Reserve in Yunnan. |
| Wudalianchihuoshandizhiyiji | Wudalianchi Heilongjiang Nature Reserve Management Committee. (2013) Comprehensive Scientific Investigation Report on the Scope and Function of Heilongjiang Wudalianchi Nature Reserve. |
| Wudaoxia | Wang ZX, Cai DJ. (2013) Biodiversity and Conservation of Hubei Wudaoxia Nature Reserve. Beijing: China Forestry Press. |
| Wulanba | Inner Mongolia University *et al.* (2012) Comprehensive Scientific Investigation Reports on Inner Mongolia Wulanba Nature Reserve. |
| Wuliangshan | Kang YX, Gao XB. (2006) Report of Comprehensive Investigation of Shaanxi Wuliangshan Nature Reserve. Shaanxi: Shaanxi Science and Technology Press. |
| Wulipo | Xiao WF, Chen LQ. (2009) Biodiversity of Chongqing Wulipo Nature Reserve. Beijing: China Forestry Press. |
| Wumengshan | Chen BK. (2008) Scientific Survey Report of Yunnan Wumengshan Nature Reserve. |
| Wuyanling | Zhejiang Forestry Survey and Design Institute. (2009) Zhejiang Wuyanling Nationnal Nature Reserve Master Plan. |
| Wuyiling | Planning and Design Institute of Forest Products Industry, State Foresty Administration. (2012) The Adjustment in Scientific Investigation Report of Heilongjiang Wuyiling National Nature Reserve. |
| Wuyuansenlinniaolei | Li ZJ, Ge G. (2010) Comprehensive Scientific Investigation Reports on Wuyuan Forest Bird Nature Reserve in Jiangxi. |
| Wuyu'erhe | Wildlife Research Institute, Northeast Forestry University *et al.* (2011) Comprehensive Investigation Report of Heilongjiang Wuyu'er River Natural Reserve. |
| Wuyunjie | Research Institute of Forest Ecological Environment and Protection, Chinese Academy of Forestry *et al.* (2012) The Comprehensive Scientific Investigation Report on the Adjustment of the Scope and Function Area of Hunan Wuyunjie Natural Neserve. |
| Xianfengzhongjianheda'ni | Hubei Fisheries Science Research Institute. (2009) Comprehensive Investigation Report of Xianfengzhongjianhe *Andrias davidianus* Natural Reserve. |
| Xiangtoushan | State Forestry Bureaue Survey Scheme Designing Institute *et al*. (2010) Guangdong Xiangtoushan Nationnal Nature Reserve Master Plan. |
| Xiaobeihu | Northeast Forestry University. (2009) Heilongjiang Xiaobeihu Nature Reserve Scientific Survey Report. |
| Xiaowutaishan | Hebei Agriculture University *et al.* (2012) Range Adjustment of Scientific Survey Report of Hebei Xiaowutaishan National Nature Reserve. |
| Xiaozhaizigou | Hu JC. (2003) A Report of the Comprehensive Survey on Xiaozhaizi Nature Reserve in Sichuan, China. Sichuan: Sichuan Science and Technology Press. |
| Xidongtinghu | Southern China Institute of Endangered Animals. (2011) Hunan West Dongting Lake Nature Reserve Scientific Survey. |
| Xingdoushan | Liu SX, Qu J. (2003) Scientific Survey of Hubei Xingdoushan Nature Reserve. Hubei: Hubei Science and Technology Press. |
| Xinqingbaitouhe | College of Life Science, Capital Normal University. (2006) Comprehensive Investigation Report of Heilongjiang Xinqing *Grus monacha* Natural Reserve. |
| Xiongjianghuangchulin | Fujian Forestry Survey and Design Institute *et al*. (2009) Master Plan of Fujian Xiongjianghuangchulin Nature Reserve. |
| Xuebaoding | Sichuan Forestry Science Research Institute. (2010) Natural Protection Area Planning of Sichuan Xuebaoding National Nature Reserve. |
| Xuebaoshan | Liu ZL, Huang XY. (2007) Chongqing Xuebaoshan Nature Reserve Scientific Survey Report. |
| Yalujiangkoushidi | Wildlife Research Institute, Northeast Forestry University. (2010) Comprehensive Scientific Investigation Reports on Liaoning Yalujiangkou Wetland National Nature Reserve. |
| Yanchengshidizhenqin | Nanjing Forestry University *et al.* (2011) Report on Comprehensive Scientific Survey for Rare Birds in Jiangsu Yancheng Wetland National Nature Reserve. |
| Yangjifeng | School of Urban and Environmental Sciences, Fudan University. (2008) Jiangxi Yangjifeng Nature Reserve Master Plan. |
| Yanminghu | State Forestry Bureaue Survey Scheme Designing Institute *et al*. (2013) Range Adjustment of Scientific Investigation Report of Jilin Yanminghu National Nature Reserve. |
| Ye'rengu | Wang ZX, He JP. (2013) Biodiversity and Conservation of Hubei Ye'rengu Nature Reserve. Beijing: China Forestry Press. |
| Yinggeling | Jiang HS, Chen BL, Zhou YD, Wang CD, Fang L, Luo YK. (2008) Biodiversity and Conservation of Hainan Yinggeling Nature Reserve. Beijing: China Forestry Press. |
| Yinzhulaoshanziyuanlengshan | Guangxi Forestry Survey and Design Institute. (2013) Scientific Survey of Guangxi Yinzhulaoshanziyuanlengshan Nature Reserve. |
| Youhao | Northeast Forestry University *et al.* (2008) Heilongjiang Youhao Nature Reserve Scientific Survey. |
| Yuanbaoshan | Guangxi Institute of Botany, the Chinese Academy of Sciences. (2011) Comprehensive Investigation Report of Guangxi Yunbaoshan Nature Reserve. |
| Yuanjiang | Chen BK. (2009) Scientific Survey Report of Yunnan Yuanjiang Nature Reserve. |
| Yuhe | State Forestry Bureaue Survey Scheme Designing Institute. (2013) Scientific Investigation Report on Gansu Yuhe Nature Reserve. |
| Yunkaishan | Southern China Institute of Endangered Animals. (2009) Guangdong Yunkaishan Nature Reserve Scientific Survey Reports. |
| Yunlongtianchi | Yunnan Forestry Survey and Design Institute *et al.* (2007) Scientific Survey Report of Yunnan Yunlongtianchi Nature Reserve. |
| Zhanggutai | Liaoning Zhanggutai Nature Reserve. (2008) Liaoning Zhanggutai Nature Reserve Scientific Survey Reports. |
| Zhangjiangkouhongshulinshidi | Lin P. (2001) Scientific Integrated Survey Report of Zhangjiangkou Mangrove Wetland Natural Reserve in Fujian Province. Xiamen: Xiamen University Press. |
| Zhongyangzhanheizuisongji | College of wildlife resources, Northeast Forestry University. (2011) Comprehensive Investigation Report of Heilongjiang Zhongyangzhanheizuisongji Natural Reserve. |
| Zhouzhilaoxiancheng | Jiang ZG. (2006) Biodiversity of Zhouzhilaoxiancheng Nature Reserve, Shaanxi, China. Beijing: Tsinghua University Press. |
